# Discovery of Novel Allosteric Inhibitor Hits for Insulin-Regulated Aminopeptidase Provides Insights on Enzymatic Mechanism

**DOI:** 10.1021/acsomega.5c01169

**Published:** 2025-04-23

**Authors:** Galateia Georgaki, Nikoletta-Maria Koutroumpa, Panagiotis Lagarias, Antreas Afantitis, Athanasios Papakyriakou, Efstratios Stratikos

**Affiliations:** 1 Laboratory of Biochemistry, Department of Chemistry, National and Kapodistrian University of Athens, Zografou 15784, Greece; 2 National Centre for Scientific Research Demokritos, Agia Paraskevi 15341, Greece; 3 NovaMechanics Ltd., Nicosia 1070, Cyprus; 4 School of Chemical Engineering, National Technical University of Athens, Athens 15780, Greece

## Abstract

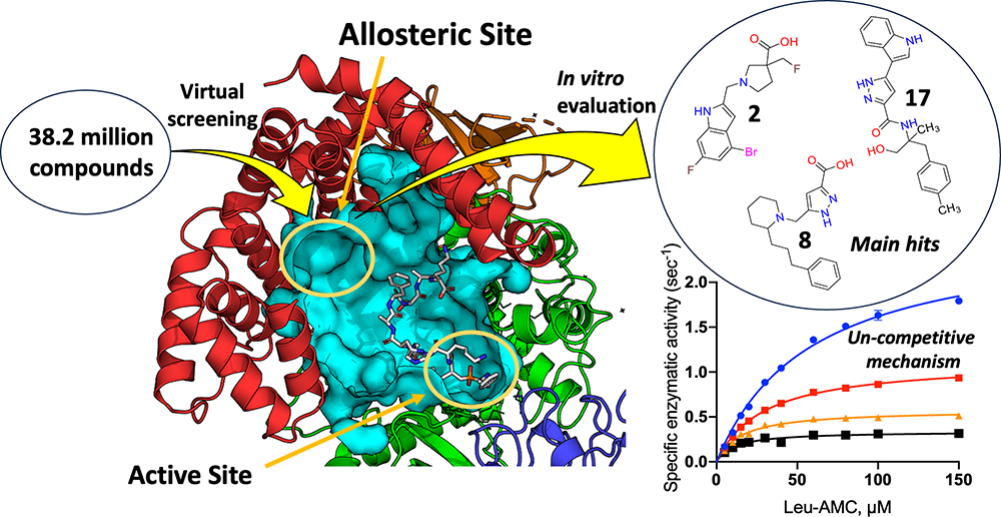

Insulin-regulated aminopeptidase (IRAP) is a transmembrane zinc metalloprotease with various important biological roles, including fibrosis, septic thrombosis, cognitive functions, and immune system regulation. As a result, IRAP is an emerging pharmacological target for several diseases. However, the development of selective inhibitors that specifically regulate its activity remains challenging due to its high sequence and functional homology with many other enzymes that have highly conserved active sites. To circumvent this limitation, we targeted the malate allosteric site, a site that has yielded highly selective inhibitors of the homologous enzyme ERAP1. We performed virtual screening to discover drug-like compounds that bind with high affinity to this allosteric site in IRAP. A database of 38 million diverse, drug-like compounds from ENAMINE was employed for screening at three conformations of the targeted site. A subset of the top-ranked compounds was subsequently evaluated using molecular dynamics simulations and comparative MM/GBSA free energy calculation, from which 17 were selected for further *in vitro* evaluation of their inhibitory activity for IRAP by two orthogonal assays. Three hits, one for each enzyme conformation and substrate class, were selected for further mechanistic evaluation revealing substrate-dependent uncompetitive or noncompetitive mechanisms of action, consistent with the conformationally sensitive nature of the allosteric site. Our results support the tractability of the malate site for the discovery of novel selective IRAP inhibitors, establish novel hits for further development, and suggest that it may be possible to target specific biological functions of IRAP by targeting distinct conformations of the enzyme by allosteric inhibitors.

## Introduction

Insulin-regulated aminopeptidase (IRAP, EC 3.4.11.3) is a transmembrane protein with several important biological functions depending on the tissue and cellular context, including roles in glucose metabolism, fibrosis, cognitive functions, and the immune system.^1^ It consists of an extracellular zinc metalloprotease domain that has aminopeptidase activity, a single transmembrane helix, and a small cytoplasmic domain with roles in endosomal vesicle trafficking. Its aminopeptidase domain can degrade small peptides and by that function plays important roles in biological processes such as the regulation of immune responses,^2,3^ the degradation of placental oxytocin,^4^ and the regulation of oxytocin and vasopressin levels in the brain^5^ as well as cardiac fibrosis,^6^ ischemic stroke,^7^ and septic thrombosis.^8^ As a result, IRAP is an active pharmacological target mainly in regards to its functions in fibrosis, cognition, and immune responses.^6,9,10^ However, no clinical applications of IRAP inhibition have been reported yet, primarily due to limitations of existing compounds and some knowledge gaps in the IRAP mechanism and biology.

The luminal domain of IRAP contains its aminopeptidase function and also serves as the receptor for angiotensin IV.^11,12^ IRAP belongs to the oxytocinase subfamily of M1 aminopeptidases^13^ and carries high sequence and structural homology to the enzymes ER aminopeptidase 1 and 2 (ERAP1 and ERAP2), two intracellular enzymes that also process antigenic peptides.^14^ The aminopeptidase domain consists of four structural domains arranged in a concave structure that forms an extended internal cavity, with limited access to the external solvent.^15^ Upon substrate or ligand binding to the active site, IRAP can change conformation to a more closed configuration in which the internal cavity has no access to the external solvent.^16^ This conformational change has been associated with the peptide trimming mechanism of IRAP and its wide breadth of substrate selectivity.^17^

Several groups have developed inhibitors of IRAP,^18−21^ either based on rational design or library screening. Some of these compounds have been shown to exhibit biological activity in functional assays, such as promoting the formation of dendritic spines in primary hippocampal neuron cultures,^22^ regulating the cross-presentation of antigenic peptides,^21^ glucose tolerance in insulin-resistant Zucker fatty rats,^23^ conferring neuroprotection in a conscious model of ischemic stroke,^7^ and regulating fibrosis.^6^ Most of the reported compounds have been either designed or considered to target the IRAP active site by engaging with the catalytic zinc(II) atom or the adjacent selectivity subsites. Crystallographic studies have demonstrated that active site engagement by inhibitors exploits some unique structural features in IRAP, such as the flexibility of its GAMEN motif.^16,21^ Still, due to the high level of sequence and structural conservation between the many members of the M1 family of aminopeptidases, targeting the active site can present limitations in selectivity and result in off-target effects. In contrast, targeting allosteric sites may result in more selective inhibitors that are amenable to clinical development. In a recent study from our group, we discovered that HFI-419, a widely utilized IRAP inhibitor, is actually allosteric.^17^ However, HFI-419’s unique mechanism of action results in unexpected and complex behavior against different size substrates, limiting its clinical value.^17^ This finding provided renewed inspiration for the exploration of other allosteric sites of IRAP as targets for small-molecule modulators.

IRAP’s aminopeptidase domain is highly homologous (∼49% sequence identity) to ER aminopeptidase 1 (ERAP1), a soluble enzyme that can process antigenic peptide precursors in the ER. ERAP1 plays important roles in the regulation of adaptive immune responses and is currently a target for cancer immunotherapy applications.^24^ ERAP1’s mechanism includes the binding of the C-terminus of a peptidic substrates by an allosteric regulatory site, and this function likely underlies its unique length selection for substrates.^25,26^ In a high-resolution crystal structure of ERAP1, this site was found to be occupied by a malate molecule, a buffer component which mimicked the carboxyl-terminal moiety of a peptide C-terminus.^27^ This malate-binding site (MLT site) has been exploited to generate potent and selective ERAP1 inhibitors.^28,29^ IRAP contains a similar site, although it does not share all structural characteristics and does not share ERAP1’s length-selection properties, suggesting that that site does not have the same function.^30^

In this study, we used structure-based computational screening to discover novel inhibitor hits that target the ERAP-equivalent MLT site in IRAP. We identified several potential hits through evaluation of more than 38 million commercially available compounds from ENAMINE and by molecular dynamics calculations with free energy calculations of selected top-ranked compounds. Aiming to further our understanding of the interplay between IRAP conformations and substrates, we characterized the identified hits for affinity and the mechanism of action using two substrates of different lengths. Our results provide novel hits with potential for further development toward potent IRAP inhibitor leads as well as novel insights on how targeting specific IRAP conformations can be exploited as part of a strategy targeting specific IRAP biological functions.

## Experimental Methods

### Virtual Screening

#### Compound Library

We employed a diversity set of 38.2 million compounds, preselected as a representative 1% of the full ENAMINE REAL Database.^31^ The diversity set contains compounds that have no analogs with a Tanimoto similarity >0.6 within the set (512-bit Morgan2 fingerprint) and have been prefiltered so as to comply with the Lipinski’s and Veber’s criteria (MW ≤500, SlogP ≤5, HBA ≤10, HBD ≤5, rotatable bonds ≤10, and TPSA ≤140).^32,33^ Initially, the compound set was enumerated for tautomeric forms (but restricted to a maximum number of three tautomers per molecule) and assigned the appropriate ionization state of each tautomer at pH ∼ 7.4 using the OpenEye’s program tautomers included in the QUACPAC v.2.0.1.2 suite of applications.^34^ After tautomer enumeration and canonicalization, the diversity set size increased to 41.2 million (an increase of ∼8% from 38,178,008 to 41,244,353 compounds) and was split into 40 × 1.03 million compound chunks for further processing. Stereoisomer enumeration was not performed, and compounds with unspecified stereo centers were converted to a single, randomly selected stereoisomer. 3D conformations of molecules were generated using OMEGA v3.1.1.2^35^ with default parameters for a maximum number of 200 conformers for each molecule and unspecified stereo centers allowed. An average of 158 conformers per compound were generated for the diversity set (6.5 average number of rotatable bonds), except for 44,809 molecules that OMEGA failed to build structure (i.e., ∼0.1% of the total set, mainly due to erroneous stereo center definition in ring atoms), resulting in over 6.5 billion conformers.

#### Target Enzyme

Considering that the exact structure of the target is of paramount importance in structure-based virtual screening, we employed two individual crystal structures of IRAP, one in the fully closed state (PDB ID: 5mj6)^16^ and one in an open state (PDB ID: 4z7i).^30^ The binding site was defined as the internal pocket where a molecule of malic acid was resolved in a high-resolution crystal structure of ERAP1 (PDB ID: 6q4r).^27^ This pocket has been identified as an allosteric site of ERAP1 that accommodated the C-terminus of a transition-state pseudopeptide analogue bound to the internal substrate-binding cavity.^26^ After the X-ray structures of IRAP were superimposed with that of ERAP1, the d-malate molecule (MLT) was transferred to the corresponding pockets of IRAP to facilitate search space detection. In particular, chains A and B of IRAP in the closed conformation exhibited an interacting arginine residue (Arg933) in two alternative conformations that gave rise to different pocket sizes. Therefore, both chains were employed in addition to chain B of IRAP in the open state, which was very similar to chain A (herein designated as IRAP-O). The search space was calculated using the OpenEye’s graphical utility make_receptor v.3.3.0.3 and resulted in three sites with volumes of 984 Å^3^ (IRAP-A), 852 Å^3^ (IRAP-B), and 820 Å^3^ (IRAP-O).

*Virtual screening* was carried out using FRED v.3.3.1.2^36,37^ with default parameters, requesting a hit-list size of 10,000 molecules for each 1.03-million chunk (i.e., ∼1% top-scored compounds). The aggregate 400,000 top-ranked compounds for each docking site were rescored with the Chemgauss4 scoring function using OpenEye’s application ScorePose v3.3.1.2^34^ with the high-resolution optimization (0.5 Å step size for both translation and rotation). In this way, we retrieved the 1800 top-scored compounds, from which we selected a subset of representative and highly potent compounds for experimental evaluation. This was performed by visual investigation of the top-ranked molecules using OpenEye’s VIDA v.4.4.0.4 and by filtering using Enalos Suite.^38^

### Enalos Molecular Property Predictions

The molecular property screening evaluation was performed using the Enalos Suite, a cheminformatics platform which integrates user-friendly tools to make advanced predictive models accessible to the broader scientific community.^38^ All models employed were developed and validated according to the Organization for Economic Cooperation and Development (OECD) guidelines, ensuring the robustness, reliability, and predictive accuracy required for regulatory and scientific applications.^39^ The predictions were performed for five properties essential for assessing drug-likeness and safety: the octanol/water partition coefficient in its logarithmic form (logP), representative of the compound's lipophilicity; water solubility (logS); the bioconcentration factor (logBCF); and mutagenicity and cytotoxicity. These properties were calculated for all the top-scored molecules from each docking cavity (IRAP-A, IRAP-B, and IRAP-O). To narrow down the list of compounds to those that meet key drug-likeness and safety criteria, we applied three filters to the predicted properties: (1) “negative”, which refers to compounds predicted as nonmutagenic, (2) “inactive”, for noncytotoxic, and (3) a lipophilicity range of 1.5 < logP < 3.

### Molecular Dynamics Simulations

Virtual screening of REAL Database hits of ENAMINE^31^ was conducted for the closed structure of the IRAP protein (PDB: 5mj6), chains A and B, and for the open structure (PDB: 4z7i), chain B, as explained above. To investigate the IRAP interactions and to identify differences in the possible selectivity among the compounds considered, molecular dynamics (MD) simulations and binding free energy calculations were performed. The lowest energy conformations of the ligand–IRAP complexes produced by the virtual screening calculations served as the initial structures for the MD simulations. Missing residues in nonterminal regions were added by means of the SWISS-MODEL server,^40^ a web-based integrated service dedicated to protein structure homology modeling, whereas further model refinement was performed using Modeler 1.16.^41−43^ Then, the IRAP protein was described by means of the AMBER14SB^44,45^ force field and according to the protocol proposed by Mpakali et al.^30^ Specifically, a disulfide bond between Cys828 and Cys825 of the receptor was added, the protonation state of the zinc-bound histidines was set to delta-protonation, and hydrogen atoms were added. The ff14SB force-field parameters were applied to the protein atoms, and a simple bonded model was employed for the zinc coordination sphere. Parameters for the ligands considered were calculated using the ANTECHAMBER module of AMBER as implemented, by assigning AM1-BCC partial charges and the General AMBER Force Field (GAFF).^46,47^

Water solvent molecules were explicitly added to all IRAP–ligand complex systems by means of the TIP3P model with a 10 Å water buffer, producing cubic unit cells that included Na+ counterions to ensure charge neutralization.^48^ All MD simulations were performed using the OpenMM 7.4.2^49^ engine. After minimization, each system was heated in constant volume in the canonical (NVT) ensemble until the target temperature of 300 K was reached by using the Langevin integrator. Then, equilibration was conducted in two stages. Initially, a 5 ns simulation was performed in the NVT ensemble by imposing positional restraints on the protein backbone atoms with a harmonic force constant equal to 5 kcal mol^–1^ Å^–1^. The second stage was conducted in the isobaric–isothermal (NPT) ensemble for an additional 5 ns to equilibrate the system density without any restraints on the protein backbone. The Monte Carlo barostat was employed for maintaining constant pressure at 1 atm, and the production simulations were performed for 20 ns for all protein–ligand complexes considered in the NPT ensemble (200 trajectory frames). Upon completion of the production runs, postprocessing was performed within the same node for a series of structural analysis tasks, which include root-mean-square deviation (RMSD), root-mean-square fluctuations (RMSF), hydrogen bond (HB), and clustering analysis, while binding enthalpies by means of the Molecular Mechanics Generalized Born Surface Area (MM-GBSA) method. Specifically, MM-GBSA calculations were conducted every second frame (i.e., 100 frames for each IRAP–ligand complex trajectory) by employing the Hawkins, Cramer, Truhlar pairwise generalized Born model (igb = 1),^50,51^ with recommended settings (i.e., the surface tension proportionality, and the offset values were set to 0.00720 kcal mol^–1^ Å^–2^ and 0.0000 kcal mol^–1^, respectively), while setting the salt molar concentration at 150 mM.

### Recombinant Enzymes

The recombinant extracellular domain of human IRAP was expressed in stably transfected HEK 293S GnTI^(−)^ cell lines as previously described.^30^ The secreted recombinant protein contains an N-terminal Rhodopsin 1D4 tag that allows its isolation from the culture media by loading on Sepharose beads (Cube Biotech) coupled with the anti-1D4 tag antibody (University of British Columbia) and eluting with the 1D4 peptide. The isolated protein was further purified by size exclusion chromatography (Superdex 200 16/60 column; GE Healthcare). The recombinant human enzymes ERAP1 and ERAP2 were produced in Hi5 insect cells with the Baculovirus Expression System (Bac-to-Bac system, Invitrogen) and purified by immobilized-metal affinity chromatography using Ni-NTA agarose beads, as reported previously.^52^

Compounds were purchased from ENAMINE at the 5–10 mg scale and were used without any further purification. Their LC/MS spectra are provided in Figure S3 as Supporting Information. Compounds with chiral centers were provided as mixtures of enantiomers (**2**, **3**, **4**, **8**, **9**, **11**, **12**, **14**, **15**, and **17**), or mixtures of diastereoisomers (**6**, **10**, and **16**), with compound **6** having a relative stereochemistry defined and compound **16** provided in approximately a 1:1 diastereomeric ratio (Supporting Information, Figure S3). Stock solutions of the compounds were prepared at concentrations of 20–50 mM in DMSO and were stored at −80 °C.

### Fluorescence-Based Enzymatic Assays

The enzymatic assays follow the hydrolysis of the fluorogenic dipeptide substrates l-leucine-7-amido-4-coumarin (Leu-AMC) for IRAP/ERAP1 or l-arginine-7-amido-4-coumarin (Arg-AMC) for ERAP2 (Sigma-Aldrich), as previously described,^53^ using the BioTek multimode microplate reader Synergy H1. For the initial screening of the compounds’ inhibitory activity for IRAP, all compounds were tested in two concentrations, 10 and 100 μΜ, in duplicates. The reactions were set up with 50 μΜ fluorogenic substrate and 1 nM recombinant IRAP in 150 mM NaCl, 20 mM HEPES pH 7.0 buffer +0.002% Tween 20. The potency of the most active compounds was assessed by performing titrations in a 0.01 to 30 μΜ range, spaced on a 1/3 log10 scale, while measuring the hydrolysis rate of Leu-AMC. In vitro IC_50_ values were determined by fitting the data to a four-parameter dose–response model using GraphPad Prism 8.0. For the selectivity studies versus ERAP1 and ERAP2, the titrations of the compounds were performed similarly when measuring the hydrolysis of 50 μΜ Leu-AMC by ERAP1 (10 nM) or 50 μΜ Arg-AMC by ERAP2 (8 nM). For the Michaelis–Menten analysis, initial reaction rates were calculated at varying substrate concentrations, ranging from 2.5 to 150 μΜ (2.5, 5, 10, 15, 20, 30, 40, 60, 80, 100, 150, and 200 μΜ), at a fixed enzyme concentration of 1 nM IRAP. The experiment was performed in the absence of the tested compound and in its presence at two or three concentrations. The kinetic parameters *K*_M_ and *k*_cat_ were determined by fitting the data to a classical Michaelis–Menten model in GraphPad Prism.

### HPLC-Based Enzymatic Assay

Vasopressin trimming by IRAP was followed by a high-performance liquid chromatography. Briefly, 20 μM vasopressin was incubated with 2 nM recombinant IRAP in the presence of 100 μM of each of the compounds, at a final volume of 100 μL in 50 mM HEPES buffer (pH 7.5) containing 100 mM NaCl. The reactions were carried out in triplicates, incubated for 1 h at 37 °C, quenched by the addition of 0.5% v/v trifluoroacetic acid (TFA) and kept at −80 °C until analysis. The reactions were analyzed using a reversed-phase C-18 column (Chromolith C-18 column, Merck, Kenilworth, New Jersey, USA) by following the absorbance at 220 nm using a 15 min linear gradient from 10 to 50% solvent B (solvent A: 0.05% TFA, solvent B: 0.05% TFA, 50% acetonitrile). The percentage of substrate cleavage was calculated by integrating the surface of the substrate and product peaks. It is expressed as *% product = [product/(product + remaining substrate)]* and normalized to the corresponding percentage of the product in the case of the control digestion reaction (in the absence of compounds).

Titrations of the most active compounds were also performed to evaluate their potencies for vasopressin trimming. Compound concentrations varied from 0.1 to 100 μΜ (spaced on a 1/3 log10 scale). Two higher concentration points of 150 and 200 μΜ were included to better define the lower plateau. 20 μΜ vasopressin and 3 nM IRAP were used in all reactions carried out in duplicates. The quantification of the reaction’s progression was conducted in the same manner as described above. Data were fitted to a four-parameter [inhibitor] versus response model in GraphPad Prism to determine the IC_50_ values. For the Michaelis–Menten analysis by HPLC, various concentrations of vasopressin (1.25, 2.5, 5, 10, 20, 30, and 40 μΜ) were incubated with 2–4 nM IRAP, in the absence and presence of 100–200 μM of the selected inhibitor. All reactions were performed in duplicates and added up to a final volume of 100 μL in the reaction’s buffer, incubated under the conditions described above, and quenched by the addition of 1:10 v/v TFA 5% solution. The analysis was carried out by fitting the data to a classical Michaelis–Menten model in GraphPad Prism.

## Results

### Targeting the Malate Allosteric Site of IRAP

**Figure 1:**
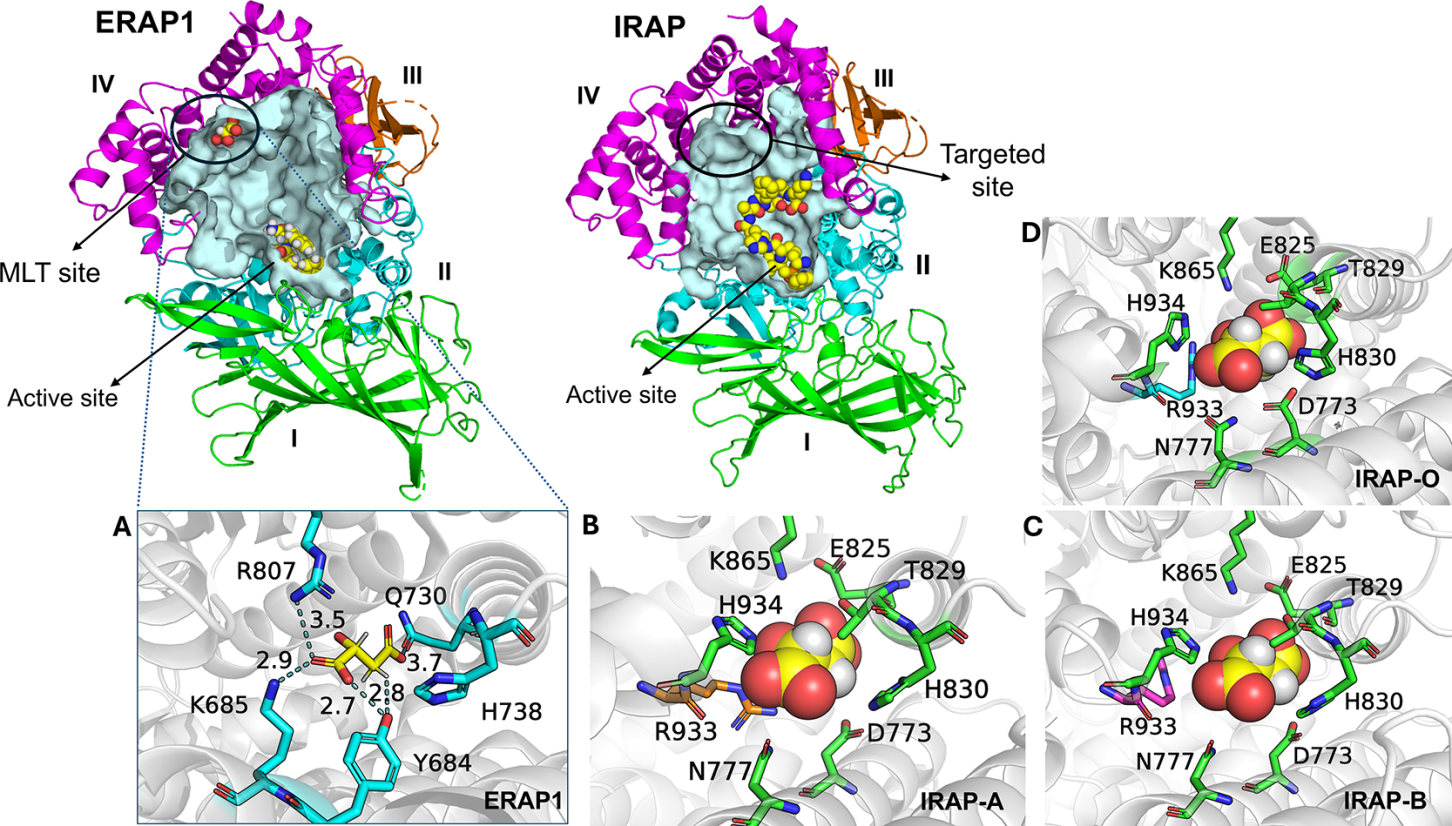
Schematic representations of the internal cavities of ERAP1 (left) and IRAP (right). The figures were generated using the coordinates from PDB IDs 6Q4R and 4Z7I. Internal cavities are depicted as light-cyan surface representations, while proteins are shown in cartoon representations color-coded by domain (I: green, II: cyan, II: orange, IV: magenta). Ligands within each cavity are displayed in sphere representation (C: yellow, N: blue, O: red, H: white). The active sites contain an inhibitor in the case of ERAP1 and a peptide analogue in the case of IRAP. Additionally, the locations of the malate site (MLT) in ERAP1 are indicated alongside the corresponding site in IRAP, which was targeted for virtual ligand screening. (A–D) present in detail the malate-binding site for each enzyme and conformation. For ERAP1 (A), the key amino acid residues that interact with the MLT molecule (C: yellow sticks, O: red, H: white) are shown in cyan, and the interaction distances are presented by dotted lines. For IRAP (B–D), the corresponding MLT cavities were generated upon alignment of ERAP1 structure (PDB: 6Q4R) with IRAP closed (PDB: 5MJ6) and open (PDB: 4Z7I) structures: IRAP-O was generated from 4Z7I, IRAP-A, and IRAP-B from 5MJ6, chains A and B, respectively. Key amino acid residues in proximity to the malate (spheres, C: yellow, O: red, H: white) are depicted with green sticks. The distinct orientations of Arg933 in each IRAP cavity are highlighted by different colors (orange in IRAP-A, magenta in IRAP-B, and cyan in IRAP-O).

Given the importance of the malate (MLT) allosteric site in the homologous enzyme ERAP1 for both biological function and inhibitor development,^27,28^ we targeted the equivalent site in IRAP (Figure 1). In both enzymes, the MLT site is defined as a shallow pocket in domain IV. In ERAP1, the MLT is stabilized by two positively charged amino acids, Lys685 and Arg807, along with Tyr684, through salt bridge formation and hydrogen bonding. These residues play a key role in recognizing the C-terminus of longer peptide substrates by ERAP1.^26^ In IRAP, only one of these three residues is conserved (Tyr776) suggesting a lack of functional conservation of this site (Figure S1). However, inspection of the cavity suggests that other residues such as His934, Arg933, and Asn777 could form interactions with the carboxyl moiety of acidic molecules. Furthermore, Asp773 and Glu825, in the MLT site of IRAP, could favor interactions with molecules carrying positively charged groups. Interestingly, Asp773 is not conserved in ERAP1, which instead holds a hydrophobic residue, Ile668, at the corresponding position. This difference could play a significant role in the selectivity between the two enzymes. In IRAP, the MLT site displays differences between the two known crystallographic conformations of the enzyme,^15,16^ and some differences between the two monomers in the crystallographic dimer are also evident, suggesting some structural plasticity. Specifically, Arg933 displayed two variable side-chain conformers that affect pocket size and the potential for electrostatic interaction with acidic groups (Figure 1B,C). Based on this rationale, we set off to screen for novel IRAP inhibitors targeting the IRAP MLT site.

### Virtual Screening of the Compounds—Free Energy Calculations and Selection of the Compounds for Evaluation

**Figure 2:**
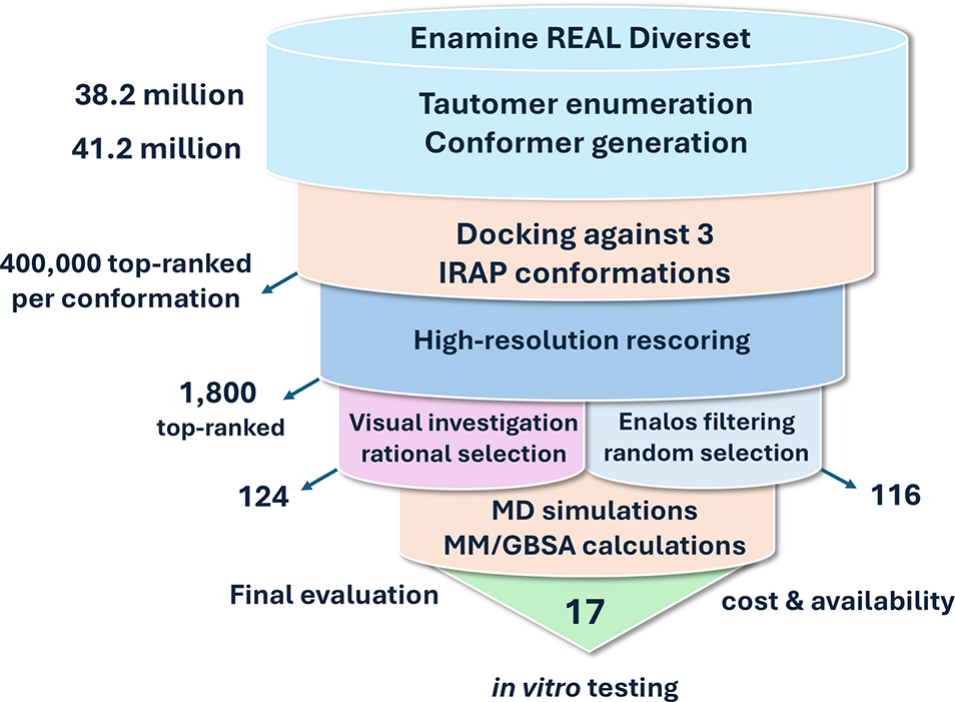
Virtual screening strategy employed for the discovery of lead-like allosteric modulators of IRAP, indicating the filtering method and the number of molecules obtained in each stage.

The screening strategy employed is summarized in Figure 2. To maximize our chances of discovering potent inhibitors of IRAP that bind at the MLT site, we utilized a database of commercially available compounds from ENAMINE. We obtained the ENAMINE REAL diversity set that was composed of approximately 38.2 million compounds (first quarter of 2022) and which represents a 1% subset of the full ENAMINE REAL database but with no similar analogs. The database is also in compliance with Lipinski’s and Veber’s criteria for drug-like compounds. The database was processed with software from OpenEye Inc. for tautomer enumeration, canonicalization, and conformer generation as described in Experimental Methods to yield a 41.2 million diversity set. For docking experiments, we used the fully closed state (PDB ID: 5mj6)^16^ targeting the MLT site of both chains found in the asymmetric unit (IRAP-A and IRAP-B). In addition, we targeted the MLT site of IRAP in an open state (PDB ID: 4z7i),^30^ which displayed side-chain variability of the residues that comprised the pocket with respect to the closed state (IRAP-O). In all, three IRAP sites were used as targets for docking with the ENAMINE database compounds.

Molecular docking was carried out using the OEDocking suite of programs from OpenEye in 1 million compound chunks for each target site. The top-ranked 400,000 compounds (1% of the full database) for each site were rescored with a more refined resolution, and the 1800 top-scored compounds were retained for further analysis. First, we selected a subset of 124 top-ranked compounds based on visual investigation focusing on hydrogen-bonding and salt bridge interactions with the key Arg933 residue, as well as other interactions with the pocket residues Asp773, Asn777, Glu825, Lys865, and His934. In addition, we used the Enalos suite^38^ to filter the 1800 compounds by selecting the ones that scored negative for mutagenicity, inactive for cytotoxicity and having a predicted lipophilicity range of 1.5 < logP < 3.0. Based on this filtering, 398 compounds were found to satisfy these criteria. From these, we randomly selected 116 compounds aiming to broaden the diversity or chemical space explored. These 116 compounds were added to the 124 compounds selected by visual inspection and analysis to yield a 240-compound set that was analyzed further using MD simulations and free energy calculations (Supplementary Tables S1 and S2).

**Chart 1:**
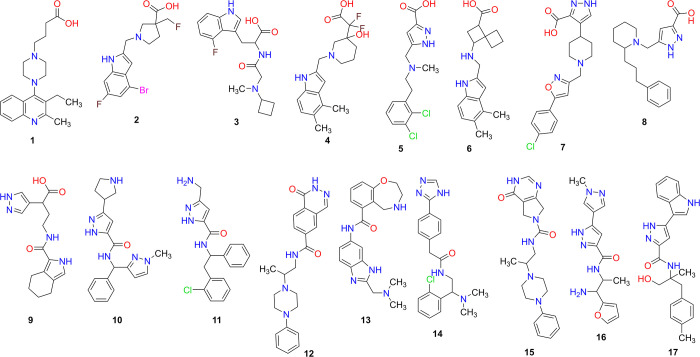
Chemical Structures of the Compounds Selected for Experimental Evaluation as Potential Allosteric Inhibitors of IRAP

To evaluate the stability of the 240 IRAP–ligand complexes, we employed MD simulations in explicit solvent at the nanosecond time scale. Simulations were performed for 20 ns in the isobaric–isothermal ensemble using the AMBER force field for the protein (refer to Experimental Methods for more information). Using 100 snapshots sampled from the simulation (every 0.2 ns), we employed Molecular Mechanics/Generalized Born Surface Area (MM/GBSA) free energy calculations. The results of the simulations were used in conjunction with the MD stability study to prioritize compounds for final selection. We opted to select a representative number of compounds from each site and from both methods of selection. After considering compound availability and price, we ended up with a selection of the 17 compounds shown in Chart 1. Key computed parameters of the selected compounds, including docking scores, predicted binding energy, partition coefficient (logP), water solubility (logS), and the bioconcentration factor (logBCF), are shown in Table 1. Compounds **1**–**9** are carboxylic acids and were selected from the virtual screening using the closed state of IRAP (**1**–**5** from chain A and **6**–**9** from chain B). On the other hand, compounds **10**–**17** that were selected from the open conformation of IRAP are not carboxylic acids, but rather contain amidic groups, some of which comprise amines (**13**, **14**, **16**). Detailed physiochemical properties and pharmacokinetic predictions computed using SwissADME^54^ as well as the LCMS characterization of these compounds are shown in the Supporting Information (Supplemental Figures S2 and S3).

**Table 1:** Calculated Parameters for Selected Compounds, Including Docking (ChemGauss4) Scores, the Estimated Binding Free Energy (Δ*G*_tot_) from MM/GBSA Calculations, and Predicted Pharmacokinetic (PK) Properties from the Enalos Suite Platform: logBCF (Bioconcentration Factor), logP (Lipophilicity), and logS (Solubility)

**compound**	**target conformation**	**docking score**(kcal/mol)	**MM/GBSA Δ***G*_**tot**_(kcal/mol)	**MW**(g/mol)	**MSlogBCF**	**MSlogP**	**MSlogS**
**1**	IRAP-A	–15.27	–116.17	341.45	0.831	2.035	–3.83
**2**	IRAP-A	–16.60	–108.71	373.19	1.977	2.104	–3.754
**3**	IRAP-A	–14.91	–105.78	347.38	1.154	1.804	–3.256
**4**	IRAP-A	–14.70	–127.84	352.38	1.378	0.656	–4.229
**5**	IRAP-A	–16.68	–103.83	328.19	1.23	1.594	–3.937
**6**	IRAP-B	–14.96	–116.11	312.41	1.684	1.983	–4.318
**7**	IRAP-B	–16.03	–110.72	386.83	1.564	1.952	–6.076
**8**	IRAP-B	–14.97	–107.79	327.42	1.428	2.759	–3.389
**9**	IRAP-B	–16.31	–96.38	316.36	0.503	0.037	–1.42
**10**	IRAP-O	–14.70	–127.84	350.42	0.902	1.767	–1.772
**11**	IRAP-O	–15.17	–122.42	354.83	1.835	0.862	–4.575
**12**	IRAP-O	–15.31	–121.16	391.47	1.197	1.901	–5.124
**13**	IRAP-O	–15.97	–120.85	365.43	1.384	2.136	–5.382
**14**	IRAP-O	–14.88	–119.75	383.87	1.561	2.503	–6.02
**15**	IRAP-O	–15.12	–118.01	382.46	0.51	2.442	–4.288
**16**	IRAP-O	–14.87	–115.97	314.34	0.369	–0.55	–1.556
**17**	IRAP-O	–14.71	–114.32	388.46	1.729	1.453	–5.692

### *In Vitro* Screening Using a Small Fluorogenic Peptide

**Figure 3:**
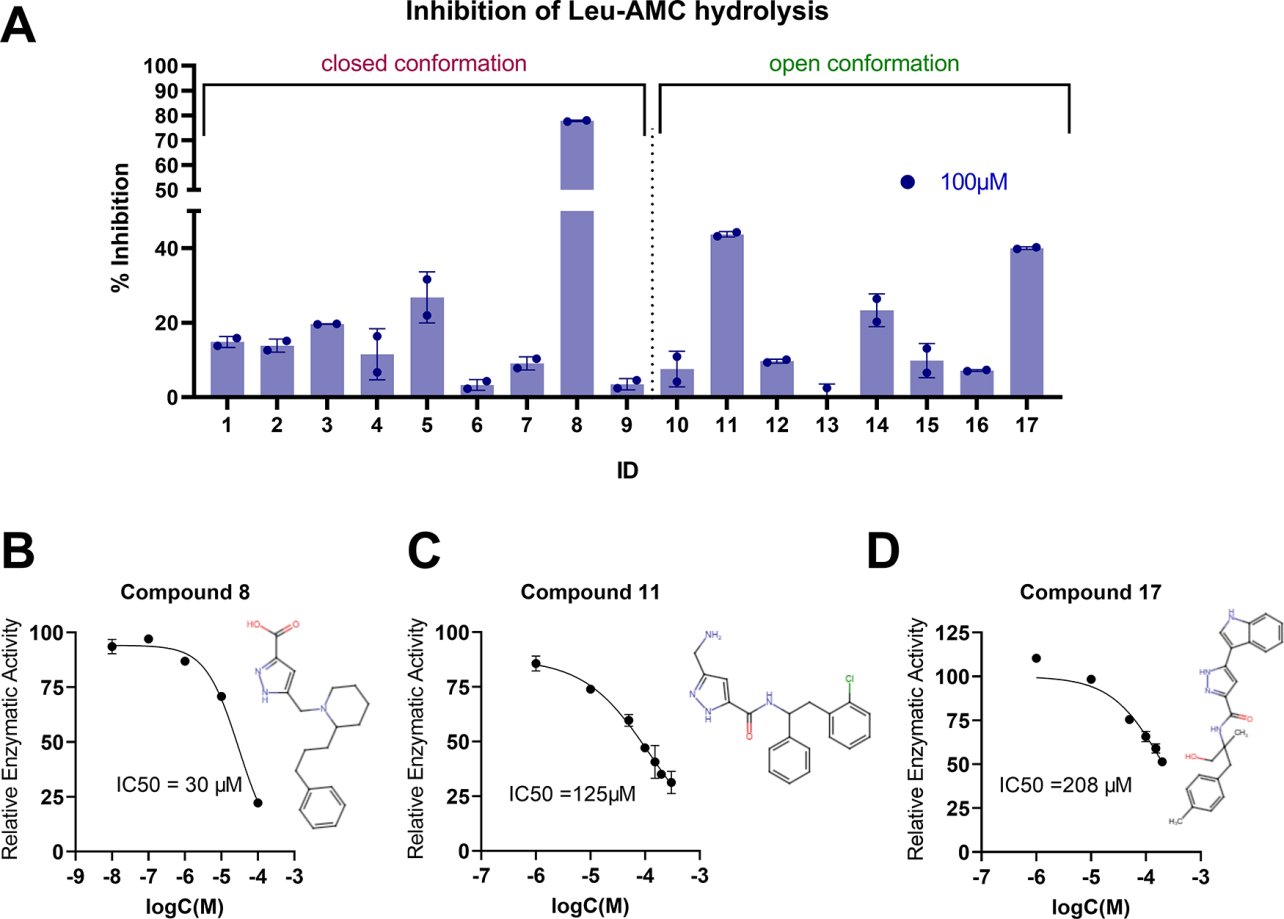
Activity of selected compounds on Leu-AMC hydrolysis by IRAP. (A) % Inhibition of Leu-AMC hydrolysis by IRAP, measured upon the addition of 100 μΜ of the compounds, relative to the digestion control reaction. (B–D) Titrations of the most active compounds for Leu-AMC hydrolysis, normalized to the control reaction. The 2D structure of the corresponding compound is indicated next to each dose–response curve. Error bars represent the standard deviation from duplicate measurements.

The 17 compounds obtained from ENAMINE were evaluated *in vitro* using a fluorescent assay that follows the hydrolysis of the model dipeptide substrate Leu-AMC by IRAP. Initial screening of the compounds was performed at two concentration points (10 and 100 μM). Compounds that reduced enzymatic activity by at least 30% at the highest concentration were considered promising candidates for further investigation (Figure 3A). Among the 17 compounds tested, three were identified as hits: **8**, which originated from the virtual screening in the closed IRAP structure (PDB ID: 5MJ6, chain B), and compounds **11** and **17**, identified from screening in the open structure (PDB ID: 4Z7I). Dose–response curves were generated for these compounds to more accurately determine their potencies toward the inhibition of Leu-AMC hydrolysis, expressed as the half-maximal inhibitory concentration (IC_50_). Compound **8** was the most potent with IC_50_ = 30 μΜ (Figure 3B). Compounds **11** and **17** exhibited lower but still clear inhibition profiles, with IC_50_ values of 125 and 208 μΜ respectively (Figure 3C,D).

### Selectivity of Hit Compounds for IRAP against Homologous Enzymes

**Figure 4:**
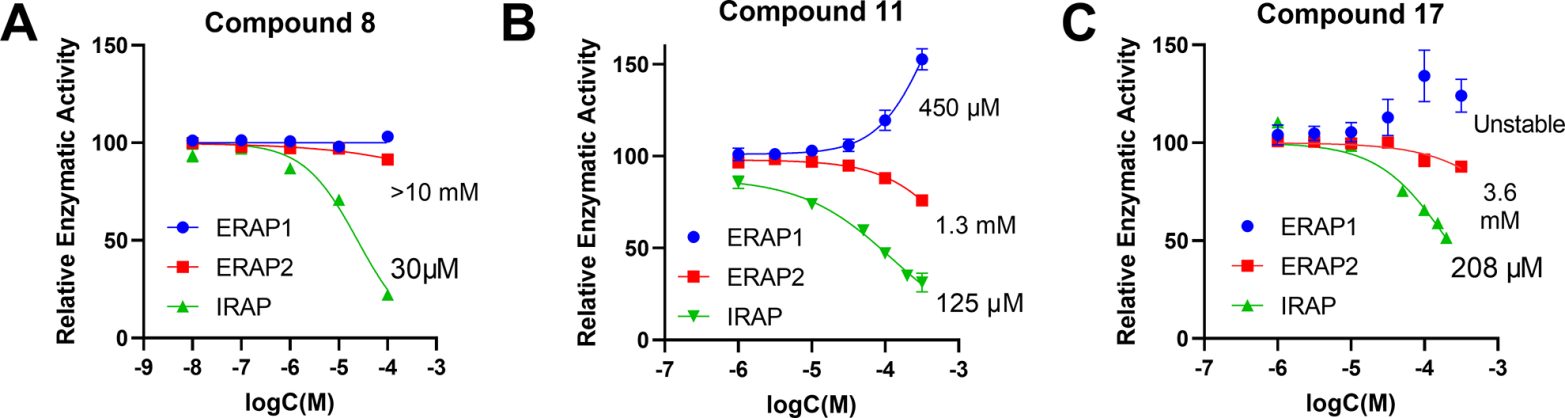
Selectivity against homologous aminopeptidases. Dose–response curves of hit compounds **8**, **11**, and **17** for the hydrolysis of Leu-AMC by IRAP (green) and ERAP1 (blue) and of Arg-AMC by ERAP2 (red), normalized to control reactions, indicating the IC_50_ values for the corresponding enzyme (bottom—IRAP, middle—ERAP2, top—ERAP1). Error bars represent the standard deviation from duplicate measurements.

Since the MLT site has some limited shared structural features in enzymes homologous to IRAP, we tested the selectivity of the discovered hits. Specifically, we evaluated the inhibition profile of the three hits against ERAP1 and ERAP2, enzymes of the M1 family that share ∼50% sequence homology with IRAP, for the hydrolysis of the dipeptidic substrates Leu-AMC and Arg-AMC, respectively. Compounds **8** and **17** were found to be inactive for ERAP1 for concentrations up to 300 μΜ, while compound **11** activated the enzyme, with an estimated EC_50_ value of 450 μΜ. Activation of ERAP1 by the MLT site has been described before and is a property unique to that enzyme.^28^ Compound **8** exhibited a high selectivity over ERAP2 by approximately 300-fold (Figure 4A). **11** and **17** also demonstrated preferential potencies toward IRAP by 10- and 17-fold, respectively (Figure 4B,C).

### Inhibition of Vasopressin Trimming

**Figure 5:**
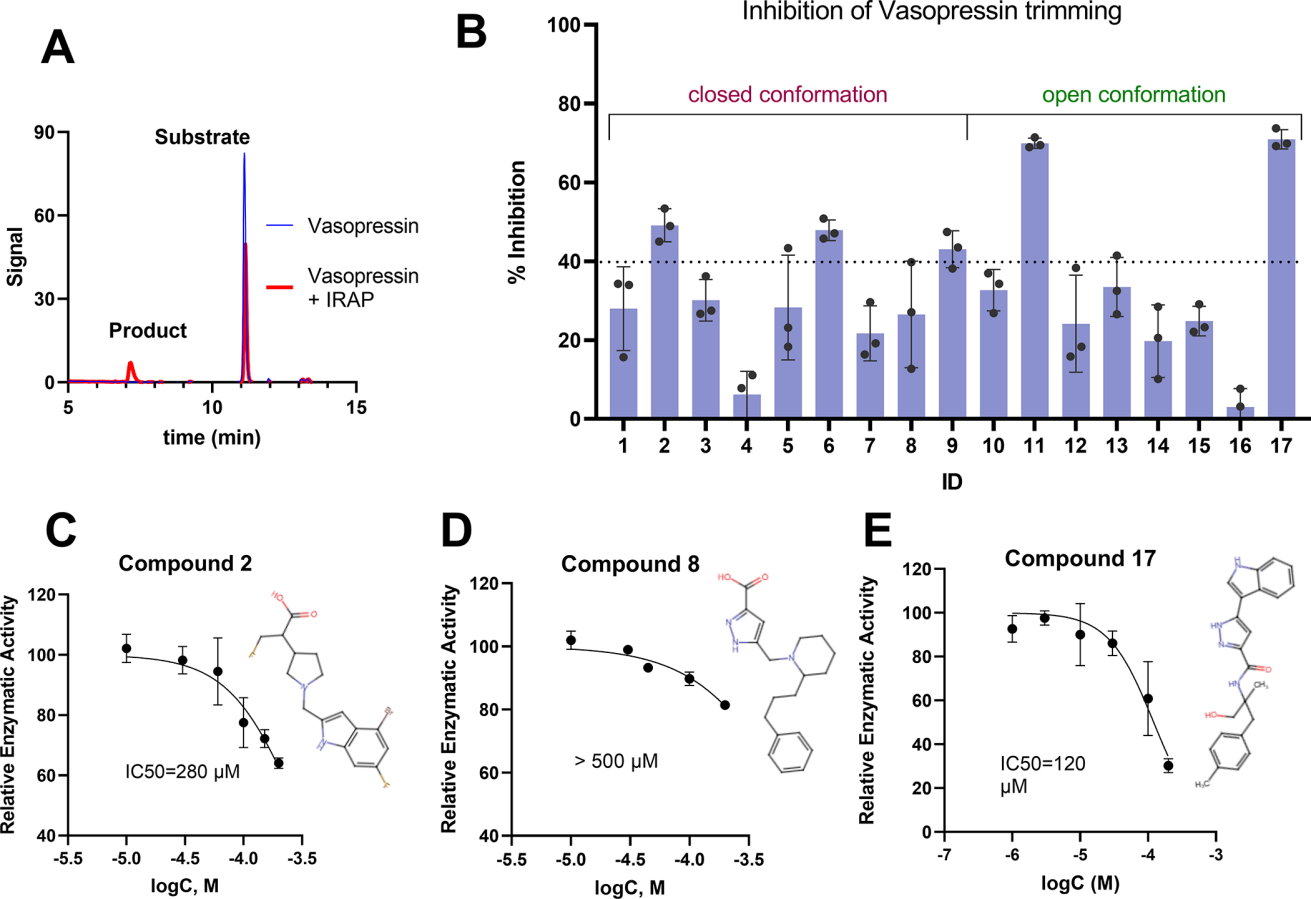
Activity of selected compounds on vasopressin trimming by IRAP. (A) HPLC trace, illustrating the peak of the peptide before the enzymatic reaction (blue line) and the resulting peaks corresponding to the product and the remaining substrate after IRAP-mediated digestion (red line). (B) % Inhibition of vasopressin trimming by IRAP, measured upon the addition of 100 μΜ of each compound, relative to the digestion control reaction. (C–E) Titrations of the most active compounds for the trimming of vasopressin, normalized to control reaction. 2D structures of the corresponding compound are indicated next to each dose–response curve. Error bars represent the standard deviation from triplicate (B) and duplicate (C–E) measurements.

We have previously demonstrated that IRAP may use different mechanisms to trim peptidic substrates of different lengths and sizes, and this can affect the potency of small-molecule inhibitors that target either the active or allosteric sites.^17,21^ Thus, we evaluated the inhibitory activity of the 17 selected compounds toward the trimming of the cyclic peptide hormone vasopressin, a physiological substrate of IRAP. The cleavage of vasopressin was monitored by HPLC, in a manner similar to the trimming of oxytocin previously reported^55^ (Figure 5A). The ability of the compounds to inhibit vasopressin trimming was initially evaluated at 100 μΜ (Figure 5B). **11** and **17**, discovered from screening the open conformation of IRAP, were the most active, resulting in ∼70% inhibition of the enzymatic reaction. From compounds discovered by screening the closed conformation (chain A or B), two showed ∼50% inhibition, **2** (chain A) and **6** (chain B). Interestingly, those compounds were inactive in the Leu-AMC assay, while **8**, the most active Leu-AMC inhibitor, exhibited only moderate activity with vasopressin. Dose–response curves were performed to determine the IC_50_ values. Compound **2** demonstrated a potency of 280 μΜ (Figure 5C), whereas **8** displayed a very weak potency, with an estimated IC_50_ >500 μΜ (Figure 5D). For compounds discovered by screening in the open conformation of IRAP, **17** was chosen for further characterization over compound **11** due to its more favorable selectivity profile for IRAP (Figure 4B,C). This compound emerged as the best hit for inhibiting vasopressin trimming, exhibiting an IC_50_ value of 120 μΜ (Figure 5E).

### Mechanism of Inhibition

**Figure 6:**
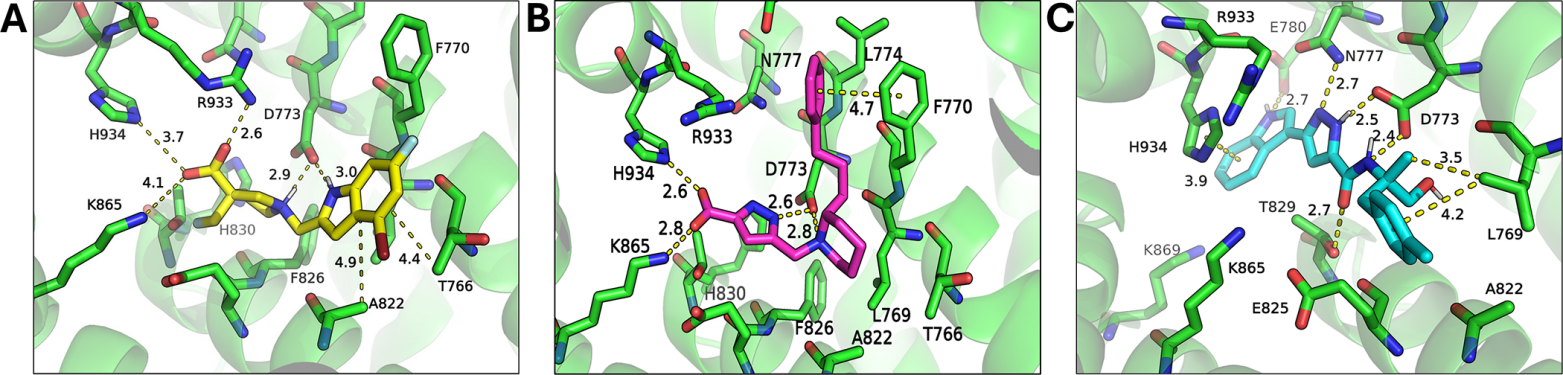
Key interactions of hit compounds in the corresponding MLT docking site. (A) Compound **2** (yellow sticks) docked in IRAP-A cavity. (B) Compound **8** (magenta sticks) docked in IRAP-B cavity. (C) Compound **17** (cyan sticks) docked in IRAP-O cavity. Interactions with the key residues of the MLT site (green sticks) are indicated with dashed lines and the corresponding distances in Å. Panels show only one of the two possible enantiomers tested in the racemic mixture provided by the supplier.

The inhibition profiles of the selected compounds varied significantly depending on the substrate used, something reminiscent of the complex effects of the commonly used IRAP inhibitor HFI-419, which is potent for Leu-AMC but very weak for a cyclic peptidic substrate.^17^ This observation prompted us to investigate the mechanism of inhibition of the most active compounds since they originate from virtual screening strategies targeting distinct conformations. We performed Michaelis–Menten kinetic analysis using both substrates, the small Leu-AMC, and the larger cyclic peptide substrate, vasopressin. Two representative compounds were studied for each case: one discovered from screening the open conformation (IRAP-O) and one from the closed conformation (IRAP-A or IRAP-B). Compounds **2**, **8**, and **17** that exhibited significant inhibitory activities for one or both corresponding substrates were chosen. The potential interactions of each compound in the binding site are shown in Figure 6. Compound **2**, selected to target monomer A of the closed IRAP conformation, is predicted to engage its carboxyl group in ionic interactions with three basic amino acids, namely, Lys865, His934, and Arg933. The heterocyclic amine can interact with Asp773 via hydrogen bonding, and the aromatic moiety can be stabilized by hydrophobic interactions with Ala822 and Thr766 (Figure 6A). Compound **8**, selected to target monomer B of the closed IRAP conformation is predicted to bind with its carboxyl group via hydrogen bonding interactions with Lys865 and His934, two interactions that are common with **2** in chain A. Similar to compound **2**, Asp733 potentially interacts with the heterocycle moiety of the compound and the phenyl group forms a T-shaped π–π stacking interaction with Phe770 (Figure 6B). Compound **17** that is not acid and was selected from the open conformation of IRAP is predicted to form hydrogen bonding interactions with Glu780, Asn777, Thr829, and Asp733, π–π stacking interactions with His934, and hydrophobic interactions with Leu769 (Figure 6C), the latter being common with both **2** and **8** as well.

**Figure 7:**
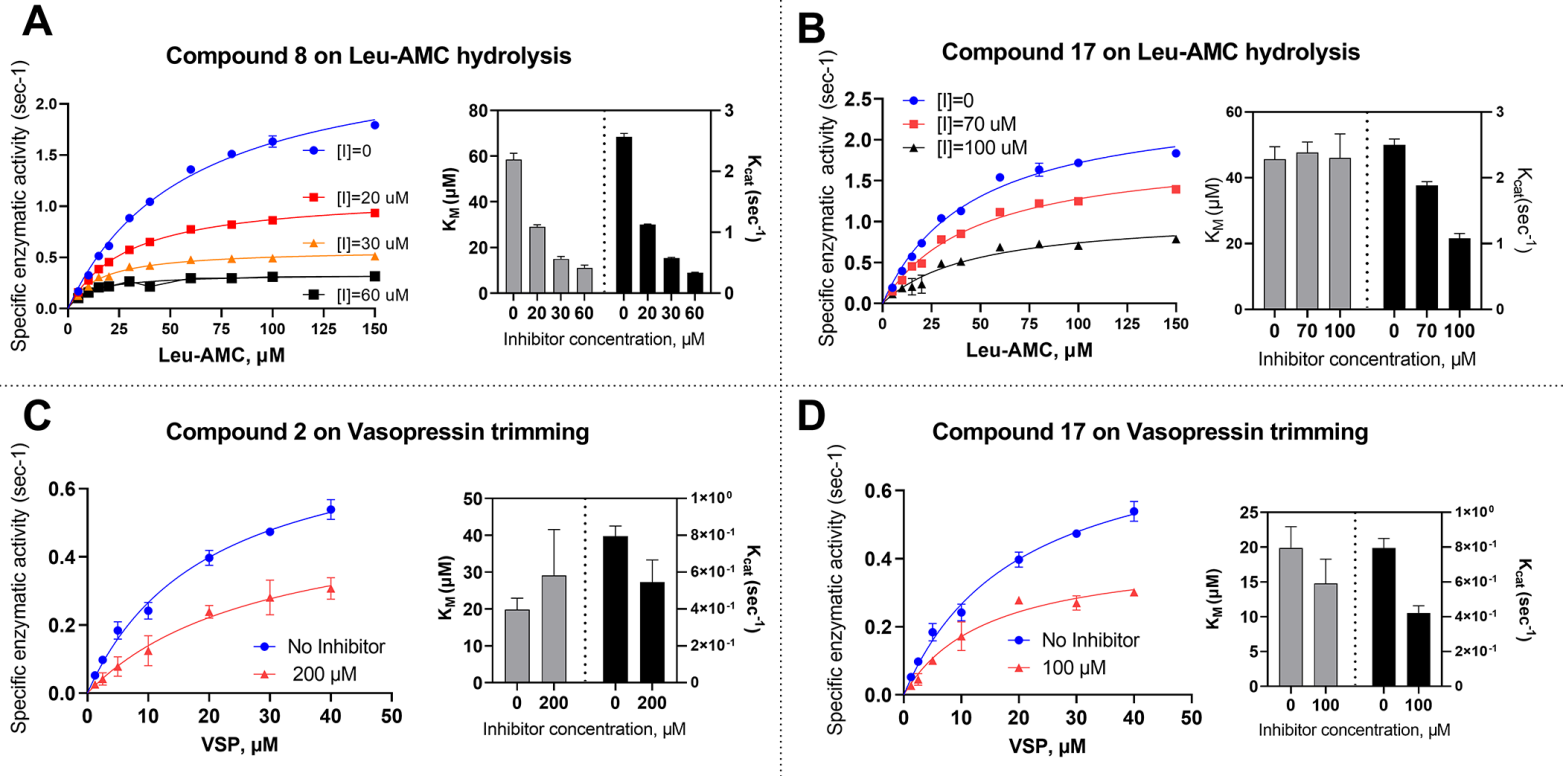
Michaelis–Menten analysis of (A, B) Leu-AMC substrate digestion by IRAP in the presence of increasing concentrations of compounds **8** (A) and **17** (B). (C, D) Vasopressin trimming in the presence or absence of compounds **2** (C) and **17** (D). In each panel, the Michaelis–Menten fits are shown on the left, while the calculated parameters, *k*_cat_ and *K*_M_, are presented on the right. For the Michaelis–Menten fits, error bars are calculated from triplicate measurements. For the bar graphs, error bars correspond to the standard deviation calculated from the 95% confidence interval of the corresponding fit.

For the small substrate, compounds **8** (IRAP-B), the most active hit, and **17** (IRAP-O) were further characterized. The enzymatic reaction was carried out in the presence of increasing concentrations of the inhibitors to follow the variation of kinetic parameters *K*_Μ_ (Μ) and *k*_cat_ (sec^–1^) and determine the mode of inhibition. Compound **8**, the best hit for the Leu-AMC substrate, was found to be an uncompetitive inhibitor as both kinetic parameters decreased for increasing compound concentrations (Figure 7A). Compound **17** exhibited a noncompetitive mechanism as *k*_cat_ was reduced by the inhibitor, while *K*_Μ_ remained constant (Figure 7B).

Since compound **17** was also effective in inhibiting vasopressin trimming, we first characterized its mechanism of action versus that of this large substrate (Figure 7C). Compound **17** was found to be a noncompetitive inhibitor for vasopressin, similar to its mechanism of action for Leu-AMC. The second compound evaluated for the cleavage of vasopressin was **2**, which was discovered by screening the closed conformation of IRAP and was found to be active for this substrate but not for Leu-AMC. In the presence of 200 μΜ of **2**, *k*_cat_ decreased and *K*_Μ_ (Μ) showed a small but not statistically significant increase, suggesting a noncompetitive mechanism of inhibition (Figure 7D).

Overall, all characterized hits displayed noncompetitive inhibition kinetics, irrespective of target conformation and substrate, which is consistent with their expected allosteric nature. However, the discovery of uncompetitive mechanisms of action for compound **8**, suggests that the MLT site can, in some cases, affect the active site, a phenomenon reminiscent of the regulatory properties of this site in the homologous ERAP1.^26^

## Discussion

IRAP belongs to the family of M1 aminopeptidases, which contains more than 60 members according to the MEROPS database.^56^ All members utilize a similar mechanism of action that relies on a single zinc(II) atom in the catalytic site and a series of conserved residues for N-terminus recognition and catalysis.^57^ As a result, inhibitors targeting the active site run a high risk of also targeting other members of the family, limiting their clinical potential. A potential solution to this limitation is to instead target allosteric sites that tend to be significantly less conserved and thus enhance the chances for higher selectivity with the trade-off of more complex mechanisms of action. Indeed, we have recently discovered that two widely utilized IRAP inhibitors, although thought to occupy the active site, are actually allosteric, with variable repercussions on their ability to inhibit the processing of some substrates.^17,21^ We previously explored another cavity (B3P site) of IRAP and discovered inhibitor hits that however had complex substrate dependency.^55^ Here, we explored the MLT site in IRAP, which is a functionally relevant site in homologous ERAP1 and has been successfully utilized to generate highly potent and selective inhibitors for that enzyme. This site in ERAP1 has known overlap with peptidic substrates and thus yielded competitive inhibitors.^27,28^ In contrast, no known overlap between IRAP substrates and the IRAP MLT site exists, raising doubts if this site can be utilized to inhibit IRAP activity. However, IRAP has been suggested to cycle between different conformations for its catalytic cycle, and the MLT site undergoes some structural changes between conformations, suggesting that the inhibitor binding there may interfere with the catalytic cycle.

Our results suggest that the MLT site is a tractable target site for generating novel IRAP inhibitor hits. By screening a 38-million drug-like compound database and then filtering results by a combination of rational and computational tools, we identified several potential hits. These hits cover the full landscape of conformations targeted and substrate types, constituting a valuable starting arsenal of hits to modulate different facets of IRAP function. Kinetic analysis supports an allosteric mechanism as per design since the MLT is not known to be utilized by any IRAP substrates, long or short, based on crystallographic analyses. The mechanism of action of these inhibitor hits needs to be further validated but likely lies in blocking the conformational rearrangements of IRAP necessary for its catalytic cycle.^16,17,58^ Stabilization of the MLT site by compound binding may affect IRAP’s ability to cycle between open and closed conformations, limiting the apparent catalytic turnover in a substrate-independent fashion. A similar mechanism has been proposed for allosteric sites in ERAP1, where small-angle X-ray scattering measurements suggested a transition to the closed conformation upon ligand binding.^59^ Further experiments will be necessary to test whether some or all of the hits described in this study utilize a similar mechanism of action.

More careful inspection of the experimental kinetic characterization of the three major hits provides further insight into the mechanisms employed by IRAP for different substrates. Compound **2** appears to inhibit only vasopressin trimming and not Leu-AMC trimming and utilizes a noncompetitive mechanism. Compound **2** was discovered as a hit, targeting the closed conformation of IRAP, which has been proposed to be necessary for Leu-AMC trimming. Thus, by shifting the conformational equilibrium to the closed conformation, compound **2** can be understood to block vasopressin binding while still allowing for Leu-AMC binding and trimming. Conversely, compound **17** was designed to bind to the open conformation and inhibit both substrates. This behavior can be rationalized by the ability of the inhibitor to block conformational transitions and at the same time stabilize the conformation that is inefficient for small substrate cleavage, as suggested before.^17,52^ Finally, compound **8**, although designed for the closed conformation, can block the processing of only the small substrate and utilize an uncompetitive mechanism of action. This is highly reminiscent of inhibitor HFI-419, which was recently demonstrated to be allosteric, although it utilizes a different site.^17^

Our results suggest that it may be possible to generate IRAP inhibitors that target different classes of IRAP substrates, small and large. Inhibitors that target the open conformation through the MLT site can inhibit both classes, whereas targeting the closed conformation can result in selective substrate blocking depending on the compound. Indeed, in a recent study, we proposed that an additional “wide-open” conformation, to the two analyzed by X-ray crystallography, may exist for IRAP.^21^ If this is true, then it would explain why targeting the open conformation may affect both classes of substrates: all substrates have to cycle through the open conformation for their catalytic cycle.^21^

Overall, we describe novel IRAP inhibitor hits discovered through computational screening and characterized by kinetic analyses. These hits suggest that the MLT site is a tractable site for generating novel IRAP inhibitors with potential substrate selectivity. A limitation of this study is that the discovered compounds present weak binding, and substantial effort in medicinal chemistry campaigns will be necessary for their optimization. Weak affinities may also bias kinetic experiments, thus limiting our confidence in the molecular mechanisms proposed. Still, our results are consistent with the emerging hypothesis that IRAP utilizes different conformations for different substrates and establishes a path forward to generating substrate-selective inhibitor leads targeting specific IRAP biological functions.

## Data Availability

All data for this study is available throughout the manuscript and supporting files.

## Supporting Information Available

Superposition of ERAP1 and IRAP MLT sites, results of MM/GBSA calculations for the initial selection of 240 compounds, Enalos Suite predictions for the 240 compounds, and detailed physiochemical properties, pharmacokinetic predictions, and LCMS characterization for the 17 purchased compounds (PDF)
