# Supplementary material for: Discovery of Novel Allosteric Inhibitor Hits for Insulin-Regulated Aminopeptidase Provides Insights on Enzymatic Mechanism
Source: ACS Omega. 2025 Apr 23;10(17):17960–72. doi: 10.1021/acsomega.5c01169 (PMC12059914; doi:10.1021/acsomega.5c01169)

Supplementary Material

Discovery of novel allosteric inhibitor hits for Insulin-Regulated Aminopeptidase provides insights on enzymatic mechanism

Galateia Georgaki[1,2], Nikoletta-Maria Koutroumpa[3,4], Panagiotis Lagarias[3], Antreas Afantitis[3], Athanasios Papakyriakou[2,*], Efstratios Stratikos[1,2,*]

[1]Laboratory of Biochemistry, Department of Chemistry, National and Kapodistrian University of Athens, 15784 Zografou, Greece
[2]National Centre for Scientific Research Demokritos, 15341 Agia Paraskevi, Greece
[3]NovaMechanics Ltd., Nicosia 1070, Cyprus
[4] School of Chemical Engineering, National Technical University of Athens, Athens, 15780, Greece
[*]Correspondence: Athanasios Papakyriakou, thpap@bio.demokritos.gr | Efstratios Stratikos, estratikos@chem.uoa.gr

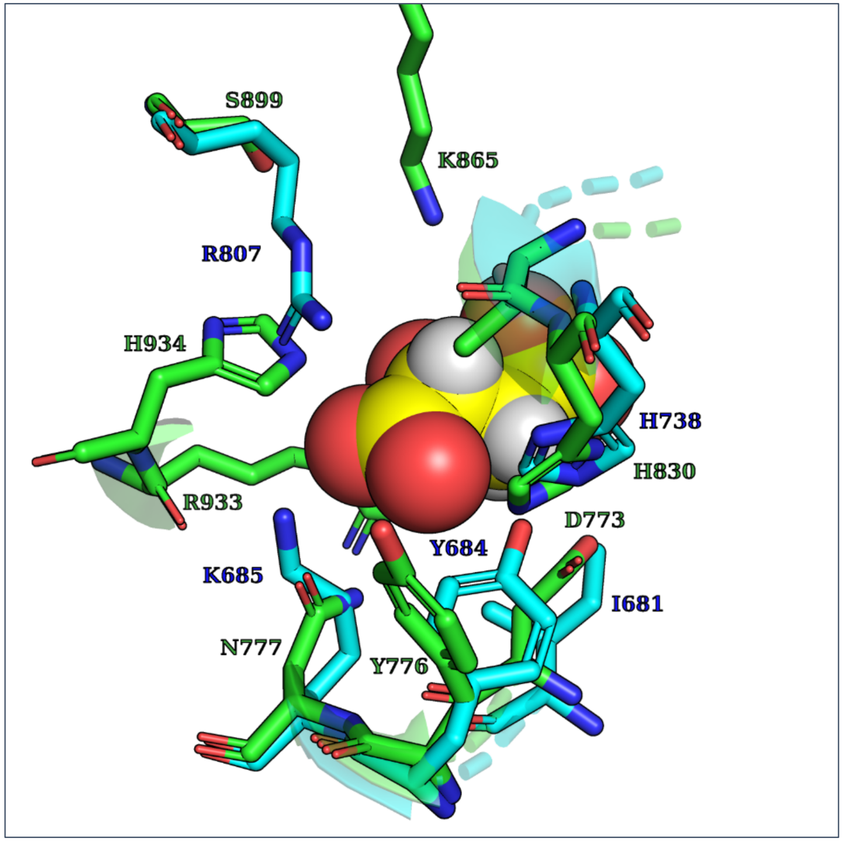

Supplemental Figure S1: Key residues in ERAP1 and IRAP "malate" sites, superposed. Image is generated upon structural alignment of PDB structures 6Q4R for ERAP1 and 5MJ6 (chain A) for IRAP. ERAP1 residues are shown in cyan and their respective labels in blue. The corresponding IRAP residues and labels are shown in green. The malate is depicted with spheres (C: yellow, O: red, H: white).

Table S1. The initial selection of 240 compounds that were evaluated using MM/GBSA calculations showing the initial selection method, the docking (ChemGauss4) score and the estimated binding free energy in both in kcal/mol. The compounds that were obtained for experimental validation are highlighted and indicated by the ID numbers **1–17**.

ID	ENAMINE ID	CXSMILES	IRAP state	Selection	Docking score	MM/GBSA score
	**Z3861670572**	**O=C(O)C(F)(F)C1(O)CCCN(CC2=CC3=CC(Cl)=C(Cl)C=C3N2)C1**	IRAP-A	VISUAL	-16.76	-121.67
	**Z4250350143**	**CC1=NC(CCN(C)CC2=CC(C(=O)O)=NN2)=CC=C1**	IRAP-A	VISUAL	-15.43	-116.30
**1**	**Z5341541922**	**CCC1=C(C)N=C2C=CC=CC2=C1N1CCN(CCCC(=O)O)CC1**	IRAP-A	RANDOM	-15.27	-116.17
	**Z3838877942**	**O=C(O)[C@@H]1CC[C@@](F)(CNCC2=CC3=C(Br)C=CC=C3N2)C1**	IRAP-A	VISUAL	-15.73	-114.75
	**Z5422924976**	**CC1=C(C)C=C2C(N(C)C(CCN3CCOCC3)C(=O)O)=NC=NC2=C1**	IRAP-A	RANDOM	-15.62	-114.64
	**Z5341541542**	**COC1=CC=C2N=C3CCCCC3=C(N3CCN(CCCC(=O)O)CC3)C2=C1**	IRAP-A	RANDOM	-15.54	-112.85
	**Z4210355805**	**CC(C)(C)N1C=C(CNCC2=NNC(C(=O)O)=C2)C(C2=CC=CC=C2Cl)=N1**	IRAP-A	VISUAL	-14.89	-111.59
	**Z4212262952**	**O=C(O)C(F)(F)C1(O)CCN(CC2=CC3=CC=C(Br)C=C3N2)C1**	IRAP-A	VISUAL	-17.34	-111.43
	**PV-004727649566**	**C[C@H](O)[C@@H](C(=O)N1CC[C@@H](O)[C@H](NC(=O)C2=CC(Cl)=CN2)C1)N(C)C**	IRAP-A	VISUAL	-16.01	-111.19
	**Z4562168048**	**CC(NC1C2CCC1CC1=CC=CC=C1C2)C1=NC(C(=O)O)=NN1**	IRAP-A	RANDOM	-16.19	-110.74
	**PV-003401519556**	**CC1=CC=CN=C1OC1CCN(C(C)C2=NC(C(=O)O)=NN2)CC1**	IRAP-A	VISUAL	-15.60	-109.82
	**Z4250065604**	**O=C(O)C(F)(F)C1(O)CCN(CC2=NC3=C(F)C=CC=C3N2)C1**	IRAP-A	VISUAL	-16.13	-108.84
**2**	**Z5179432492**	**O=C(O)C1(CF)CCN(CC2=CC3=C(Br)C=C(F)C=C3N2)C1**	IRAP-A	RANDOM	-16.60	-108.71
	**Z5269220534**	**CC1=CC2=C(C=C1Cl)C(NC1CCN(CCCC(=O)O)CC1)CO2**	IRAP-A	RANDOM	-15.51	-107.44
	**Z4387367234**	**COC1=CC=CC(F)=C1N1CCN(CC2=NC(C(=O)O)=NN2)CC1**	IRAP-A	VISUAL	-16.02	-106.93
	**Z4247724324**	**COC1=CC=C2NC(C(=O)NCCC(F)(F)C(=O)O)=CC2=C1**	IRAP-A	VISUAL	-16.88	-105.95
	**Z4210353111**	**O=C(O)C1=CC(CNCC2=CC=CC=C2C2CCC2)=NN1**	IRAP-A	RANDOM	-15.64	-105.78
**3**	**Z3390553169**	**CN(CC(=O)NC(CC1=CNC2=CC=CC(F)=C12)C(=O)O)C1CCC1**	IRAP-A	RANDOM	-14.91	-105.78
	**PV-003310368609**	**CN(CC1=CC(C(=O)O)=NN1)CC1=NNC2=C1COCC2**	IRAP-A	VISUAL	-18.19	-104.81
**4**	**Z3861671484**	**CC1=CC=C2NC(CN3CCCC(O)(C(F)(F)C(=O)O)C3)=CC2=C1C**	IRAP-A	VISUAL	-17.04	-104.64

ID	ENAMINE ID	CXSMILES	IRAP state	Selection	Docking score	MM/GBSA score
	Z4387366308	CN1C=C(C2CCN(CC3=NC(C(=O)O)=NN3)C2)C2=CC=CC=C21	IRAP-A	VISUAL	-16.18	-103.90
	Z4250158149	O=C(O)C1COC(CN(CC2=CN(C3=CC=CC=C3)N=C2)C2CC2)=N1	IRAP-A	RANDOM	-16.73	-103.90
	PV-004622855944	O=C(O)C[C@@H]1[C@@H](O)C[C@H]1NC(=O)C1(CC2=CC=C(Br)C=C2)CC1	IRAP-A	RANDOM	-14.92	-103.86
5	Z4250351400	CN(CCC1=CC=CC(Cl)=C1Cl)CC1=CC(C(=O)O)=NN1	IRAP-A	RANDOM	-16.68	-103.83
	Z4381214573	O=C(O)C(F)C1CN(CC2=NNC(C3=CC=CC=C3)=C2)C1	IRAP-A	VISUAL	-16.00	-103.76
	Z2898202771	CN(CC1=CC(C(=O)O)=NN1)C1CCC2=CC=CC=C2C1	IRAP-A	RANDOM	-15.55	-103.73
	PV-002804155214	CC1=CN2C(CNCC3=NNC(C(=O)O)=C3)=CN=C2C=C1	IRAP-A	VISUAL	-16.52	-103.61
	Z4387334824	CC(C)C1=CC=C2CN(CC3=NC(C(=O)O)=NN3)CCC2=C1	IRAP-A	VISUAL	-15.73	-103.51
	Z4251430825	O=C(O)C1=NC=C(CN2CCC(C3=NC=C4CCCCN43)CC2)S1	IRAP-A	RANDOM	-15.64	-103.30
	PV-002967423450	O=C(O)C1=NNC(CN2CCN(C3=CC=CC4=C3C=NN4)CC2)=N1	IRAP-A	VISUAL	-16.33	-103.20
	Z4387360883	CC1=C(C2=CCN(CC3=NC(C(=O)O)=NN3)CC2)C2=CC=CC=C2N1	IRAP-A	VISUAL	-16.99	-102.85
	PV-004713760776	CC(CCNC(=O)C1CCCC2=C1NN=C2)N(C)C(=O)C1=CC=CC2=C1NN=N2	IRAP-A	RANDOM	-15.67	-102.82
	PV-007066194544	CN(CCNC(=O)C1CCC2=C1NN=C2)C(=O)C1CC12CC(OC(C)(C)C)C2	IRAP-A	RANDOM	-15.26	-102.42
	Z4001682850	COC1=CC=CC=C1C1=CCN(CC2=CC(C(=O)O)=NN2)CC1	IRAP-A	RANDOM	-15.27	-102.41
	Z4250353294	CN(CCC1COC(C)(C)C1)CC1=CC(C(=O)O)=NN1	IRAP-A	VISUAL	-15.68	-102.36
	PV-002804155214	CC1=CN2C(CNCC3=NNC(C(=O)O)=C3)=CN=C2C=C1	IRAP-A	VISUAL	-14.89	-101.87
	Z3777363876	O=C(O)CCCN1CCC(NCC2(C3CCC3)CC2)CC1	IRAP-A	RANDOM	-15.27	-101.74
	PV-003310368609	CN(CC1=CC(C(=O)O)=NN1)CC1=NNC2=C1COCC2	IRAP-A	VISUAL	-16.68	-101.53
	Z4210355917	CC(C)N1C(CNCC2=NNC(C(=O)O)=C2)=NC2=CC=CC=C21	IRAP-A	VISUAL	-15.33	-101.23
	Z2911606713	COC1=CC=C(C(=O)O)C2=C1CN(C(=O)C1CC(C)CC3=C1NN=C3)CC2	IRAP-A	RANDOM	-15.23	-101.17
	Z4251268391	CC1=C(Cl)C=CC=C1N1CCCN(CC2=CC(C(=O)O)=NN2)CC1	IRAP-A	VISUAL	-15.34	-100.78
	Z2898236840	CC(CC1=CC=CC=C1C(F)(F)F)N(C)CC1=CC(C(=O)O)=NN1	IRAP-A	RANDOM	-16.54	-100.69
	Z4341677602	O=C(O)CN1CC(NCC2=CC3=CC=C(F)C=C3N2)C1	IRAP-A	VISUAL	-16.65	-100.51
	PV-002781370939	COC(=O)C1=CNC(CN2CC(O)(C(F)C(=O)O)C2)=C1C1CC1	IRAP-A	VISUAL	-15.70	-100.17
	Z4562167868	CC(N[C@H]1CCC2=C1C=CC=C2F)C1=NC(C(=O)O)=NN1	IRAP-A	VISUAL	-15.99	-99.81

ID	ENAMINE ID	CXSMILES	IRAP state	Selection	Docking score	MM/GBSA score
	PV-006852601960	CCC1=C(C(=O)O)SC(NCC2=NC(C3=CC=CS3)=CN2)=N1	IRAP-A	VISUAL	-16.70	-99.79
	Z3655568854	CN(CCC1=CC=CC=C1)CC(=O)NC(CC1=COC2=CC=CC=C12)C(=O)O	IRAP-A	RANDOM	-15.23	-99.66
	Z4210355082	O=C(O)C1=CC(CNCC2=CC=C3OCCCCOC3=C2)=NN1	IRAP-A	RANDOM	-15.48	-99.47
	Z5182662261	CC1=CC(C)=C(C=CCN(C)CC2=NN=C(C(=O)O)S2)C(C)=C1	IRAP-A	VISUAL	-14.90	-99.29
	Z5434197110	O=C(O)C1=NNC(CN2CCC(C3=CNC4=CC=CC=C34)C2)=C1	IRAP-A	VISUAL	-15.68	-99.27
	PV-003730731431	CCC(C1=NN=CN1)N(C)CC1=CC(C(=O)O)=NN1	IRAP-A	VISUAL	-16.10	-99.11
	Z4168669561	CC1=C2CCCC2=CC=C1CN1CCC(C2=CNN=C2C(=O)O)CC1	IRAP-A	RANDOM	-15.77	-99.08
	Z4388146723	CC1=C(CN2C[C@@H](O)[C@H](CC(=O)O)C2)SC2=CC=CC=C12	IRAP-A	RANDOM	-15.25	-98.93
	Z3347815877	CCC1=CC=CC2=C1CCN(CC1=CC(C(=O)O)=NN1)C2	IRAP-A	RANDOM	-15.64	-98.79
	Z4250352285	CN(CC(=O)N1CCCCC1)CC1=CC(C(=O)O)=NN1	IRAP-A	VISUAL	-15.66	-98.65
	PV-003948874842	O=C(O)C1=NNC(CN2CCC3=CC=C(OCC4=CC=CC=C4)C=C3C2)=C1	IRAP-A	RANDOM	-15.66	-98.23
	PV-002981194334	CC1=CC=C(C2=CC=CC=C2)C=C1C(=O)NCC1=NNC(C(=O)O)=C1	IRAP-A	RANDOM	-16.85	-98.07
	PV-007098233941	CC1=CC(Br)=C2OCCN(CC3=NC(C(=O)O)=NN3)CC2=C1	IRAP-A	VISUAL	-15.75	-98.01
	PV-005890634210	CN(CCNC(=O)C1=C2CCCCC2=CN1)C(=O)[C@@H]1[C@@H](C2=CC=NC=C2)C1(C)C	IRAP-A	RANDOM	-15.23	-97.55
	Z4387360928	CCC(NCC1=NC(C(=O)O)=NN1)C1=CC=CC(Cl)=C1	IRAP-A	VISUAL	-14.90	-97.54
	PV-005654514600	CCC1=CC(C(=O)N2CCN(C(=O)C3=NNC4=C3C=CC=C4C)CC2)=NC=N1	IRAP-A	RANDOM	-17.26	-97.31
	Z4562164875	CC(C1=NC(C(=O)O)=NN1)N(C)C1CCC2=CC(Br)=CC=C2C1	IRAP-A	RANDOM	-16.11	-97.11
	Z3347937904	CC1(C)CCC2(CCC(CNC3CCN(CCCC(=O)O)CC3)O2)CC1	IRAP-A	RANDOM	-15.63	-97.00
	Z4210354573	O=C(O)C1=CC(CNCC2=C(C3=CC=CC=C3)N(C3CCCC3)N=C2)=NN1	IRAP-A	RANDOM	-16.06	-97.00
	PV-002969507796	CC1=CC(C(=O)O)=NC=C1CN(C)CC1=CC2=CC=CC=C2N1	IRAP-A	VISUAL	-15.71	-96.95
	Z4000894115	O=C(O)C1=COC(CN2CCC(C3CC4=CC=CC=C4C3)C2)=N1	IRAP-A	RANDOM	-15.58	-96.77
	Z3635314177	CN(CC1=CC(C(=O)O)=NN1)CC(O)C(C)(C)C	IRAP-A	VISUAL	-16.49	-96.71
	PV-003732599773	CN(CCC1=CC=C2OCOC2=C1)CC1=NC(C(=O)O)=NN1	IRAP-A	VISUAL	-16.04	-96.66
	PV-006829883586	C1=CC=C(C[C@@H]2CN=C(NCC3CCN(C4=CC=NC=N4)CC3)N2)C=C1	IRAP-A	RANDOM	-15.28	-96.56
	Z4250353293	CN(CC1=CC(C(=O)O)=NN1)CC1CC(C(C)(C)C)C1	IRAP-A	RANDOM	-15.24	-96.44

ID	ENAMINE ID	CXSMILES	IRAP state	Selection	Docking score	MM/GBSA score
	Z4250353021	CC1=CC(Cl)=CC(CN(C)CC2=CC(C(=O)O)=NN2)=C1	IRAP-A	RANDOM	-14.91	-96.44
	Z4250353052	CN(CCC1=CC=CC(C(F)(F)F)=C1)CC1=CC(C(=O)O)=NN1	IRAP-A	RANDOM	-15.50	-96.22
	Z4246689204	CCCC(C(=O)NCC1=CC(C(=O)O)=NO1)N(C)C	IRAP-A	VISUAL	-15.62	-96.00
	Z3347742847	O=C(O)C1=NNC(CN2CCC3=CC=C(Cl)C(F)=C3C2)=C1	IRAP-A	RANDOM	-14.91	-95.43
	Z4250350918	CC(C1=CC=C2CCCC2=C1)N(C)CC1=CC(C(=O)O)=NN1	IRAP-A	RANDOM	-15.49	-95.28
	Z5343383448	O=C(O)CCCN1CCN(C(=O)[C@@H]2OCCO[C@@H]2C2=CC=CC=C2)CC1	IRAP-A	RANDOM	-15.66	-94.61
	PV-002993444268	COC1=CC(C)=C2C=C(CNC(C)(C)C3=NNC(C(=O)O)=C3)NC2=C1	IRAP-A	VISUAL	-14.98	-94.58
	Z4251254765	O=C(O)C1=NNC(CN2CCC(O)(CCC(F)(F)F)CC2)=C1	IRAP-A	VISUAL	-15.72	-94.24
	Z4250353463	CC1=CC(Br)=C2OC(CN(C)CC3=CC(C(=O)O)=NN3)CC2=C1	IRAP-A	RANDOM	-15.56	-94.23
	Z4250350170	CN(CC1=CNC=N1)CC1=CC(C(=O)O)=NN1	IRAP-A	VISUAL	-16.44	-93.35
	Z2934194876	CN(CC1=CN=C(C(=O)O)S1)C1CCC2=CC=CC=C2C1	IRAP-A	RANDOM	-15.49	-93.19
	PV-002981194334	CC1=CC=C(C2=CC=CC=C2)C=C1C(=O)NCC1=NNC(C(=O)O)=C1	IRAP-A	RANDOM	-16.51	-93.11
	Z4251134045	CN1C=C(C2CCN(CC3=CN=C(C(=O)O)S3)C2)C2=CC=CC=C21	IRAP-A	RANDOM	-15.68	-92.91
	Z4251281141	CCCC1CN(C2=CC=CC(C(F)(F)F)=C2)CCN1CC1=CC(C(=O)O)=NN1	IRAP-A	VISUAL	-15.97	-92.46
	PV-003830110125	CN(CCC1CCS(=O)(=O)C1)CC1=CC(C(=O)O)=NN1	IRAP-A	VISUAL	-16.58	-92.42
	PV-003616439313	CN(CC1=CC(C(=O)O)=NN1)CC(O)C1(O)CCOC1	IRAP-A	VISUAL	-16.80	-91.80
	Z4251310526	COC1=CC=CC(N2CCN(CC3=CC(C(=O)O)=NN3)CC2C)=C1	IRAP-A	VISUAL	-16.74	-91.61
	Z4210354255	COC1=C(CNCC2=NNC(C(=O)O)=C2)C=CC=C1C(C)(C)C	IRAP-A	RANDOM	-15.23	-91.50
	Z2898216118	CC(CC1=CSC=C1)N(C)CC1=CC(C(=O)O)=NN1	IRAP-A	VISUAL	-15.97	-90.79
	PV-005389180408	CN(CCN1C2=CC=CC3=CC=CC(=C23)S1(=O)=O)CC1=CC(C(=O)O)=NN1	IRAP-A	RANDOM	-16.20	-90.74
	PV-003698827517	CC(C)OC1=NC=C(COC(=O)C2=CC3=C(N2)SC(Cl)=C3)C=N1	IRAP-A	RANDOM	-15.27	-89.44
	Z5341541952	O=C(O)CCCN1CCN(C2=NC(C3=CC=CC=N3)=NC3=C2CCC3)CC1	IRAP-A	RANDOM	-16.45	-89.29
	Z4562164670	CC(C1=NC(C(=O)O)=NN1)N1CCCC(C2=CNC3=CC=CC=C23)C1	IRAP-A	VISUAL	-16.03	-89.00
	PV-002902365021	CCOC(=O)C1=NNC(CN2CC(O)(C(F)(F)C(=O)O)C2)=C1	IRAP-A	VISUAL	-16.30	-88.00
	PV-002711377975	O=C(NCCC(F)(F)C(=O)O)C1=CC2=C(C=C(F)C(F)=C2)N1	IRAP-A	VISUAL	-15.33	-87.81

ID	ENAMINE ID	CXSMILES	IRAP state	Selection	Docking score	MM/GBSA score
	Z4001682850	COC1=CC=CC=C1C1=CCN(CC2=CC(C(=O)O)=NN2)CC1	IRAP-A	VISUAL	-16.88	-87.62
	PV-005438447506	O=C(O)C1=NNC(CN2CCN(C3=NS(=O)(=O)C4=CC=CC=C34)CC2)=C1	IRAP-A	VISUAL	-16.38	-87.13
	PV-003095717604	CC1=CC(C(=O)N(C)CC2=CC(C(=O)O)=NN2)=C2NN=CC2=C1	IRAP-A	RANDOM	-15.64	-87.13
	Z3861465857	O=C(O)C1=NNC(CN2CCC3(CC2)CC2=CC(F)=CC=C2C3=O)=C1	IRAP-A	RANDOM	-16.26	-87.10
	Z4387370311	O=C(O)C1=NNC(CN2CCC(NC3=CC=CC=C3Cl)CC2)=N1	IRAP-A	VISUAL	-15.98	-86.67
	Z4387368384	O=C(O)C1=NNC(CN2CCCC(CC3=CSC4=CC=CC=C34)C2)=N1	IRAP-A	VISUAL	-15.77	-86.28
	Z4251479028	O=C(O)C1=NC=C(CN2CCCN3C(=CC4=CC=CC=C43)C2)S1	IRAP-A	RANDOM	-15.22	-83.97
	Z3508272727	O=C(CC1=CC2=C(C=C1Br)N=CN2)NC1CC=C(C(=O)O)C1	IRAP-A	RANDOM	-14.92	-83.90
	Z5220742121	CN(CC1=NN=C(C(=O)O)S1)C[C@@H](O)C1=CC2=CC=CC=C2O1	IRAP-A	VISUAL	-16.39	-83.60
	PV-006862462825	CC1=NC(CO)=NC=C1C(=O)NCC1=CC=C2SC=C(C)C2=C1	IRAP-A	VISUAL	-15.64	-82.37
	Z4210353093	CC(C)(CNCC1=NNC(C(=O)O)=C1)CC1=CC=CC=C1Br	IRAP-A	RANDOM	-16.10	-82.04
	Z3510007969	CC1=CC2=C(NN=C2)C(C(=O)N(C)C2(CC(=O)O)CC2)=C1	IRAP-A	RANDOM	-15.48	-81.93
	PV-003860597259	CC1=CC(F)=CC=C1CS(=O)(=O)NCC1=NNC(C(=O)O)=C1	IRAP-A	VISUAL	-15.62	-80.16
	Z4250578603	CC1=CC=C(N2CCN(CC3=CC(C(=O)O)=NN3)CC2)C(C)=C1	IRAP-A	RANDOM	-16.77	-78.74
	PV-003014300839	CC1=CN(CC(=O)NC(C)(C)C2=NNC(C(=O)O)=C2)C2=CC=CC=C12	IRAP-A	RANDOM	-14.92	-78.53
	Z5286457552	CC1=CC(C(=O)O)=NC=C1CN1CCC(C2=CC=CC(C#N)=C2)C1	IRAP-A	RANDOM	-15.22	-77.35
	Z3510003749	CN(C(=O)C1=CC(Br)=CC2=C1N=CN2)C1(CC(=O)O)CC1	IRAP-A	RANDOM	-15.24	-76.26
	PV-006260900716	CC12CN(CC3=CC=CC=C3)CC1(C)CN(C(=O)COC1=CC(CN)=CC=C1)C2	IRAP-B	RANDOM	-15.17	-150.77
	Z4175111789	COC1=CC=C(C(=O)NC2CCN(C(C)C3=CC=NC=C3)CC2)C=C1OCCN	IRAP-B	VISUAL	-15.38	-126.75
	Z4250310204	CC1=CC=C(N2C=CC(CN3CCC(C4=C(C(=O)O)N=CO4)CC3)=N2)C=C1	IRAP-B	RANDOM	-15.35	-119.04
	PV-005636810118	CC1=C(C)C(C(=O)NC[C@@H]2[C@H]3C[C@H]3CN2C(=O)CC2CCN(C)CC2)=NN1	IRAP-B	RANDOM	-14.76	-118.41
	Z5592194898	CC1=CC=C2NC(CNC[C@@H]3[C@H]4CC[C@H]4[C@@H]3C(=O)O)=CC2=C1C	IRAP-B	RANDOM	-16.22	-117.95
	Z5074742728	CC(NC(=O)[C@H](CC(=O)O)N(C)C)C1=CC=CC2=C1NC=C2	IRAP-B	VISUAL	-15.98	-116.90
	PV-006283977818	O=C(O)CC(NCC1=CC2=C(N1)C(Br)=CS2)C1=CC=NC=C1	IRAP-B	VISUAL	-14.97	-116.57
6	Z4380815781	CC1=CC=C2NC(CN[C@@H]3C[C@H](C(=O)O)C34CCC4)=CC2=C1C	IRAP-B	RANDOM	-14.96	-116.11

ID	ENAMINE ID	CXSMILES	IRAP state	Selection	Docking score	MM/GBSA score
	**PV-003315399085**	**NC1=NC=C(CNCC2=CC3=CC=C(Cl)C=C3N2)C(N)=N1**	IRAP-B	VISUAL	-14.91	-115.60
	**Z4001220567**	**CCC1=CC=C(C2=NC(CN3CCC(C4=CNN=C4C(=O)O)CC3)=C(C)O2)C=C1**	IRAP-B	RANDOM	-16.08	-113.74
	**Z3879747050**	**COC(=O)C1(C2=CC=CC=C2Cl)CCN(CC2=CC(=O)N3N=CC=C3N2)C1**	IRAP-B	RANDOM	-15.27	-113.62
	**PV-006005181456**	**O=C(NCC(=O)N1C[C@@H]2C[C@@H]2[C@H]1CNC(=O)C1=NNC=C1)NC1CCCCC1**	IRAP-B	VISUAL	-15.68	-113.07
	**PV-006383708231**	**CC1CCN(CC(=O)N2CCC(C3CCN(C(=O)C4=CC=NC=N4)C3)C2)CC1**	IRAP-B	RANDOM	-15.23	-112.65
	**PV-005433944246**	**CC1=CC(Br)=CC2=C1C=C(CNC(CC(=O)O)C1=CC=NC=C1)N2**	IRAP-B	VISUAL	-15.75	-111.53
	**PV-003186566901**	**CC1=CNN=C1C(=O)N[C@@H]1C[C@@H]1CNC(=O)CN1CCC(C)(C)C1**	IRAP-B	VISUAL	-16.02	-111.19
7	**Z4001220365**	**O=C(O)C1=NNC=C1C1CCN(CC2=NOC(C3=CC=C(Cl)C=C3)=C2)CC1**	IRAP-B	RANDOM	-16.03	-110.72
	**PV-006884085939**	**COC1=NC=CC=C1CN(C)CC1=CC2=CC=CC=C2C(=O)N1**	IRAP-B	VISUAL	-15.55	-110.22
	**Z4115949382**	**CC1=C(CCNC(=O)C(C#N)C2CCNCC2)C2=C(Cl)C=CC=C2N1**	IRAP-B	RANDOM	-15.34	-109.44
	**PV-006805391731**	**COC1=NC=CC=C1CN(C)CC1=CC2=C(C=CC=C2F)N1**	IRAP-B	RANDOM	-16.46	-109.31
	**Z4081743610**	**CC1=CC=C2NC(CN[C@@H]3C[C@H](O)[C@H]3CC(=O)O)=CC2=C1C**	IRAP-B	VISUAL	-16.62	-109.24
	**PV-005727181030**	**CC1=NC(C(=O)NCC2(CNC(=O)C3CC4=NN=C(C)N4C3)CC2)=C(C)N1**	IRAP-B	RANDOM	-16.35	-109.14
	**PV-002779753296**	**CC(C1=NN=CN1)N1CCC(C2=NNC(N)=N2)C1**	IRAP-B	VISUAL	-15.30	-108.91
	**PV-005970070526**	**CN(CCNC(=O)C1=NNC2=CC=C(Cl)C=C12)C(=O)CC1CCCC(O)C1**	IRAP-B	RANDOM	-15.14	-108.91
	**PV-003062318855**	**O=C(O)CC1(C(=O)O)CCN(CC2=NC3=C(COCC3)N2)C1**	IRAP-B	VISUAL	-15.24	-108.60
	**PV-003431445904**	**CN(CC1=CC(C(=O)O)=NN1)CC1=NC2=C(C=CC=C2F)N1**	IRAP-B	VISUAL	-15.98	-108.48
	**PV-004495093970**	**CC1=NC(CC(=O)N2CC(CNC(=O)C3=NNC4=C3CCC4)C(C)(C)C2)=CN1**	IRAP-B	RANDOM	-14.96	-108.32
8	**Z2898226219**	**O=C(O)C1=NNC(CN2CCCCC2CCCC2=CC=CC=C2)=C1**	IRAP-B	VISUAL	-14.97	-107.79
	**PV-006963367379**	**COC1=NC=CC=C1CN(C)CC1=CC2=CC=C(C)C=C2N1**	IRAP-B	RANDOM	-15.93	-107.57
	**PV-007010261716**	**CC1=NC=CC(CCNC(=O)C2=NNC3=C2CCCCC3)=N1**	IRAP-B	RANDOM	-15.80	-107.43
	**PV-005466392658**	**CC1=NN=C2CC(C(=O)NCC(C)(C)CNC(=O)C3=CC4=C(C=CO4)N3)CN12**	IRAP-B	VISUAL	-14.82	-106.44
	**PV-003111086729**	**CNC(=O)N[C@H](C)C(=O)N(C)CCNC(=O)C1=CC=CN1**	IRAP-B	VISUAL	-15.28	-106.37
	**PV-007037204777**	**CC1=CC=C2C(C(=O)NCC(F)CNC(=O)C3=CC=C(F)O3)=NNC2=C1**	IRAP-B	VISUAL	-15.51	-106.30
	**PV-004482068029**	**C[C@@H](CNC(=O)C1=NNC2=C1CC(F)(F)CC2)N(C)C(=O)CN1C=CN=N1**	IRAP-B	VISUAL	-14.74	-105.67

ID	ENAMINE ID	CXSMILES	IRAP state	Selection	Docking score	MM/GBSA score
	PV-007017201417	COC1=NC=CC=C1CN(C)CC1=C(Br)C2=CC=CC=C2N1	IRAP-B	RANDOM	-15.13	-105.18
	Z3347802976	CC(C)(C)CCC1CCN(CC2=CC(C(=O)O)=NN2)C1	IRAP-B	RANDOM	-14.75	-105.14
	PV-005022891113	C[C@H](CNC(=O)C1=CC(=O)NN=C1)CNC(=O)C1CCC2=NC=CC=C2C1	IRAP-B	VISUAL	-16.02	-103.42
	PV-004158159053	O=C(CC1=C2NC3=C(C=CC=C3Cl)C2=CC=C1)NCC1=NNC(C(=O)O)=C1	IRAP-B	VISUAL	-15.61	-101.78
	PV-002939007821	CC1=NC=C(NCCC2=CC3=CC=CC=C3N2)C(C#N)=N1	IRAP-B	VISUAL	-15.45	-101.48
	PV-003467682774	CC1=NNC(C(=O)N[C@@H]2CC[C@H]2CNC(=O)CC2=CC=NO2)=C1Cl	IRAP-B	VISUAL	-15.17	-101.14
	Z4240409427	O=C(NCC(CCO)C1=CC=NC=C1)C1=NC(Br)=CN1	IRAP-B	VISUAL	-15.81	-101.13
	Z5394358312	BrC1=CC=C2C=C(CNC3CCCC4=C3N=CN4)NC2=C1	IRAP-B	RANDOM	-15.15	-100.70
	PV-007043660508	CC1=NC(C(C)(C)CNC(=O)C2=CC=C(CN(C)C)N2)=NN1	IRAP-B	VISUAL	-14.95	-100.46
	PV-006853346153	CN(C)[C@@H]1CCC2=CC=CC=C2[C@H]1NC(=O)CNC(=O)C1=CC2=CC=CC=C2N1	IRAP-B	RANDOM	-15.42	-100.40
	PV-005372643423	CC1=C(C(=O)NC[C@H](O)CNC(=O)C2=CC=NN=C2)NC2=CC(F)=CC(F)=C12	IRAP-B	VISUAL	-15.26	-100.02
	Z4250350170	CN(CC1=CNC=N1)CC1=CC(C(=O)O)=NN1	IRAP-B	VISUAL	-16.02	-99.94
	PV-006802194032	CC1=CC=C(C)C(S(=O)(=O)NCC(=O)NCC2=CC3=C(C=C(C)S3)N2)=C1	IRAP-B	VISUAL	-15.21	-99.57
	Z5292279984	CSC1(C(=O)O)CC(NC(=O)C2=C(C3CC3)C=NN2)C1	IRAP-B	VISUAL	-15.68	-99.42
	PV-006781773614	O=C(CCNC(=O)C1=C2C=CC=CC2=NN1)NCC1=CC=C(O)C(F)=C1F	IRAP-B	RANDOM	-15.41	-99.35
	Z4210353253	CCNC1=CC=CC=C1CNCC1=NNC(C(=O)O)=C1	IRAP-B	VISUAL	-14.75	-99.27
	PV-004332340507	CC1=C(C(=O)NCC2(O)CN(C(=O)C(C)N3CCC3)C2)NN=C1C(F)(F)F	IRAP-B	VISUAL	-15.89	-99.11
	PV-003405258552	CN(CCNC(=O)C1=C(C2CC2)C=NN1)C(=O)C1(C)CCCC1O	IRAP-B	VISUAL	-15.16	-97.26
	PV-002887518540	O=C(NCC(CCO)C1=CC=NC=C1)C1=NNC=N1	IRAP-B	VISUAL	-14.95	-96.68
	Z4240408572	CC1=CC=CC2=C1NN=C2C(=O)NCC(CCO)C1=CC=NC=C1	IRAP-B	RANDOM	-16.72	-96.44
9	PV-002947517349	O=C(NCCC(C(=O)O)C1=CNN=C1)C1=C2CCCCC2=CN1	IRAP-B	VISUAL	-16.31	-96.38
	Z3609506103	CN(CC1=CN=C(N2C=CC=N2)S1)C(=O)CCC1=C2CCCCC2=NN1	IRAP-B	RANDOM	-14.75	-95.52
	PV-007108701995	O=C(NCCC1=CC(=O)NC(C2=CC=CC=N2)=N1)C1=NNC2=CC=C(Cl)C=C12	IRAP-B	RANDOM	-15.31	-95.34
	PV-006334475960	CC(NC(=O)C1=C2C=CC=CC2=NN1)C(=O)N1C[C@@H]2CCCN2C[C@H]1C(C)C	IRAP-B	RANDOM	-16.49	-94.00
	PV-005133931482	CN1CCCC1(C)C(=O)N1CC(CO)(NC(=O)C2=NNC(C3=CC=CO3)=C2)C1	IRAP-B	VISUAL	-15.83	-91.99

ID	ENAMINE ID	CXSMILES	IRAP state	Selection	Docking score	MM/GBSA score
	**PV-002908100897**	**CC1=NC(C2=NC=CC=N2)=NC=C1C(C)NCCC1=CC=CC2=C1NC=C2**	IRAP-B	VISUAL	-15.57	-91.40
	**PV-004160528578**	**CC(C)(CNC(=O)CC1=CN=C2C=CC=NN12)NC(=O)C1=NNC2=C1CCCC2**	IRAP-B	VISUAL	-15.33	-90.67
	**PV-006943073003**	**O=C(NCC(CCO)C1=CC=NC=C1)C1=NNC2=CC=C(Br)C=C12**	IRAP-B	RANDOM	-16.69	-89.93
	**PV-006744440553**	**CC(CC1=CC=C2NN=CC2=C1)NC(=O)C1=C(C2CC2)C=CN1**	IRAP-B	RANDOM	-14.96	-87.69
	**PV-003208817841**	**O=C(NCC(CCO)C1=CC=NC=C1)C1=NNC2=C1CCCN2**	IRAP-B	VISUAL	-17.30	-87.64
	**PV-006848007303**	**CC1=NC=CC(CCNC(=O)C2=NNC3=CC=C(Cl)C=C23)=N1**	IRAP-B	RANDOM	-16.36	-86.96
	**Z1951089022**	**CN(CC1=NNC2=CC=CC=C12)CC1=CC=NN1C**	IRAP-B	RANDOM	-15.28	-86.85
	**Z4507718605**	**CC(C1=NNN=N1)N(CC1=NNC2=CC=CC=C12)C1CC1**	IRAP-B	RANDOM	-15.25	-86.64
	**PV-006369094312**	**CC(C)C1=CC(C(=O)N2CCN(C(=O)C3=NC=NC4=C3CCC4)CC2)=CC(O)=N1**	IRAP-B	RANDOM	-15.38	-86.31
	**Z4507718605**	**CC(C1=NNN=N1)N(CC1=NNC2=CC=CC=C12)C1CC1**	IRAP-B	RANDOM	-15.25	-85.67
	**PV-005497177697**	**COCC1=C(C(=O)NCC2(C)CN(C(=O)C3=CC(C)=CN3)C(C)(C)C2)NC=C1**	IRAP-O	RANDOM	-15.33	-165.57
	**Z4115312583**	**CC(C1=CC=CC=N1)N(C)CCNC(=O)C1=CC(C2CCCN2)=NN1**	IRAP-O	VISUAL	-15.14	-134.59
**10**	**Z3386081457**	**CN1C=CC(C(NC(=O)C2=CC(C3CCNC3)=NN2)C2=CC=CC=C2)=N1**	IRAP-O	RANDOM	-14.70	-127.84
**11**	**PV-003267964142**	**NCC1=NNC(C(=O)NC(CC2=CC=CC=C2Cl)C2=CC=CC=C2)=C1**	IRAP-O	VISUAL	-15.17	-122.42
**12**	**Z4309616340**	**CC(CNC(=O)C1=CC=C2C(=O)NN=CC2=C1)N1CCN(C2=CC=CC=C2)CC1**	IRAP-O	RANDOM	-15.31	-121.16
**13**	**Z3385671698**	**CN(C)CC1=NC2=CC=C(NC(=O)C3=CC=CC4=C3CNCCO4)C=C2N1**	IRAP-O	RANDOM	-15.97	-120.85
	**PV-005804701749**	**C=C(Cl)C(C)(C)C(=O)N1CC(N2CCN(C(=O)CC3=CNN=C3C)CC2)C1**	IRAP-O	RANDOM	-16.01	-119.86
**14**	**Z5518863768**	**CN(C)C(CNC(=O)CC1=CC=C(C2=NN=CN2)C=C1)C1=CC=CC=C1Cl**	IRAP-O	RANDOM	-14.88	-119.75
	**Z5282597398**	**O=C(NCC1=CC2=CC=CN=C2N1)C1=C(C2CNC2)NN=C1**	IRAP-O	VISUAL	-15.22	-118.51
	**Z4272410596**	**CC1=CC=CC=C1CN1CCC(NC(=O)CN2CCC3=C(C=NNC3=O)C2)CC1**	IRAP-O	VISUAL	-16.44	-118.45
	**PV-005690321219**	**CCCCC(=O)C1=CC=C(NC(=O)N2CC3=C(C2)C(=O)NC(=O)N3)C=C1Cl**	IRAP-O	RANDOM	-14.95	-118.29
**15**	**Z4601470241**	**CC(CNC(=O)N1CC2=C(C1)C(=O)NC=N2)N1CCN(C2=CC=CC=C2)CC1**	IRAP-O	RANDOM	-15.12	-118.01
	**PV-003992050528**	**NC(CC1=CNN=C1)C(=O)NC(C1=CC=CC=C1)C1=CC=C2NC=NC2=C1**	IRAP-O	VISUAL	-15.36	-117.87
	**PV-004560032796**	**CC1(C)CCCC(C)(C)C1C(=O)NC1CN(C(=O)CC2=CC(=O)NC(=O)N2)C1**	IRAP-O	RANDOM	-14.95	-117.64
	**PV-005668578965**	**CN(C)[C@H](CO)C(=O)N1CCC2=CC=CC=C2C1CNC(=O)C1=CC(F)=CN1**	IRAP-O	VISUAL	-16.65	-117.28

ID	ENAMINE ID	CXSMILES	IRAP state	Selection	Docking score	MM/GBSA score
	PV-005422025641	CCOC1(C(=O)N2CC(N3CCN(C(=O)CC4=CNN=C4C)CC3)C2)CCC1	IRAP-O	RANDOM	-14.90	-116.84
16	PV-002764876330	CC(NC(=O)C1=NNC(C2=CN(C)N=C2)=C1)C(N)C1=CC=CO1	IRAP-O	VISUAL	-14.87	-115.97
	PV-006855251261	CC(O)(CNC(=O)C1=CC(F)=CN1)CNC(=O)C1CN2CCC1CC2	IRAP-O	VISUAL	-15.55	-114.68
	Z4309751849	CC1CCCN(C(C)(C)CNC(=O)C2=CC=C3C(=O)NN=CC3=C2)C1	IRAP-O	VISUAL	-15.71	-114.59
	PV-004651371208	CC1=CC=C(SCC(=O)N[C@H]2C[C@@H](O)[C@H](NC(=O)C3=CNN=C3)C2)C=C1C	IRAP-O	VISUAL	-15.70	-114.48
17	PV-006830307217	CC1=CC=C(CC(C)(CO)NC(=O)C2=NNC(C3=CNC4=CC=CC=C34)=C2)C=C1	IRAP-O	VISUAL	-14.71	-114.32
	PV-006906684662	CC1=CN=C(C)C(C(=O)NCC(C)(O)CNC(=O)C2=NNC=C2F)=C1	IRAP-O	VISUAL	-14.70	-113.55
	PV-005097643364	CCC1=CNN=C1C(=O)NC1CCC(CNC(=O)C2=CN=C3NC=CC3=C2)CC1	IRAP-O	VISUAL	-16.03	-113.03
	PV-006074765607	CC1=NNC(C)=C1CCC(=O)N1CCC2(CCCC2NC(=O)C2=CN=CN2)C1	IRAP-O	RANDOM	-14.89	-113.02
	Z4272410601	O=C(CN1CCC2=C(C=NNC2=O)C1)NCC1=CC=C(CN2CCCC2)C=C1	IRAP-O	RANDOM	-15.40	-112.40
	PV-004990993519	O=C(NC1=NN=C(N2CCCCC2)N1)C1=CC=C2C(=O)NN=CC2=C1	IRAP-O	RANDOM	-15.15	-111.20
	PV-003666315616	NCC1=NNC(C(=O)NC(C2=CC=CC=C2)C2=CC=CC3=CC=CC=C23)=C1	IRAP-O	VISUAL	-15.33	-111.18
	PV-006376531261	CC(C)(CNC(=O)C1=CC=CN1)CC1CN(C(=O)C2CCCC3=C2C=NN3)C1	IRAP-O	VISUAL	-16.05	-110.03
	PV-005804701749	C=C(Cl)C(C)(C)C(=O)N1CC(N2CCN(C(=O)CC3=CNN=C3C)CC2)C1	IRAP-O	VISUAL	-14.71	-109.98
	PV-004452500357	CCCN(C)CC1=CC=C(C(=O)N2CC3=C(C2)C(=O)NC(=O)N3)O1	IRAP-O	VISUAL	-16.22	-109.84
	PV-007050107558	CC1=C(C(=O)N2CC3=C(C2)C(=O)NC(=O)N3)C=NN1C1=CC=C(C(C)(C)C)C=C1	IRAP-O	VISUAL	-14.71	-109.41
	Z4472167234	CN1CCN(C(=O)C2=CC(C3=CC=C(O)C=C3)=NN2)CC1C1=CC=CS1	IRAP-O	RANDOM	-15.07	-109.29
	PV-006828053122	CC1=NNC(C)=C1CC(C)C(=O)N[C@@H]1C[C@H]2C[C@@H](NC(=O)C3=CC=CN3)[C@H]2C1	IRAP-O	VISUAL	-17.30	-108.43
	Z4312297770	C[C@H]1C[C@@H](NC(=O)C2=CC=C3C(=O)NN=CC3=C2)C[C@@H](C)N1CC1=CC=CC=C1	IRAP-O	RANDOM	-14.87	-107.84
	PV-005717770058	CC(=O)C1=CNC(C(=O)N[C@@H](C)C2CCN(C(=O)C3=C(Cl)C=NN3)CC2)=C1	IRAP-O	VISUAL	-14.86	-107.35
	PV-006337896860	CC1CCC2=C(C=C(C(=O)NCC(=O)NC3=CC4=NNC=C4C=C3)N2)C1	IRAP-O	VISUAL	-15.19	-107.26
	PV-004762123740	CC(C)(O)C1(CNC(=O)C2=CC=CN2)CN(C(=O)CC2=CC=C3C=NNC3=C2)C1	IRAP-O	VISUAL	-15.73	-107.15
	PV-004393531270	O=C(N[C@H]1C[C@@H]1NC(=O)C1=CC(Cl)=CC2=CC=NC=C12)C1=NC=NC(O)=C1	IRAP-O	RANDOM	-14.95	-106.35
	PV-004233542396	COC1=CC(C(=O)N[C@H]2COC[C@H]2NC(=O)C2=CN=CN2)=C2N=CC=CC2=C1	IRAP-O	RANDOM	-15.18	-105.88
	PV-007117827737	O=C(N[C@@H]1CC[C@H]1NC(=O)C1=CC(F)=CN1)C1=CC=C2C(=O)NN=CC2=C1	IRAP-O	VISUAL	-16.27	-104.62

ID	ENAMINE ID	CXSMILES	IRAP state	Selection	Docking score	MM/GBSA score
	PV-006555934998	CC(C)<br>(C(=O)N[C@H]1C[C@H](NC(=O)C2=C(F)NN=C2)C1)C1=CNC2=CC=CC=C12	IRAP-O	VISUAL	-15.60	-104.50
	PV-002853724860	CN1CCCC2=CC=C(NC(=O)N3CC4=C(C3)C(=O)NC(=O)N4)C=C2C1	IRAP-O	VISUAL	-16.03	-104.36
	PV-004350213648	COCC1=C(C(=O)N2C[C@@H]3C[C@@H]3[C@@H](NC(=O)C3=CC(C#N)=CN3)C2)NC=C1	IRAP-O	VISUAL	-15.35	-104.19
	Z4225996356	O=C(CN1CC2CCC1C2)N1CCC2=C(C=NNC2=O)C1	IRAP-O	RANDOM	-15.24	-104.04
	PV-003325676028	ClC1=CC=C(C2=NNC(CNCC3=NC4=CC=CC=C4N3)=C2)C=C1	IRAP-O	VISUAL	-14.72	-103.60
	PV-003036335116	O=C(NCC1=CNC2=CC=C(F)C=C12)N1CC2=C(C1)C(=O)NC(=O)N2	IRAP-O	VISUAL	-16.18	-103.25
	PV-006897939853	CC(O)(CNC(=O)C1=CC(F)=CN1)CNC(=O)[C@@H]1CC[C@H]2C[C@@H]1CO2	IRAP-O	VISUAL	-15.58	-103.04
	Z4879764433	O=C(CN1CCCC(OCC(F)F)C1)N1CC2=C(C1)C(=O)NC(=O)N2	IRAP-O	VISUAL	-15.55	-102.71
	PV-004595861284	O=C(N[C@H]1CCN(C2=CC(=O)NC(=O)N2)C1)C1=CC=C(CCC2=CC=CC=C2)N1	IRAP-O	VISUAL	-15.75	-102.25
	Z4828721937	CC(CCCC(=O)N1CC2=C(C1)C(=O)NC(=O)N2)C1=CC=CC=C1	IRAP-O	RANDOM	-15.31	-101.19
	PV-006677779485	CC1=COC(C(=O)N2CCC(N3CCN(C(=O)CC4=CNN=C4C)CC3)C2)=C1	IRAP-O	RANDOM	-15.11	-101.07
	PV-004715269800	O=C(NC[C@@H]1CC(F)(F)CN1C(=O)[C@H]1C[C@H]1F)C1=CC=C2C(=O)NN=CC2=C1	IRAP-O	RANDOM	-14.86	-100.95
	Z4594993615	O=C(CCCN1C(=O)CCC2=CC=CC=C21)N1CC2=C(C1)C(=O)NC=N2	IRAP-O	RANDOM	-15.27	-100.87
	PV-006229431204	CC(C)(C)OC(=O)N1CC2CCC2(CC(=O)N2CC3=C(C2)C(=O)NC(=O)N3)C1	IRAP-O	RANDOM	-15.32	-100.48
	PV-006956939305	CC1=CC=C(N2NC(C(=O)N=S(C)(=O)C3=CNN=C3)=CC2=O)C=C1C	IRAP-O	RANDOM	-14.91	-95.84
	PV-006845993993	CS(=O)(=NC(=O)C1=CC=C(C2=CC=C(Cl)C=C2)N1)C1=CNN=C1	IRAP-O	VISUAL	-15.18	-95.12
	Z4154684703	O=C(COCC1=CC(=O)NC=C1)NC1C2=CC=CC=C2C2CCC1C2	IRAP-O	RANDOM	-14.72	-93.00
	PV-003015074737	O=C(NC1=NN(C2=CC=CC=N2)C=C1)C1=CC=C2C(=O)NN=CC2=C1	IRAP-O	RANDOM	-14.91	-92.59
	PV-004476105682	CCC1=C(C(=O)N[C@H]2C[C@H](NC(=O)C3=CC(C)=NN3C3=CC=CC=N3)C2)C=NN1	IRAP-O	RANDOM	-15.10	-89.65
	Z3413964093	CCC1=NN=C(C2CN(C(=O)NC3CCN(CCF)C3=O)CC3=CNN=C32)N1	IRAP-O	VISUAL	-14.70	-83.45

Table S2: Enalos Suite predicted pharmacokinetic (PK) parameters for all the 240 compounds selected for Molecular Dynamics / MM-GBSA calculations. The predictions include the classification for cytotoxicity (active/inactive) and mutagenicity (negative/positive), the logBCF (bioconcentration factor), logP (lipophilicity) and logS(solubility). The initial selection method (visual selection or random selection after Enalos filtering) is mentioned. Out of the 240 compounds, a subset of 124 were chosen by visual investigation, and a second subset of 116 were selected randomly after having applied filters for three of the predicted PK parameters (see Methods Section). The screening cavity of IRAP from which the compounds originated is also shown.

Ordered No.	Enamine ID	IRAP state	Selection	Cytotoxicity Classification	MSlogBCF	MSlogP	MSlogS	Mutagenicity Classification
	**Z3861670572**	IRAP-A	VISUAL	"inactive"	2.253	3.034	-3.612	"negative"
	**Z4250350143**	IRAP-A	VISUAL	"inactive"	0.257	0.208	-2.221	"negative"
1	**Z5341541922**	IRAP-A	ENALOS	"inactive"	0.831	2.035	-3.830	"negative"
	**Z3838877942**	IRAP-A	VISUAL	"inactive"	1.696	1.812	-4.571	"positive"
	**Z5422924976**	IRAP-A	ENALOS	"inactive"	0.965	2.376	-3.871	"negative"
	**Z5341541542**	IRAP-A	ENALOS	"inactive"	0.801	2.154	-4.385	"negative"
	**Z4210355805**	IRAP-A	VISUAL	"inactive"	1.563	1.829	-3.893	"negative"
	**Z4212262952**	IRAP-A	VISUAL	"inactive"	1.365	0.457	-3.710	"negative"
	**PV-004727649566**	IRAP-A	VISUAL	"inactive"	0.482	-0.902	-0.999	"negative"
	**Z4562168048**	IRAP-A	ENALOS	"inactive"	0.982	1.708	-2.857	"negative"
	**PV-003401519556**	IRAP-A	VISUAL	"inactive"	0.250	-0.432	-2.342	"negative"
	**Z4250065604**	IRAP-A	VISUAL	"inactive"	0.953	-0.664	-3.299	"positive"
2	**Z5179432492**	IRAP-A	ENALOS	"inactive"	1.977	2.104	-3.754	"negative"
	**Z5269220534**	IRAP-A	ENALOS	"inactive"	1.575	2.006	-4.659	"negative"
	**Z4387367234**	IRAP-A	VISUAL	"inactive"	0.907	0.520	-3.119	"positive"
	**Z4247724324**	IRAP-A	VISUAL	"inactive"	1.242	0.833	-3.317	"positive"
	**Z4210353111**	IRAP-A	ENALOS	"inactive"	1.327	1.852	-2.661	"negative"
3	**Z3390553169**	IRAP-A	ENALOS	"inactive"	1.154	1.804	-3.256	"negative"
	**PV-003310368609**	IRAP-A	VISUAL	"inactive"	0.215	-0.230	-1.450	"negative"

Ordered No.	Enamine ID	IRAP state	Selection	Cytotoxicity Classification	MSlogBCF	MSlogP	MSlogS	Mutagenicity Classification
4	**Z3861671484**	IRAP-A	VISUAL	"inactive"	1.378	0.656	-4.229	"negative"
	**Z4387366308**	IRAP-A	VISUAL	"inactive"	1.049	1.251	-2.202	"negative"
	**Z4250158149**	IRAP-A	ENALOS	"inactive"	0.912	2.134	-1.874	"negative"
	**PV-004622855944**	IRAP-A	ENALOS	"inactive"	1.123	2.143	-4.014	"negative"
5	**Z4250351400**	IRAP-A	ENALOS	"inactive"	1.230	1.594	-3.937	"negative"
	**Z4381214573**	IRAP-A	VISUAL	"inactive"	1.378	0.745	-1.887	"positive"
	**Z2898202771**	IRAP-A	ENALOS	"inactive"	1.451	1.709	-1.897	"negative"
	**PV-002804155214**	IRAP-A	VISUAL	"inactive"	0.256	0.625	-1.985	"negative"
	**Z4387334824**	IRAP-A	VISUAL	"inactive"	0.844	1.375	-3.712	"negative"
	**Z4251430825**	IRAP-A	ENALOS	"inactive"	0.482	2.015	-3.850	"negative"
	**PV-002967423450**	IRAP-A	VISUAL	"inactive"	0.938	1.138	-3.446	"positive"
	**Z4387360883**	IRAP-A	VISUAL	"inactive"	0.885	1.281	-3.640	"negative"
	**PV-004713760776**	IRAP-A	ENALOS	"inactive"	0.897	2.060	-6.125	"negative"
	**PV-007066194544**	IRAP-A	ENALOS	"inactive"	1.069	2.273	-4.190	"negative"
	**Z4001682850**	IRAP-A	ENALOS	"inactive"	1.204	1.592	-2.124	"negative"
	**Z4250353294**	IRAP-A	VISUAL	"inactive"	0.469	0.114	-2.039	"negative"
	**PV-002804155214**	IRAP-A	VISUAL	"inactive"	0.255	0.620	-1.989	"negative"
	**Z3777363876**	IRAP-A	ENALOS	"inactive"	0.865	2.005	-3.146	"negative"
	**PV-003310368609**	IRAP-A	VISUAL	"inactive"	0.277	-0.230	-1.451	"negative"
	**Z4210355917**	IRAP-A	VISUAL	"inactive"	0.972	1.567	-2.272	"positive"
	**Z2911606713**	IRAP-A	ENALOS	"inactive"	1.111	2.066	-4.048	"negative"
	**Z4251268391**	IRAP-A	VISUAL	"inactive"	1.626	1.561	-3.702	"negative"
	**Z2898236840**	IRAP-A	ENALOS	"inactive"	1.273	2.187	-2.469	"negative"
	**Z4341677602**	IRAP-A	VISUAL	"inactive"	1.158	1.042	-2.968	"positive"
	**PV-002781370939**	IRAP-A	VISUAL	"inactive"	0.444	-1.027	-1.937	"positive"
	**Z4562167868**	IRAP-A	VISUAL	"inactive"	1.654	-0.497	-2.894	"positive"

Ordered No.	Enamine ID	IRAP state	Selection	Cytotoxicity Classification	MSlogBCF	MSlogP	MSlogS	Mutagenicity Classification
	**PV-006852601960**	IRAP-A	VISUAL	"inactive"	0.523	0.186	-1.577	"positive"
	**Z3655568854**	IRAP-A	ENALOS	"inactive"	1.613	1.508	-4.976	"negative"
	**Z4210355082**	IRAP-A	ENALOS	"inactive"	1.097	1.645	-4.391	"negative"
	**Z5182662261**	IRAP-A	VISUAL	"inactive"	1.198	2.634	-4.890	"negative"
	**Z5434197110**	IRAP-A	VISUAL	"inactive"	1.130	1.381	-2.369	"negative"
	**PV-003730731431**	IRAP-A	VISUAL	"inactive"	0.325	-1.257	-1.474	"negative"
	**Z4168669561**	IRAP-A	ENALOS	"inactive"	1.166	2.249	-4.668	"negative"
	**Z4388146723**	IRAP-A	ENALOS	"inactive"	0.937	1.703	-2.552	"negative"
	**Z3347815877**	IRAP-A	ENALOS	"inactive"	1.192	1.683	-2.953	"negative"
	**Z4250352285**	IRAP-A	VISUAL	"inactive"	0.279	-0.517	-1.447	"negative"
	**PV-003948874842**	IRAP-A	ENALOS	"inactive"	1.241	2.643	-5.423	"negative"
	**PV-002981194334**	IRAP-A	ENALOS	"inactive"	1.854	2.239	-4.762	"negative"
	**PV-007098233941**	IRAP-A	VISUAL	"inactive"	1.315	1.865	-3.654	"negative"
	**PV-005890634210**	IRAP-A	ENALOS	"inactive"	0.976	1.940	-4.276	"negative"
	**Z4387360928**	IRAP-A	VISUAL	"inactive"	1.339	0.295	-2.191	"positive"
	**PV-005654514600**	IRAP-A	ENALOS	"inactive"	0.988	2.504	-5.188	"negative"
	**Z4562164875**	IRAP-A	ENALOS	"inactive"	1.404	1.548	-3.726	"negative"
	**Z3347937904**	IRAP-A	ENALOS	"inactive"	0.854	3.331	-3.747	"negative"
	**Z4210354573**	IRAP-A	ENALOS	"inactive"	1.203	1.662	-3.382	"negative"
	**PV-002969507796**	IRAP-A	VISUAL	"inactive"	0.889	1.646	-2.193	"negative"
	**Z4000894115**	IRAP-A	ENALOS	"inactive"	0.869	2.345	-2.211	"negative"
	**Z3635314177**	IRAP-A	VISUAL	"inactive"	0.400	-1.597	-1.438	"negative"
	**PV-003732599773**	IRAP-A	VISUAL	"inactive"	0.942	0.265	-2.668	"positive"
	**PV-006829883586**	IRAP-A	ENALOS	"inactive"	1.655	1.829	-4.372	"negative"
	**Z4250353293**	IRAP-A	ENALOS	"inactive"	0.431	1.894	-1.746	"negative"
	**Z4250353021**	IRAP-A	ENALOS	"inactive"	1.529	1.934	-3.037	"negative"

Ordered No.	Enamine ID	IRAP state	Selection	Cytotoxicity Classification	MSlogBCF	MSlogP	MSlogS	Mutagenicity Classification
	**Z4250353052**	IRAP-A	ENALOS	"inactive"	1.288	1.965	-2.433	"negative"
	**Z4246689204**	IRAP-A	VISUAL	"inactive"	0.274	-1.114	-1.481	"positive"
	**Z3347742847**	IRAP-A	ENALOS	"inactive"	1.894	2.422	-3.429	"negative"
	**Z4250350918**	IRAP-A	ENALOS	"inactive"	1.102	1.854	-3.661	"negative"
	**Z5343383448**	IRAP-A	ENALOS	"inactive"	0.894	1.648	-3.261	"negative"
	**PV-002993444268**	IRAP-A	VISUAL	"inactive"	1.175	0.750	-5.357	"negative"
	**Z4251254765**	IRAP-A	VISUAL	"inactive"	0.356	-0.971	-1.566	"negative"
	**Z4250353463**	IRAP-A	ENALOS	"inactive"	1.499	1.711	-3.971	"negative"
	**Z4250350170**	IRAP-A	VISUAL	"inactive"	0.322	-0.786	-1.562	"negative"
	**Z2934194876**	IRAP-A	ENALOS	"inactive"	1.205	2.106	-2.613	"negative"
	**PV-002981194334**	IRAP-A	ENALOS	"inactive"	1.854	2.239	-4.763	"negative"
	**Z4251134045**	IRAP-A	ENALOS	"inactive"	0.899	2.200	-3.569	"negative"
	**Z4251281141**	IRAP-A	VISUAL	"inactive"	1.376	1.795	-4.109	"negative"
	**PV-003830110125**	IRAP-A	VISUAL	"inactive"	0.371	-0.144	-2.170	"negative"
	**PV-003616439313**	IRAP-A	VISUAL	"inactive"	0.286	-1.858	-1.365	"negative"
	**Z4251310526**	IRAP-A	VISUAL	"inactive"	1.130	1.424	-2.167	"negative"
	**Z4210354255**	IRAP-A	ENALOS	"inactive"	1.441	1.554	-3.559	"negative"
	**Z2898216118**	IRAP-A	VISUAL	"inactive"	0.411	-0.293	-2.190	"negative"
	**PV-005389180408**	IRAP-A	ENALOS	"inactive"	0.512	2.020	-2.874	"negative"
	**PV-003698827517**	IRAP-A	ENALOS	"inactive"	0.994	1.928	-1.809	"negative"
	**Z5341541952**	IRAP-A	ENALOS	"inactive"	0.518	2.336	-4.351	"negative"
	**Z4562164670**	IRAP-A	VISUAL	"inactive"	1.052	1.524	-2.443	"negative"
	**PV-002902365021**	IRAP-A	VISUAL	"inactive"	0.383	-0.879	-1.248	"positive"
	**PV-002711377975**	IRAP-A	VISUAL	"inactive"	2.091	1.716	-3.793	"negative"
	**Z4001682850**	IRAP-A	VISUAL	"inactive"	1.202	1.592	-2.327	"negative"
	**PV-005438447506**	IRAP-A	VISUAL	"inactive"	0.328	0.292	-3.108	"negative"

Ordered No.	Enamine ID	IRAP state	Selection	Cytotoxicity Classification	MSlogBCF	MSlogP	MSlogS	Mutagenicity Classification
	**PV-003095717604**	IRAP-A	ENALOS	"inactive"	1.321	1.523	-3.761	"negative"
	**Z3861465857**	IRAP-A	ENALOS	"inactive"	1.328	1.774	-4.394	"negative"
	**Z4387370311**	IRAP-A	VISUAL	"inactive"	1.186	0.841	-2.500	"positive"
	**Z4387368384**	IRAP-A	VISUAL	"inactive"	1.102	1.802	-3.512	"negative"
	**Z4251479028**	IRAP-A	ENALOS	"inactive"	0.934	2.107	-2.940	"negative"
	**Z3508272727**	IRAP-A	ENALOS	"inactive"	1.600	1.748	-3.773	"negative"
	**Z5220742121**	IRAP-A	VISUAL	"inactive"	0.841	0.779	-2.377	"negative"
	**PV-006862462825**	IRAP-A	VISUAL	"inactive"	1.408	1.683	-3.697	"negative"
	**Z4210353093**	IRAP-A	ENALOS	"inactive"	1.595	1.743	-3.427	"negative"
	**Z3510007969**	IRAP-A	ENALOS	"inactive"	1.344	2.195	-4.571	"negative"
	**PV-003860597259**	IRAP-A	VISUAL	"inactive"	0.338	-0.546	-2.135	"negative"
	**Z4250578603**	IRAP-A	ENALOS	"inactive"	0.888	1.800	-4.392	"negative"
	**PV-003014300839**	IRAP-A	ENALOS	"inactive"	1.345	1.524	-2.569	"negative"
	**Z5286457552**	IRAP-A	ENALOS	"inactive"	1.204	2.097	-2.393	"negative"
	**Z3510003749**	IRAP-A	ENALOS	"inactive"	1.343	2.555	-4.053	"negative"
	**PV-006260900716**	IRAP-B	ENALOS	"inactive"	1.764	2.809	-5.978	"negative"
	**Z4175111789**	IRAP-B	VISUAL	"inactive"	1.183	0.467	-5.107	"negative"
	**Z4250310204**	IRAP-B	ENALOS	"inactive"	0.818	3.137	-4.869	"negative"
	**PV-005636810118**	IRAP-B	ENALOS	"inactive"	0.892	1.851	-4.264	"negative"
	**Z5592194898**	IRAP-B	ENALOS	"inactive"	1.682	1.816	-4.574	"negative"
	**Z5074742728**	IRAP-B	VISUAL	"active"	0.982	1.482	-2.685	"negative"
	**PV-006283977818**	IRAP-B	VISUAL	"inactive"	0.579	-0.272	-2.152	"negative"
6	**Z4380815781**	IRAP-B	ENALOS	"inactive"	1.684	1.983	-4.318	"negative"
	**PV-003315399085**	IRAP-B	VISUAL	"inactive"	0.668	0.093	-3.318	"negative"
	**Z4001220567**	IRAP-B	ENALOS	"inactive"	1.253	1.189	-5.827	"negative"
	**Z3879747050**	IRAP-B	ENALOS	"inactive"	1.673	2.428	-3.462	"negative"

Ordered No.	Enamine ID	IRAP state	Selection	Cytotoxicity Classification	MSlogBCF	MSlogP	MSlogS	Mutagenicity Classification
	**PV-006005181456**	IRAP-B	VISUAL	"inactive"	0.842	0.307	-4.004	"negative"
	**PV-006383708231**	IRAP-B	ENALOS	"inactive"	0.718	2.441	-4.197	"negative"
	**PV-005433944246**	IRAP-B	VISUAL	"inactive"	1.641	1.599	-3.897	"negative"
	**PV-003186566901**	IRAP-B	VISUAL	"inactive"	0.828	0.291	-2.800	"negative"
7	**Z4001220365**	IRAP-B	ENALOS	"inactive"	1.564	1.952	-6.076	"negative"
	**PV-006884085939**	IRAP-B	VISUAL	"inactive"	1.258	1.465	-2.623	"negative"
	**Z4115949382**	IRAP-B	ENALOS	"inactive"	1.698	1.907	-3.872	"negative"
	**PV-006805391731**	IRAP-B	ENALOS	"inactive"	0.886	2.273	-3.078	"negative"
	**Z4081743610**	IRAP-B	VISUAL	"inactive"	0.944	-0.115	-3.895	"negative"
	**PV-005727181030**	IRAP-B	ENALOS	"inactive"	0.550	2.551	-3.710	"negative"
	**PV-002779753296**	IRAP-B	VISUAL	"inactive"	0.531	-0.278	-1.547	"negative"
	**PV-005970070526**	IRAP-B	ENALOS	"inactive"	1.443	1.619	-5.349	"negative"
	**PV-003062318855**	IRAP-B	VISUAL	"inactive"	0.384	-1.247	-1.925	"negative"
	**PV-003431445904**	IRAP-B	VISUAL	"inactive"	0.966	0.526	-3.235	"positive"
	**PV-004495093970**	IRAP-B	ENALOS	"inactive"	0.856	1.863	-3.947	"negative"
8	**Z2898226219**	IRAP-B	VISUAL	"inactive"	1.428	2.759	-3.389	"negative"
	**PV-006963367379**	IRAP-B	ENALOS	"inactive"	1.659	2.088	-3.264	"negative"
	**PV-007010261716**	IRAP-B	ENALOS	"inactive"	0.921	1.879	-2.046	"negative"
	**PV-005466392658**	IRAP-B	VISUAL	"inactive"	0.534	1.157	-3.819	"negative"
	**PV-003111086729**	IRAP-B	VISUAL	"inactive"	0.260	-1.201	-1.564	"positive"
	**PV-007037204777**	IRAP-B	VISUAL	"inactive"	1.391	1.264	-5.631	"positive"
	**PV-004482068029**	IRAP-B	VISUAL	"inactive"	0.480	0.437	-2.223	"positive"
	**PV-007017201417**	IRAP-B	ENALOS	"inactive"	1.489	2.541	-3.877	"negative"
	**Z3347802976**	IRAP-B	ENALOS	"inactive"	0.431	1.882	-1.948	"negative"
	**PV-005022891113**	IRAP-B	VISUAL	"inactive"	1.000	1.353	-4.216	"negative"
	**PV-004158159053**	IRAP-B	VISUAL	"inactive"	1.776	1.811	-5.625	"negative"

Ordered No.	Enamine ID	IRAP state	Selection	Cytotoxicity Classification	MSlogBCF	MSlogP	MSlogS	Mutagenicity Classification
	**PV-002939007821**	IRAP-B	VISUAL	"inactive"	1.006	1.582	-2.241	"negative"
	**PV-003467682774**	IRAP-B	VISUAL	"inactive"	0.569	0.404	-1.892	"positive"
	**Z4240409427**	IRAP-B	VISUAL	"inactive"	0.372	0.409	-1.470	"negative"
	**Z5394358312**	IRAP-B	ENALOS	"inactive"	1.922	2.599	-4.553	"negative"
	**PV-007043660508**	IRAP-B	VISUAL	"inactive"	0.527	-0.329	-1.816	"negative"
	**PV-006853346153**	IRAP-B	ENALOS	"inactive"	1.154	1.855	-5.017	"negative"
	**PV-005372643423**	IRAP-B	VISUAL	"inactive"	1.180	1.185	-5.574	"positive"
	**Z4250350170**	IRAP-B	VISUAL	"inactive"	0.323	-1.135	-1.108	"negative"
	**PV-006802194032**	IRAP-B	VISUAL	"inactive"	0.445	2.753	-3.675	"negative"
	**Z5292279984**	IRAP-B	VISUAL	"inactive"	0.548	1.103	-2.148	"negative"
	**PV-006781773614**	IRAP-B	ENALOS	"inactive"	1.599	1.874	-5.549	"negative"
	**Z4210353253**	IRAP-B	VISUAL	"inactive"	1.039	0.478	-1.966	"negative"
	**PV-004332340507**	IRAP-B	VISUAL	"inactive"	0.275	-1.262	-2.980	"positive"
	**PV-003405258552**	IRAP-B	VISUAL	"inactive"	0.735	0.457	-2.063	"negative"
	**PV-002887518540**	IRAP-B	VISUAL	"inactive"	0.385	0.229	-0.787	"negative"
	**Z4240408572**	IRAP-B	ENALOS	"inactive"	1.365	2.294	-3.851	"negative"
9	**PV-002947517349**	IRAP-B	VISUAL	"inactive"	0.503	0.037	-1.420	"negative"
	**Z3609506103**	IRAP-B	ENALOS	"inactive"	1.085	1.924	-3.742	"negative"
	**PV-007108701995**	IRAP-B	ENALOS	"inactive"	1.687	2.271	-4.945	"negative"
	**PV-006334475960**	IRAP-B	ENALOS	"inactive"	0.748	2.038	-3.897	"negative"
	**PV-005133931482**	IRAP-B	VISUAL	"inactive"	0.505	-0.413	-2.167	"negative"
	**PV-002908100897**	IRAP-B	VISUAL	"active"	1.404	1.133	-5.103	"negative"
	**PV-004160528578**	IRAP-B	VISUAL	"inactive"	0.840	1.538	-3.804	"negative"
	**PV-006943073003**	IRAP-B	ENALOS	"inactive"	1.327	1.547	-4.111	"negative"
	**PV-006744440553**	IRAP-B	ENALOS	"inactive"	0.895	1.637	-4.234	"negative"
	**PV-003208817841**	IRAP-B	VISUAL	"inactive"	0.384	0.431	-2.114	"negative"

Ordered No.	Enamine ID	IRAP state	Selection	Cytotoxicity Classification	MSlogBCF	MSlogP	MSlogS	Mutagenicity Classification
	**PV-006848007303**	IRAP-B	ENALOS	"inactive"	1.612	1.911	-3.732	"negative"
	**Z1951089022**	IRAP-B	ENALOS	"inactive"	1.404	2.458	-3.474	"negative"
	**Z4507718605**	IRAP-B	ENALOS	"inactive"	1.116	1.891	-3.190	"negative"
	**PV-006369094312**	IRAP-B	ENALOS	"inactive"	0.827	1.779	-4.294	"negative"
	**Z4507718605**	IRAP-B	ENALOS	"inactive"	1.116	1.891	-3.187	"negative"
	**PV-005497177697**	IRAP-O	ENALOS	"inactive"	0.835	1.693	-3.688	"negative"
	**Z4115312583**	IRAP-O	VISUAL	"inactive"	0.515	0.199	-3.067	"negative"
10	**Z3386081457**	IRAP-O	ENALOS	"inactive"	0.902	1.767	-1.772	"negative"
11	**PV-003267964142**	IRAP-O	VISUAL	"inactive"	1.835	0.862	-4.575	"negative"
12	**Z4309616340**	IRAP-O	ENALOS	"inactive"	1.197	1.901	-5.124	"negative"
13	**Z3385671698**	IRAP-O	ENALOS	"inactive"	1.384	2.136	-5.382	"negative"
	**PV-005804701749**	IRAP-O	ENALOS	"inactive"	0.904	2.037	-3.969	"negative"
14	**Z5518863768**	IRAP-O	ENALOS	"inactive"	1.561	2.503	-6.020	"negative"
	**Z5282597398**	IRAP-O	VISUAL	"inactive"	0.427	-0.036	-1.537	"negative"
	**Z4272410596**	IRAP-O	VISUAL	"inactive"	1.439	1.428	-4.943	"negative"
	**PV-005690321219**	IRAP-O	ENALOS	"inactive"	2.006	1.930	-4.577	"negative"
15	**Z4601470241**	IRAP-O	ENALOS	"inactive"	0.510	2.442	-4.288	"negative"
	**PV-003992050528**	IRAP-O	VISUAL	"inactive"	1.737	0.435	-4.907	"negative"
	**PV-004560032796**	IRAP-O	ENALOS	"inactive"	0.875	1.959	-4.533	"negative"
	**PV-005668578965**	IRAP-O	VISUAL	"inactive"	0.901	1.246	-2.185	"positive"
	**PV-005422025641**	IRAP-O	ENALOS	"inactive"	0.833	2.609	-4.003	"negative"
16	**PV-002764876330**	IRAP-O	VISUAL	"inactive"	0.369	-0.550	-1.556	"positive"
	**PV-006855251261**	IRAP-O	VISUAL	"inactive"	0.352	0.140	-2.732	"positive"
	**Z4309751849**	IRAP-O	VISUAL	"inactive"	0.877	1.574	-4.334	"negative"
	**PV-004651371208**	IRAP-O	VISUAL	"inactive"	1.334	1.191	-4.831	"negative"
17	**PV-006830307217**	IRAP-O	VISUAL	"active"	1.729	1.453	-5.692	"negative"

Ordered No.	Enamine ID	IRAP state	Selection	Cytotoxicity Classification	MSlogBCF	MSlogP	MSlogS	Mutagenicity Classification
	**PV-006906684662**	IRAP-O	VISUAL	"inactive"	0.345	-0.301	-1.700	"positive"
	**PV-005097643364**	IRAP-O	VISUAL	"inactive"	0.840	0.569	-3.786	"negative"
	**PV-006074765607**	IRAP-O	ENALOS	"inactive"	0.853	2.292	-3.955	"negative"
	**Z4272410601**	IRAP-O	ENALOS	"inactive"	1.261	1.764	-4.889	"negative"
	**PV-004990993519**	IRAP-O	ENALOS	"inactive"	1.186	1.908	-4.594	"negative"
	**PV-003666315616**	IRAP-O	VISUAL	"inactive"	1.525	1.298	-4.357	"negative"
	**PV-006376531261**	IRAP-O	VISUAL	"inactive"	0.998	1.515	-4.278	"negative"
	**PV-005804701749**	IRAP-O	VISUAL	"inactive"	0.904	2.037	-3.968	"negative"
	**PV-004452500357**	IRAP-O	VISUAL	"inactive"	0.525	0.456	-2.193	"negative"
	**PV-007050107558**	IRAP-O	VISUAL	"inactive"	1.026	2.223	-4.691	"negative"
	**Z4472167234**	IRAP-O	ENALOS	"inactive"	1.299	1.668	-3.771	"negative"
	**PV-006828053122**	IRAP-O	VISUAL	"inactive"	0.927	0.576	-3.842	"negative"
	**Z4312297770**	IRAP-O	ENALOS	"inactive"	1.873	3.374	-5.102	"negative"
	**PV-005717770058**	IRAP-O	VISUAL	"inactive"	0.373	1.116	-2.437	"negative"
	**PV-006337896860**	IRAP-O	VISUAL	"inactive"	1.256	1.864	-5.065	"negative"
	**PV-004762123740**	IRAP-O	VISUAL	"inactive"	0.932	1.452	-5.157	"negative"
	**PV-004393531270**	IRAP-O	ENALOS	"inactive"	1.671	1.771	-5.267	"negative"
	**PV-004233542396**	IRAP-O	ENALOS	"inactive"	0.361	1.737	-4.041	"negative"
	**PV-007117827737**	IRAP-O	VISUAL	"inactive"	1.298	1.492	-5.097	"positive"
	**PV-006555934998**	IRAP-O	VISUAL	"inactive"	1.396	0.482	-4.724	"negative"
	**PV-002853724860**	IRAP-O	VISUAL	"inactive"	0.807	1.252	-3.925	"negative"
	**PV-004350213648**	IRAP-O	VISUAL	"inactive"	0.522	0.416	-1.431	"negative"
	**Z4225996356**	IRAP-O	ENALOS	"inactive"	0.883	1.991	-1.575	"negative"
	**PV-003325676028**	IRAP-O	VISUAL	"inactive"	1.655	2.614	-5.066	"negative"
	**PV-003036335116**	IRAP-O	VISUAL	"inactive"	0.948	1.265	-3.758	"positive"
	**PV-006897939853**	IRAP-O	VISUAL	"inactive"	0.355	0.019	-2.381	"positive"

Ordered No.	Enamine ID	IRAP state	Selection	Cytotoxicity Classification	MSlogBCF	MSlogP	MSlogS	Mutagenicity Classification
	**Z4879764433**	IRAP-O	VISUAL	"inactive"	0.518	0.094	-3.454	"positive"
	**PV-004595861284**	IRAP-O	VISUAL	"inactive"	0.919	0.290	-4.304	"negative"
	**Z4828721937**	IRAP-O	ENALOS	"inactive"	1.048	1.631	-2.945	"negative"
	**PV-006677779485**	IRAP-O	ENALOS	"inactive"	0.802	2.051	-3.875	"negative"
	**PV-004715269800**	IRAP-O	ENALOS	"inactive"	1.208	2.340	-4.865	"negative"
	**Z4594993615**	IRAP-O	ENALOS	"inactive"	0.821	2.248	-3.710	"negative"
	**PV-006229431204**	IRAP-O	ENALOS	"inactive"	0.735	2.504	-4.321	"negative"
	**PV-006956939305**	IRAP-O	ENALOS	"inactive"	0.526	1.677	-3.225	"negative"
	**PV-006845993993**	IRAP-O	VISUAL	"active"	0.939	0.774	-3.564	"negative"
	**Z4154684703**	IRAP-O	ENALOS	"inactive"	0.889	2.880	-3.497	"negative"
	**PV-003015074737**	IRAP-O	ENALOS	"inactive"	1.247	2.332	-4.533	"negative"
	**PV-004476105682**	IRAP-O	ENALOS	"inactive"	0.826	1.528	-3.781	"negative"
	**Z3413964093**	IRAP-O	VISUAL	"inactive"	0.836	-0.452	-3.408	"positive"

Supplemental Figure S2: Detailed physiochemical properties and pharmacokinetic predictions computed using SwissADME for the 17 selected compounds**.**

Molecule 1

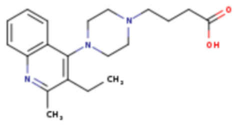

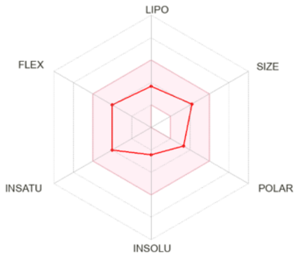

SMILES CCc1c(C)nc2c(c1N1CCN(CC1)CCCC(=O)O)cccc2

Physicochemical Properties	
Formula	C20H27N3O2
Molecular weight	341.45 g/mol
Num. heavy atoms	25
Num. arom. heavy atoms	10
Fraction Csp3	0.50
Num. rotatable bonds	6
Num. H-bond acceptors	4
Num. H-bond donors	1
Molar Refractivity	108.93
TPSA	56.67 Å²

Lipophilicity	
Log $P_{o/w}$ (iLOGP)	3.11
Log $P_{o/w}$ (XLOGP3)	0.90
Log $P_{o/w}$ (WLOGP)	2.33
Log $P_{o/w}$ (MLOGP)	2.21
Log $P_{o/w}$ (SILICOS-IT)	3.39
Consensus Log $P_{o/w}$	2.39

Water Solubility	
Log *S* (ESOL)	-2.42
Solubility	1.29e+00 mg/ml ; 3.77e-03 mol/l
Class	Soluble
Log *S* (Ali)	-1.68
Solubility	7.21e+00 mg/ml ; 2.11e-02 mol/l
Class	Very soluble
Log *S* (SILICOS-IT)	-5.22
Solubility	2.08e-03 mg/ml ; 6.08e-06 mol/l
Class	Moderately soluble

Pharmacokinetics	
GI absorption	High
BBB permeant	Yes
P-gp substrate	Yes
CYP1A2 inhibitor	No
CYP2C19 inhibitor	No
CYP2C9 inhibitor	No
CYP2D6 inhibitor	Yes
CYP3A4 inhibitor	No
Log K_p (skin permeation)	-7.74 cm/s

Druglikeness	
Lipinski	Yes; 0 violation
Ghose	Yes
Veber	Yes
Egan	Yes
Muegge	Yes
Bioavailability Score	0.55

Medicinal Chemistry	
PAINS	0 alert
Brenk	0 alert
Leadlikeness	Yes
Synthetic accessibility	2.63

Molecule 2

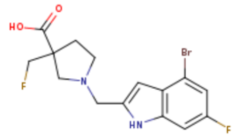

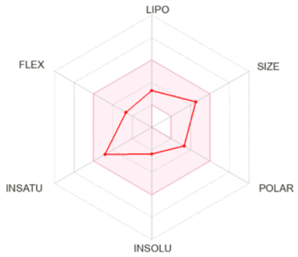

SMILES FCC1(CCN(C1)Cc1cc2c([nH]1)cc(cc2Br)F)C(=O)O

Physicochemical Properties	
Formula	C15H15BrF2N2O2
Molecular weight	373.19 g/mol
Num. heavy atoms	22
Num. arom. heavy atoms	9
Fraction Csp3	0.40
Num. rotatable bonds	4
Num. H-bond acceptors	5
Num. H-bond donors	2
Molar Refractivity	86.02
TPSA	56.33 Å²

Lipophilicity	
Log $P_{o/w}$ (iLOGP)	2.30
Log $P_{o/w}$ (XLOGP3)	0.26
Log $P_{o/w}$ (WLOGP)	3.62
Log $P_{o/w}$ (MLOGP)	2.77
Log $P_{o/w}$ (SILICOS-IT)	4.03
Consensus Log $P_{o/w}$	2.60

Water Solubility	
Log *S* (ESOL)	-2.36
Solubility	1.64e+00 mg/ml ; 4.40e-03 mol/l
Class	Soluble
Log *S* (Ali)	-1.00
Solubility	3.70e+01 mg/ml ; 9.91e-02 mol/l
Class	Very soluble
Log *S* (SILICOS-IT)	-5.25
Solubility	2.07e-03 mg/ml ; 5.56e-06 mol/l
Class	Moderately soluble

Pharmacokinetics	
GI absorption	High
BBB permeant	Yes
P-gp substrate	Yes
CYP1A2 inhibitor	No
CYP2C19 inhibitor	No
CYP2C9 inhibitor	No
CYP2D6 inhibitor	Yes
CYP3A4 inhibitor	No
Log K_p (skin permeation)	-8.39 cm/s

Druglikeness	
Lipinski	Yes; 0 violation
Ghose	Yes
Veber	Yes
Egan	Yes
Muegge	Yes
Bioavailability Score	0.55

Medicinal Chemistry	
PAINS	0 alert
Brenk	0 alert
Leadlikeness	No; 1 violation: MW>350
Synthetic accessibility	2.99

Molecule 3

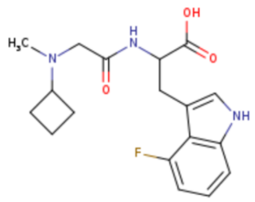

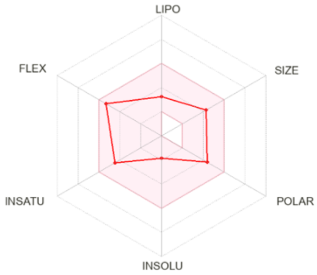

SMILES O=C(CN(C1CCC1)C)NC(C(=O)O)Cc1c[nH]c2c1c(F)ccc2

Physicochemical Properties	
Formula	C18H22FN3O3
Molecular weight	347.38 g/mol
Num. heavy atoms	25
Num. arom. heavy atoms	9
Fraction Csp3	0.44
Num. rotatable bonds	8
Num. H-bond acceptors	5
Num. H-bond donors	3
Molar Refractivity	92.04
TPSA	85.43 Å²

Lipophilicity	
Log $P_{o/w}$ (iLOGP)	0.00
Log $P_{o/w}$ (XLOGP3)	0.17
Log $P_{o/w}$ (WLOGP)	2.32
Log $P_{o/w}$ (MLOGP)	1.42
Log $P_{o/w}$ (SILICOS-IT)	1.48
Consensus Log $P_{o/w}$	1.08

Water Solubility	
Log *S* (ESOL)	-1.84
Solubility	5.03e+00 mg/ml ; 1.45e-02 mol/l
Class	Very soluble
Log *S* (Ali)	-1.52
Solubility	1.05e+01 mg/ml ; 3.01e-02 mol/l
Class	Very soluble
Log *S* (SILICOS-IT)	-2.30
Solubility	1.74e+00 mg/ml ; 5.00e-03 mol/l
Class	Soluble

Pharmacokinetics	
GI absorption	High
BBB permeant	No
P-gp substrate	Yes
CYP1A2 inhibitor	No
CYP2C19 inhibitor	No
CYP2C9 inhibitor	No
CYP2D6 inhibitor	No
CYP3A4 inhibitor	No
Log K_p (skin permeation)	-8.30 cm/s

Druglikeness	
Lipinski	Yes; 0 violation
Ghose	Yes
Veber	Yes
Egan	Yes
Muegge	Yes
Bioavailability Score	0.55

Medicinal Chemistry	
PAINS	0 alert
Brenk	0 alert
Leadlikeness	No; 1 violation: Rotors>7
Synthetic accessibility	3.23

Molecule 4

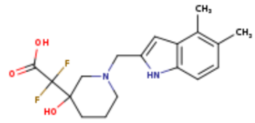

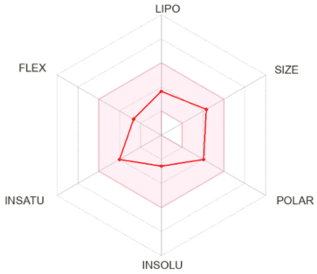

SMILES OC(=O)C(C1(O)CCCN(C1)Cc1cc2c([nH]1)ccc(c2C)C)(F)F

Physicochemical Properties	
Formula	C18H22F2N2O3
Molecular weight	352.38 g/mol
Num. heavy atoms	25
Num. arom. heavy atoms	9
Fraction Csp3	0.50
Num. rotatable bonds	4
Num. H-bond acceptors	6
Num. H-bond donors	3
Molar Refractivity	94.65
TPSA	76.56 Å²

Lipophilicity	
Log $P_{o/w}$ (iLOGP)	1.76
Log $P_{o/w}$ (XLOGP3)	0.85
Log $P_{o/w}$ (WLOGP)	3.14
Log $P_{o/w}$ (MLOGP)	1.76
Log $P_{o/w}$ (SILICOS-IT)	3.56
Consensus Log $P_{o/w}$	2.21

Water Solubility	
Log *S* (ESOL)	-2.56
Solubility	9.65e-01 mg/ml ; 2.74e-03 mol/l
Class	Soluble
Log *S* (Ali)	-2.04
Solubility	3.21e+00 mg/ml ; 9.10e-03 mol/l
Class	Soluble
Log *S* (SILICOS-IT)	-4.54
Solubility	1.01e-02 mg/ml ; 2.88e-05 mol/l
Class	Moderately soluble

Pharmacokinetics	
GI absorption	High
BBB permeant	Yes
P-gp substrate	Yes
CYP1A2 inhibitor	No
CYP2C19 inhibitor	No
CYP2C9 inhibitor	No
CYP2D6 inhibitor	Yes
CYP3A4 inhibitor	No
Log K_p (skin permeation)	-7.85 cm/s

Druglikeness	
Lipinski	Yes; 0 violation
Ghose	Yes
Veber	Yes
Egan	Yes
Muegge	Yes
Bioavailability Score	0.55

Medicinal Chemistry	
PAINS	0 alert
Brenk	0 alert
Leadlikeness	No; 1 violation: MW>350
Synthetic accessibility	3.08

Molecule 5

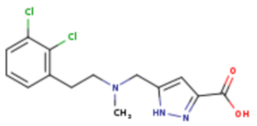

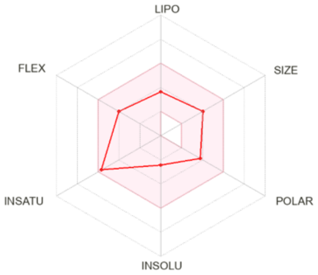

SMILES CN(Cc1[nH]nc(c1)C(=O)O)CCc1cccc(c1Cl)Cl

Physicochemical Properties	
Formula	C14H15Cl2N3O2
Molecular weight	328.19 g/mol
Num. heavy atoms	21
Num. arom. heavy atoms	11
Fraction Csp3	0.29
Num. rotatable bonds	6
Num. H-bond acceptors	4
Num. H-bond donors	2
Molar Refractivity	82.34
TPSA	69.22 Å²

Lipophilicity	
Log $P_{o/w}$ (iLOGP)	2.07
Log $P_{o/w}$ (XLOGP3)	0.88
Log $P_{o/w}$ (WLOGP)	2.94
Log $P_{o/w}$ (MLOGP)	2.35
Log $P_{o/w}$ (SILICOS-IT)	3.40
Consensus Log $P_{o/w}$	2.33

Water Solubility	
Log *S* (ESOL)	-2.42
Solubility	1.25e+00 mg/ml ; 3.79e-03 mol/l
Class	Soluble
Log *S* (Ali)	-1.92
Solubility	3.96e+00 mg/ml ; 1.21e-02 mol/l
Class	Very soluble
Log *S* (SILICOS-IT)	-5.27
Solubility	1.76e-03 mg/ml ; 5.36e-06 mol/l
Class	Moderately soluble

Pharmacokinetics	
GI absorption	High
BBB permeant	Yes
P-gp substrate	Yes
CYP1A2 inhibitor	No
CYP2C19 inhibitor	No
CYP2C9 inhibitor	No
CYP2D6 inhibitor	No
CYP3A4 inhibitor	No
Log K_p (skin permeation)	-7.68 cm/s

Druglikeness	
Lipinski	Yes; 0 violation
Ghose	Yes
Veber	Yes
Egan	Yes
Muegge	Yes
Bioavailability Score	0.55

Medicinal Chemistry	
PAINS	0 alert
Brenk	0 alert
Leadlikeness	Yes
Synthetic accessibility	2.36

Molecule 6

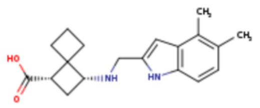

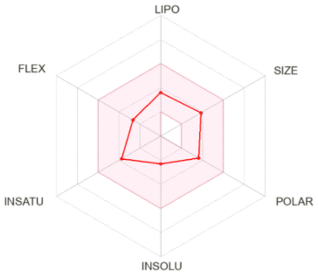

SMILES OC(=O)[C@H]1C[C@H](C21CCC2)NCc1cc2c([nH]1)ccc(c2C)C

Physicochemical Properties	
Formula	C19H24N2O2
Molecular weight	312.41 g/mol
Num. heavy atoms	23
Num. arom. heavy atoms	9
Fraction Csp3	0.53
Num. rotatable bonds	4
Num. H-bond acceptors	3
Num. H-bond donors	3
Molar Refractivity	91.74
TPSA	65.12 Å²

Lipophilicity	
Log $P_{o/w}$ (iLOGP)	2.69
Log $P_{o/w}$ (XLOGP3)	0.78
Log $P_{o/w}$ (WLOGP)	3.37
Log $P_{o/w}$ (MLOGP)	2.59
Log $P_{o/w}$ (SILICOS-IT)	3.98
Consensus Log $P_{o/w}$	2.68

Water Solubility	
Log *S* (ESOL)	-2.29
Solubility	1.59e+00 mg/ml ; 5.08e-03 mol/l
Class	Soluble
Log *S* (Ali)	-1.73
Solubility	5.84e+00 mg/ml ; 1.87e-02 mol/l
Class	Very soluble
Log *S* (SILICOS-IT)	-5.34
Solubility	1.43e-03 mg/ml ; 4.59e-06 mol/l
Class	Moderately soluble

Pharmacokinetics	
GI absorption	High
BBB permeant	Yes
P-gp substrate	Yes
CYP1A2 inhibitor	No
CYP2C19 inhibitor	No
CYP2C9 inhibitor	No
CYP2D6 inhibitor	Yes
CYP3A4 inhibitor	No
Log K_p (skin permeation)	-7.65 cm/s

Druglikeness	
Lipinski	Yes; 0 violation
Ghose	Yes
Veber	Yes
Egan	Yes
Muegge	Yes
Bioavailability Score	0.55

Medicinal Chemistry	
PAINS	0 alert
Brenk	0 alert
Leadlikeness	Yes
Synthetic accessibility	3.56

Molecule 7

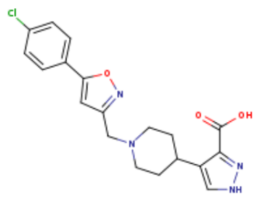

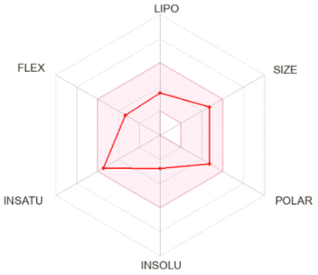

SMILES Clc1ccc(cc1)c1onc(c1)CN1CCC(CC1)c1c[nH]nc1C(=O)O

Physicochemical Properties	
Formula	C19H19ClN4O3
Molecular weight	386.83 g/mol
Num. heavy atoms	27
Num. arom. heavy atoms	16
Fraction Csp3	0.32
Num. rotatable bonds	5
Num. H-bond acceptors	6
Num. H-bond donors	2
Molar Refractivity	104.24
TPSA	95.25 Å²

Lipophilicity	
Log $P_{o/w}$ (iLOGP)	1.87
Log $P_{o/w}$ (XLOGP3)	0.64
Log $P_{o/w}$ (WLOGP)	3.26
Log $P_{o/w}$ (MLOGP)	2.03
Log $P_{o/w}$ (SILICOS-IT)	3.42
Consensus Log $P_{o/w}$	2.24

Water Solubility	
Log *S* (ESOL)	-2.75
Solubility	6.88e-01 mg/ml ; 1.78e-03 mol/l
Class	Soluble
Log *S* (Ali)	-2.22
Solubility	2.35e+00 mg/ml ; 6.09e-03 mol/l
Class	Soluble
Log *S* (SILICOS-IT)	-5.93
Solubility	4.52e-04 mg/ml ; 1.17e-06 mol/l
Class	Moderately soluble

Pharmacokinetics	
GI absorption	High
BBB permeant	No
P-gp substrate	Yes
CYP1A2 inhibitor	No
CYP2C19 inhibitor	No
CYP2C9 inhibitor	No
CYP2D6 inhibitor	No
CYP3A4 inhibitor	No
Log K_p (skin permeation)	-8.21 cm/s

Druglikeness	
Lipinski	Yes; 0 violation
Ghose	Yes
Veber	Yes
Egan	Yes
Muegge	Yes
Bioavailability Score	0.55

Medicinal Chemistry	
PAINS	0 alert
Brenk	0 alert
Leadlikeness	No; 1 violation: MW>350
Synthetic accessibility	3.18

Molecule 8

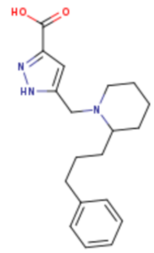

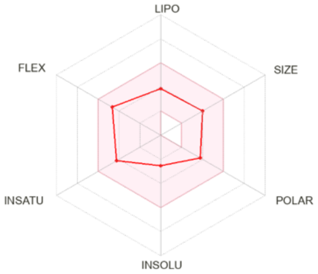

SMILES OC(=O)c1n[nH]c(c1)CN1CCCCC1CCCc1ccccc1

Physicochemical Properties	
Formula	C19H25N3O2
Molecular weight	327.42 g/mol
Num. heavy atoms	24
Num. arom. heavy atoms	11
Fraction Csp3	0.47
Num. rotatable bonds	7
Num. H-bond acceptors	4
Num. H-bond donors	2
Molar Refractivity	98.15
TPSA	69.22 Å²

Lipophilicity	
Log $P_{o/w}$ (iLOGP)	2.49
Log $P_{o/w}$ (XLOGP3)	1.25
Log $P_{o/w}$ (WLOGP)	2.95
Log $P_{o/w}$ (MLOGP)	2.53
Log $P_{o/w}$ (SILICOS-IT)	3.54
Consensus Log $P_{o/w}$	2.55

Water Solubility	
Log *S* (ESOL)	-2.53
Solubility	9.56e-01 mg/ml ; 2.92e-03 mol/l
Class	Soluble
Log *S* (Ali)	-2.30
Solubility	1.63e+00 mg/ml ; 4.99e-03 mol/l
Class	Soluble
Log *S* (SILICOS-IT)	-5.21
Solubility	2.00e-03 mg/ml ; 6.11e-06 mol/l
Class	Moderately soluble

Pharmacokinetics	
GI absorption	High
BBB permeant	Yes
P-gp substrate	Yes
CYP1A2 inhibitor	No
CYP2C19 inhibitor	No
CYP2C9 inhibitor	No
CYP2D6 inhibitor	Yes
CYP3A4 inhibitor	No
Log K_p (skin permeation)	-7.41 cm/s

Druglikeness	
Lipinski	Yes; 0 violation
Ghose	Yes
Veber	Yes
Egan	Yes
Muegge	Yes
Bioavailability Score	0.55

Medicinal Chemistry	
PAINS	0 alert
Brenk	0 alert
Leadlikeness	Yes
Synthetic accessibility	3.10

Molecule 9

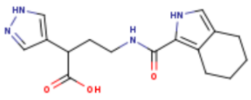

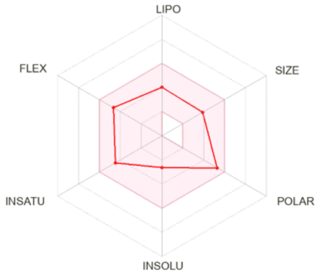

SMILES OC(=O)C(c1c[nH]nc1)CCNC(=O)c1[nH]cc2c1CCCC2

Physicochemical Properties	
Formula	C16H20N4O3
Molecular weight	316.36 g/mol
Num. heavy atoms	23
Num. arom. heavy atoms	10
Fraction Csp3	0.44
Num. rotatable bonds	7
Num. H-bond acceptors	4
Num. H-bond donors	4
Molar Refractivity	84.05
TPSA	110.87 Å²

Lipophilicity	
Log $P_{o/w}$ (iLOGP)	0.76
Log $P_{o/w}$ (XLOGP3)	1.54
Log $P_{o/w}$ (WLOGP)	1.60
Log $P_{o/w}$ (MLOGP)	0.57
Log $P_{o/w}$ (SILICOS-IT)	2.72
Consensus Log $P_{o/w}$	1.44

Water Solubility	
Log *S* (ESOL)	-2.63
Solubility	7.39e-01 mg/ml ; 2.34e-03 mol/l
Class	Soluble
Log *S* (Ali)	-3.48
Solubility	1.05e-01 mg/ml ; 3.33e-04 mol/l
Class	Soluble
Log *S* (SILICOS-IT)	-4.29
Solubility	1.64e-02 mg/ml ; 5.19e-05 mol/l
Class	Moderately soluble

Pharmacokinetics	
GI absorption	High
BBB permeant	No
P-gp substrate	Yes
CYP1A2 inhibitor	No
CYP2C19 inhibitor	No
CYP2C9 inhibitor	No
CYP2D6 inhibitor	No
CYP3A4 inhibitor	No
Log K_p (skin permeation)	-7.14 cm/s

Druglikeness	
Lipinski	Yes; 0 violation
Ghose	Yes
Veber	Yes
Egan	Yes
Muegge	Yes
Bioavailability Score	0.56

Medicinal Chemistry	
PAINS	0 alert
Brenk	0 alert
Leadlikeness	Yes
Synthetic accessibility	3.15

Molecule 10

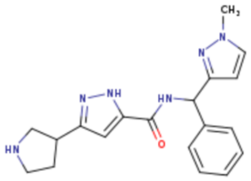

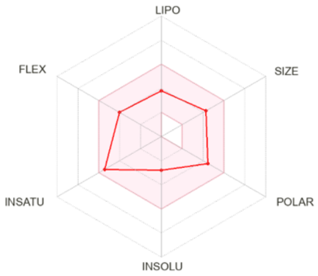

SMILES Cn1ccc(n1)C(c1ccccc1)NC(=O)c1[nH]nc(c1)C1CCNC1

Physicochemical Properties	
Formula	C19H22N6O
Molecular weight	350.42 g/mol
Num. heavy atoms	26
Num. arom. heavy atoms	16
Fraction Csp3	0.32
Num. rotatable bonds	6
Num. H-bond acceptors	4
Num. H-bond donors	3
Molar Refractivity	101.59
TPSA	87.63 Å²

Lipophilicity	
Log $P_{o/w}$ (iLOGP)	2.24
Log $P_{o/w}$ (XLOGP3)	1.15
Log $P_{o/w}$ (WLOGP)	1.03
Log $P_{o/w}$ (MLOGP)	1.13
Log $P_{o/w}$ (SILICOS-IT)	2.12
Consensus Log $P_{o/w}$	1.54

Water Solubility	
Log *S* (ESOL)	-2.80
Solubility	5.60e-01 mg/ml ; 1.60e-03 mol/l
Class	Soluble
Log *S* (Ali)	-2.58
Solubility	9.12e-01 mg/ml ; 2.60e-03 mol/l
Class	Soluble
Log *S* (SILICOS-IT)	-5.61
Solubility	8.62e-04 mg/ml ; 2.46e-06 mol/l
Class	Moderately soluble

Pharmacokinetics	
GI absorption	High
BBB permeant	No
P-gp substrate	Yes
CYP1A2 inhibitor	No
CYP2C19 inhibitor	Yes
CYP2C9 inhibitor	No
CYP2D6 inhibitor	Yes
CYP3A4 inhibitor	Yes
Log K_p (skin permeation)	-7.62 cm/s

Druglikeness	
Lipinski	Yes; 0 violation
Ghose	Yes
Veber	Yes
Egan	Yes
Muegge	Yes
Bioavailability Score	0.55

Medicinal Chemistry	
PAINS	0 alert
Brenk	0 alert
Leadlikeness	No; 1 violation: MW>350
Synthetic accessibility	3.69

Molecule 11

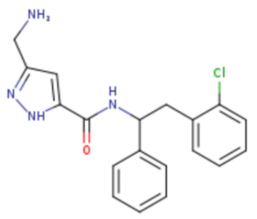

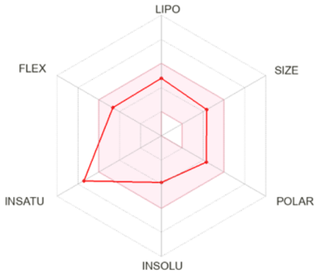

SMILES NCc1n[nH]c(c1)C(=O)NC(c1ccccc1)Cc1ccccc1Cl

Physicochemical Properties	
Formula	C19H19ClN4O
Molecular weight	354.83 g/mol
Num. heavy atoms	25
Num. arom. heavy atoms	17
Fraction Csp3	0.16
Num. rotatable bonds	7
Num. H-bond acceptors	3
Num. H-bond donors	3
Molar Refractivity	98.05
TPSA	83.80 Å²

Lipophilicity	
Log $P_{o/w}$ (iLOGP)	1.91
Log $P_{o/w}$ (XLOGP3)	2.83
Log $P_{o/w}$ (WLOGP)	2.76
Log $P_{o/w}$ (MLOGP)	2.39
Log $P_{o/w}$ (SILICOS-IT)	3.90
Consensus Log $P_{o/w}$	2.76

Water Solubility	
Log S (ESOL)	-3.86
Solubility	4.85e-02 mg/ml ; 1.37e-04 mol/l
Class	Soluble
Log S (Ali)	-4.25
Solubility	2.01e-02 mg/ml ; 5.65e-05 mol/l
Class	Moderately soluble
Log S (SILICOS-IT)	-7.34
Solubility	1.61e-05 mg/ml ; 4.55e-08 mol/l
Class	Poorly soluble

Pharmacokinetics	
GI absorption	High
BBB permeant	No
P-gp substrate	Yes
CYP1A2 inhibitor	No
CYP2C19 inhibitor	Yes
CYP2C9 inhibitor	No
CYP2D6 inhibitor	Yes
CYP3A4 inhibitor	Yes
Log K_p (skin permeation)	-6.46 cm/s

Druglikeness	
Lipinski	Yes; 0 violation
Ghose	Yes
Veber	Yes
Egan	Yes
Muegge	Yes
Bioavailability Score	0.55

Medicinal Chemistry	
PAINS	0 alert
Brenk	0 alert
Leadlikeness	No; 1 violation: MW>350
Synthetic accessibility	3.01

Molecule 12

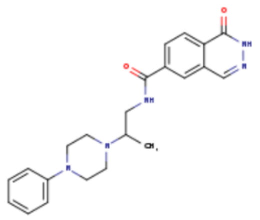

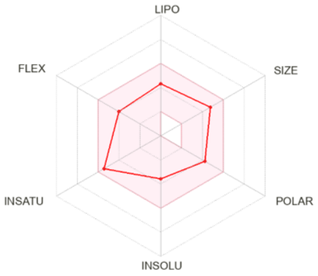

SMILES CC(N1CCN(CC1)c1ccccc1)CNC(=O)c1ccc2c(c1)cn[nH]c2=O

Physicochemical Properties	
Formula	C22H25N5O2
Molecular weight	391.47 g/mol
Num. heavy atoms	29
Num. arom. heavy atoms	16
Fraction Csp3	0.32
Num. rotatable bonds	6
Num. H-bond acceptors	4
Num. H-bond donors	2
Molar Refractivity	120.75
TPSA	81.33 Å²

Lipophilicity	
Log $P_{o/w}$ (iLOGP)	2.76
Log $P_{o/w}$ (XLOGP3)	2.05
Log $P_{o/w}$ (WLOGP)	1.10
Log $P_{o/w}$ (MLOGP)	2.23
Log $P_{o/w}$ (SILICOS-IT)	2.66
Consensus Log $P_{o/w}$	2.16

Water Solubility	
Log S (ESOL)	-3.57
Solubility	1.05e-01 mg/ml ; 2.69e-04 mol/l
Class	Soluble
Log S (Ali)	-3.39
Solubility	1.61e-01 mg/ml ; 4.11e-04 mol/l
Class	Soluble
Log S (SILICOS-IT)	-6.30
Solubility	1.94e-04 mg/ml ; 4.97e-07 mol/l
Class	Poorly soluble

Pharmacokinetics	
GI absorption	High
BBB permeant	No
P-gp substrate	Yes
CYP1A2 inhibitor	No
CYP2C19 inhibitor	No
CYP2C9 inhibitor	Yes
CYP2D6 inhibitor	Yes
CYP3A4 inhibitor	Yes
Log K_p (skin permeation)	-7.23 cm/s

Druglikeness	
Lipinski	Yes; 0 violation
Ghose	Yes
Veber	Yes
Egan	Yes
Muegge	Yes
Bioavailability Score	0.55

Medicinal Chemistry	
PAINS	0 alert
Brenk	0 alert
Leadlikeness	No; 1 violation: MW>350
Synthetic accessibility	3.11

Molecule 13

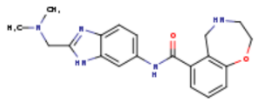

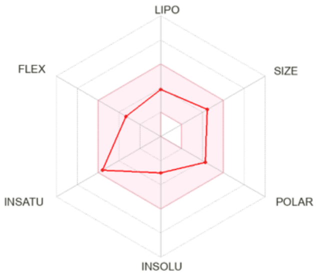

SMILES CN(Cc1nc2c([nH]1)cc(cc2)NC(=O)c1cccc2c1CNCCO2)C

Physicochemical Properties	
Formula	C20H23N5O2
Molecular weight	365.43 g/mol
Num. heavy atoms	27
Num. arom. heavy atoms	15
Fraction Csp3	0.30
Num. rotatable bonds	5
Num. H-bond acceptors	5
Num. H-bond donors	3
Molar Refractivity	108.65
TPSA	82.28 Å²

Lipophilicity	
Log $P_{o/w}$ (iLOGP)	2.26
Log $P_{o/w}$ (XLOGP3)	1.31
Log $P_{o/w}$ (WLOGP)	1.48
Log $P_{o/w}$ (MLOGP)	1.21
Log $P_{o/w}$ (SILICOS-IT)	2.66
Consensus Log $P_{o/w}$	1.79

Water Solubility	
Log *S* (ESOL)	-3.01
Solubility	3.55e-01 mg/ml ; 9.73e-04 mol/l
Class	Soluble
Log *S* (Ali)	-2.64
Solubility	8.40e-01 mg/ml ; 2.30e-03 mol/l
Class	Soluble
Log *S* (SILICOS-IT)	-6.52
Solubility	1.11e-04 mg/ml ; 3.05e-07 mol/l
Class	Poorly soluble

Pharmacokinetics	
GI absorption	High
BBB permeant	No
P-gp substrate	Yes
CYP1A2 inhibitor	Yes
CYP2C19 inhibitor	No
CYP2C9 inhibitor	No
CYP2D6 inhibitor	Yes
CYP3A4 inhibitor	Yes
Log K_p (skin permeation)	-7.60 cm/s

Druglikeness	
Lipinski	Yes; 0 violation
Ghose	Yes
Veber	Yes
Egan	Yes
Muegge	Yes
Bioavailability Score	0.55

Medicinal Chemistry	
PAINS	0 alert
Brenk	0 alert
Leadlikeness	No; 1 violation: MW>350
Synthetic accessibility	3.07

Molecule 14

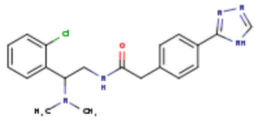

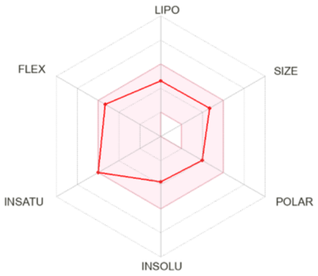

SMILES O=C(Cc1ccc(cc1)c1nnc[nH]1)NCC(c1ccccc1Cl)N(C)C

Physicochemical Properties	
Formula	C20H22ClN5O
Molecular weight	383.87 g/mol
Num. heavy atoms	27
Num. arom. heavy atoms	17
Fraction Csp3	0.25
Num. rotatable bonds	8
Num. H-bond acceptors	4
Num. H-bond donors	2
Molar Refractivity	106.21
TPSA	73.91 Å²

Lipophilicity	
Log $P_{o/w}$ (iLOGP)	2.40
Log $P_{o/w}$ (XLOGP3)	2.54
Log $P_{o/w}$ (WLOGP)	2.76
Log $P_{o/w}$ (MLOGP)	2.61
Log $P_{o/w}$ (SILICOS-IT)	3.74
Consensus Log $P_{o/w}$	2.81

Water Solubility	
Log *S* (ESOL)	-3.76
Solubility	6.70e-02 mg/ml ; 1.75e-04 mol/l
Class	Soluble
Log *S* (Ali)	-3.74
Solubility	7.00e-02 mg/ml ; 1.82e-04 mol/l
Class	Soluble
Log *S* (SILICOS-IT)	-7.42
Solubility	1.47e-05 mg/ml ; 3.82e-08 mol/l
Class	Poorly soluble

Pharmacokinetics	
GI absorption	High
BBB permeant	Yes
P-gp substrate	Yes
CYP1A2 inhibitor	No
CYP2C19 inhibitor	Yes
CYP2C9 inhibitor	Yes
CYP2D6 inhibitor	Yes
CYP3A4 inhibitor	Yes
Log K_p (skin permeation)	-6.84 cm/s

Druglikeness	
Lipinski	Yes; 0 violation
Ghose	Yes
Veber	Yes
Egan	Yes
Muegge	Yes
Bioavailability Score	0.55

Medicinal Chemistry	
PAINS	0 alert
Brenk	0 alert
Leadlikeness	No; 2 violations: MW>350, Rotors>7
Synthetic accessibility	3.13

Molecule 15

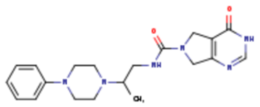

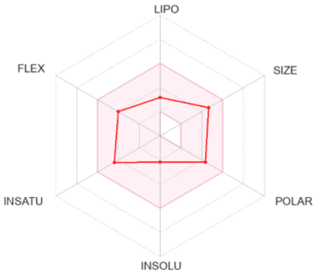

SMILES CC(N1CCN(CC1)c1ccccc1)CNC(=O)N1Cc2c(C1)nc[nH]c2=O

Physicochemical Properties	
Formula	C20H26N6O2
Molecular weight	382.46 g/mol
Num. heavy atoms	28
Num. arom. heavy atoms	12
Fraction Csp3	0.45
Num. rotatable bonds	6
Num. H-bond acceptors	4
Num. H-bond donors	2
Molar Refractivity	117.89
TPSA	84.57 Å²

Lipophilicity	
Log $P_{o/w}$ (iLOGP)	2.93
Log $P_{o/w}$ (XLOGP3)	0.07
Log $P_{o/w}$ (WLOGP)	-0.44
Log $P_{o/w}$ (MLOGP)	1.05
Log $P_{o/w}$ (SILICOS-IT)	1.24
Consensus Log $P_{o/w}$	0.97

Water Solubility	
Log *S* (ESOL)	-2.18
Solubility	2.55e+00 mg/ml ; 6.66e-03 mol/l
Class	Soluble
Log *S* (Ali)	-1.40
Solubility	1.52e+01 mg/ml ; 3.98e-02 mol/l
Class	Very soluble
Log *S* (SILICOS-IT)	-4.52
Solubility	1.15e-02 mg/ml ; 3.00e-05 mol/l
Class	Moderately soluble

Pharmacokinetics	
GI absorption	High
BBB permeant	No
P-gp substrate	Yes
CYP1A2 inhibitor	No
CYP2C19 inhibitor	No
CYP2C9 inhibitor	No
CYP2D6 inhibitor	No
CYP3A4 inhibitor	No
Log K_p (skin permeation)	-8.58 cm/s

Druglikeness	
Lipinski	Yes; 0 violation
Ghose	No; 1 violation: WLOGP<-0.4
Veber	Yes
Egan	Yes
Muegge	Yes
Bioavailability Score	0.55

Medicinal Chemistry	
PAINS	0 alert
Brenk	0 alert
Leadlikeness	No; 1 violation: MW>350
Synthetic accessibility	3.72

Molecule 16

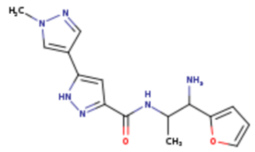

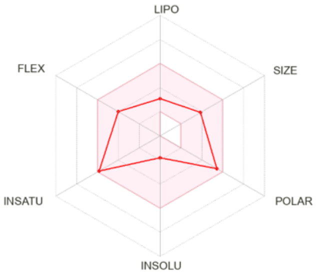

SMILES CC(C(c1ccco1)N)NC(=O)c1n[nH]c(c1)c1cnn(c1)C

Physicochemical Properties	
Formula	C15H18N6O2
Molecular weight	314.34 g/mol
Num. heavy atoms	23
Num. arom. heavy atoms	15
Fraction Csp3	0.27
Num. rotatable bonds	6
Num. H-bond acceptors	5
Num. H-bond donors	3
Molar Refractivity	83.14
TPSA	114.76 Å²

Lipophilicity	
Log $P_{o/w}$ (iLOGP)	1.44
Log $P_{o/w}$ (XLOGP3)	-0.08
Log $P_{o/w}$ (WLOGP)	0.90
Log $P_{o/w}$ (MLOGP)	-0.58
Log $P_{o/w}$ (SILICOS-IT)	0.91
Consensus Log $P_{o/w}$	0.52

Water Solubility	
Log *S* (ESOL)	-1.83
Solubility	4.70e+00 mg/ml ; 1.50e-02 mol/l
Class	Very soluble
Log *S* (Ali)	-1.88
Solubility	4.16e+00 mg/ml ; 1.32e-02 mol/l
Class	Very soluble
Log *S* (SILICOS-IT)	-3.99
Solubility	3.21e-02 mg/ml ; 1.02e-04 mol/l
Class	Soluble

Pharmacokinetics	
GI absorption	High
BBB permeant	No
P-gp substrate	Yes
CYP1A2 inhibitor	No
CYP2C19 inhibitor	No
CYP2C9 inhibitor	No
CYP2D6 inhibitor	No
CYP3A4 inhibitor	No
Log K_p (skin permeation)	-8.27 cm/s

Druglikeness	
Lipinski	Yes; 0 violation
Ghose	Yes
Veber	Yes
Egan	Yes
Muegge	Yes
Bioavailability Score	0.55

Medicinal Chemistry	
PAINS	0 alert
Brenk	0 alert
Leadlikeness	Yes
Synthetic accessibility	3.67

Molecule 17

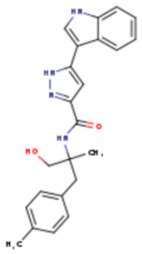

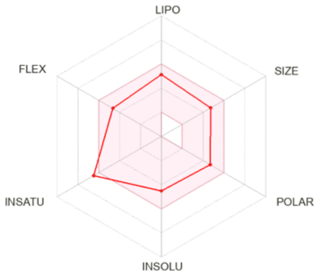

SMILES OCC(NC(=O)c1n[nH]c(c1)c1c[nH]c2c1cccc2)(Cc1ccc(cc1)C)C

Physicochemical Properties	
Formula	C23H24N4O2
Molecular weight	388.46 g/mol
Num. heavy atoms	29
Num. arom. heavy atoms	20
Fraction Csp3	0.22
Num. rotatable bonds	7
Num. H-bond acceptors	3
Num. H-bond donors	4
Molar Refractivity	113.95
TPSA	93.80 Å²

Lipophilicity	
Log $P_{o/w}$ (iLOGP)	2.93
Log $P_{o/w}$ (XLOGP3)	3.50
Log $P_{o/w}$ (WLOGP)	3.59
Log $P_{o/w}$ (MLOGP)	2.09
Log $P_{o/w}$ (SILICOS-IT)	4.80
Consensus Log $P_{o/w}$	3.38

Water Solubility	
Log *S* (ESOL)	-4.50
Solubility	1.22e-02 mg/ml ; 3.15e-05 mol/l
Class	Moderately soluble
Log *S* (Ali)	-5.15
Solubility	2.73e-03 mg/ml ; 7.03e-06 mol/l
Class	Moderately soluble
Log *S* (SILICOS-IT)	-8.14
Solubility	2.78e-06 mg/ml ; 7.16e-09 mol/l
Class	Poorly soluble

Pharmacokinetics	
GI absorption	High
BBB permeant	No
P-gp substrate	Yes
CYP1A2 inhibitor	No
CYP2C19 inhibitor	Yes
CYP2C9 inhibitor	Yes
CYP2D6 inhibitor	Yes
CYP3A4 inhibitor	Yes
Log K_p (skin permeation)	-6.18 cm/s

Druglikeness	
Lipinski	Yes; 0 violation
Ghose	Yes
Veber	Yes
Egan	Yes
Muegge	Yes
Bioavailability Score	0.55

Medicinal Chemistry	
PAINS	0 alert
Brenk	0 alert
Leadlikeness	No; 1 violation: MW>350
Synthetic accessibility	3.49

Supplemental Figure S3: LCMS characterization of the selected 17 compounds (next page)

MaxPeak: 100.00%
Ret_Time: 0.612 min

BA072969$D

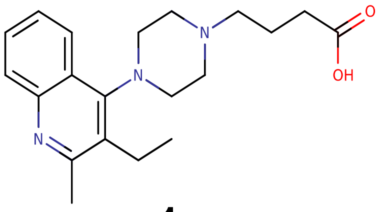

1

Mol Wt 341.45

Exact Mass 341.25

#	Time	Area%
1	0.612	100.00

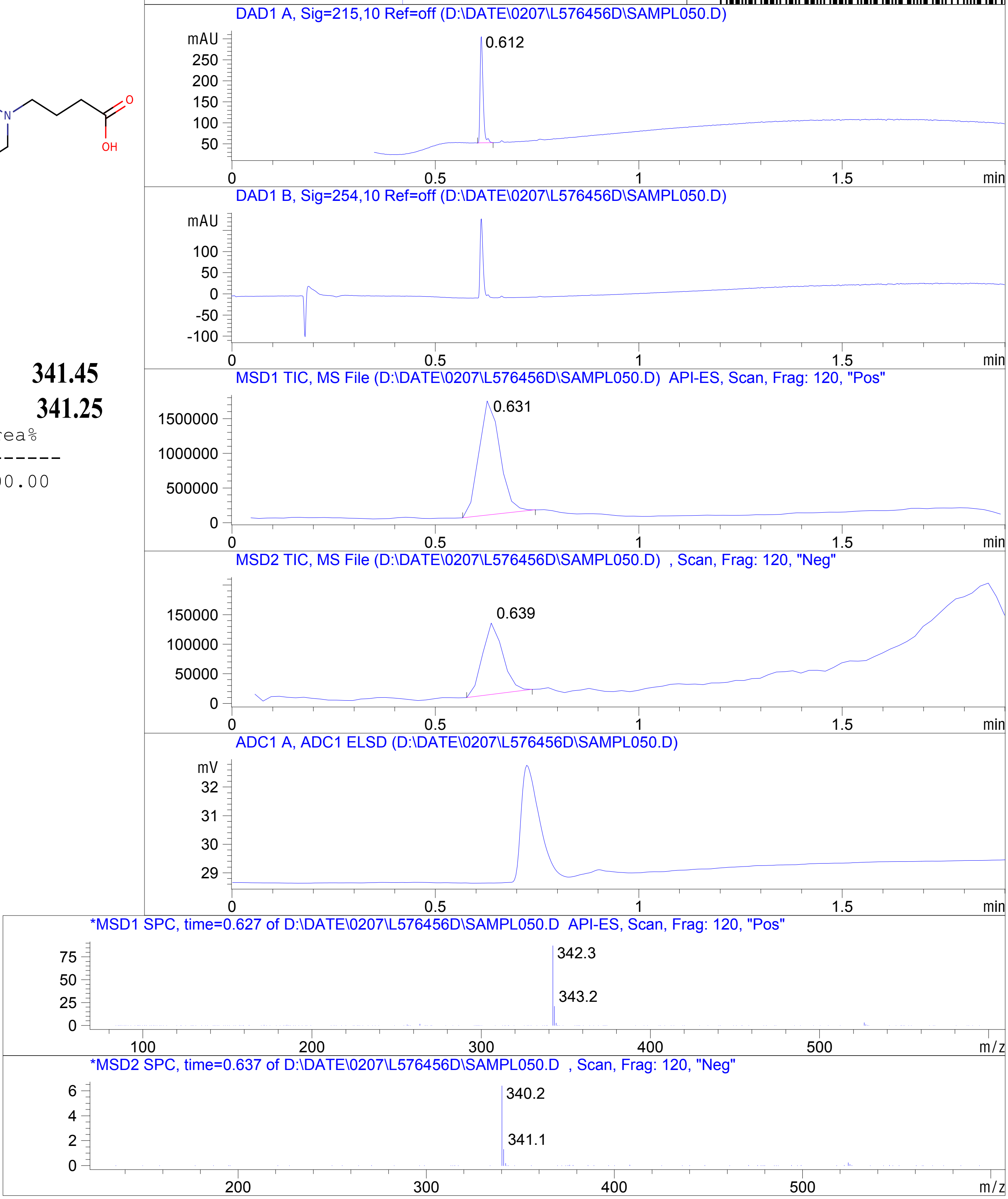

RT 0.631

RT 0.639

Inj.Date 2/8/2023 E -SL- Acq. Method C:\HPCHEM\-> ->

MaxPeak: 98.99%
Ret_Time: 0.804 min

BA072965$3

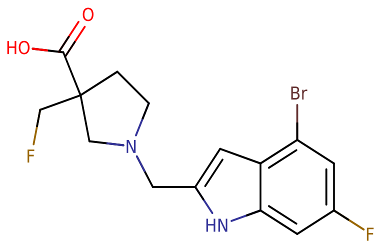

2

Mol Wt 373.19

Exact Mass 372.05

#	Time	Area%
1	0.804	98.99
2	1.033	1.01

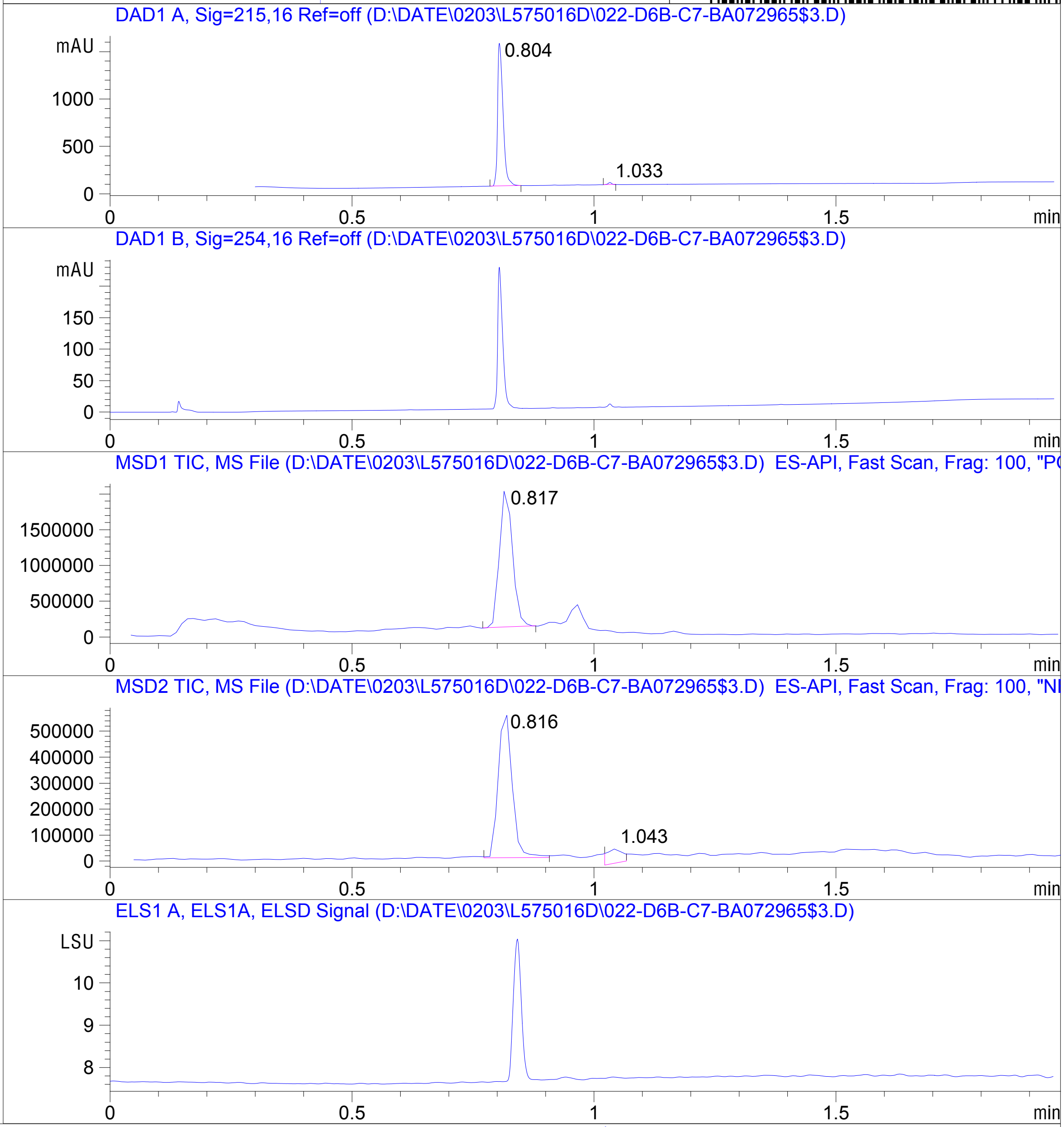

RT 0.817

RT 0.816

RT 1.043

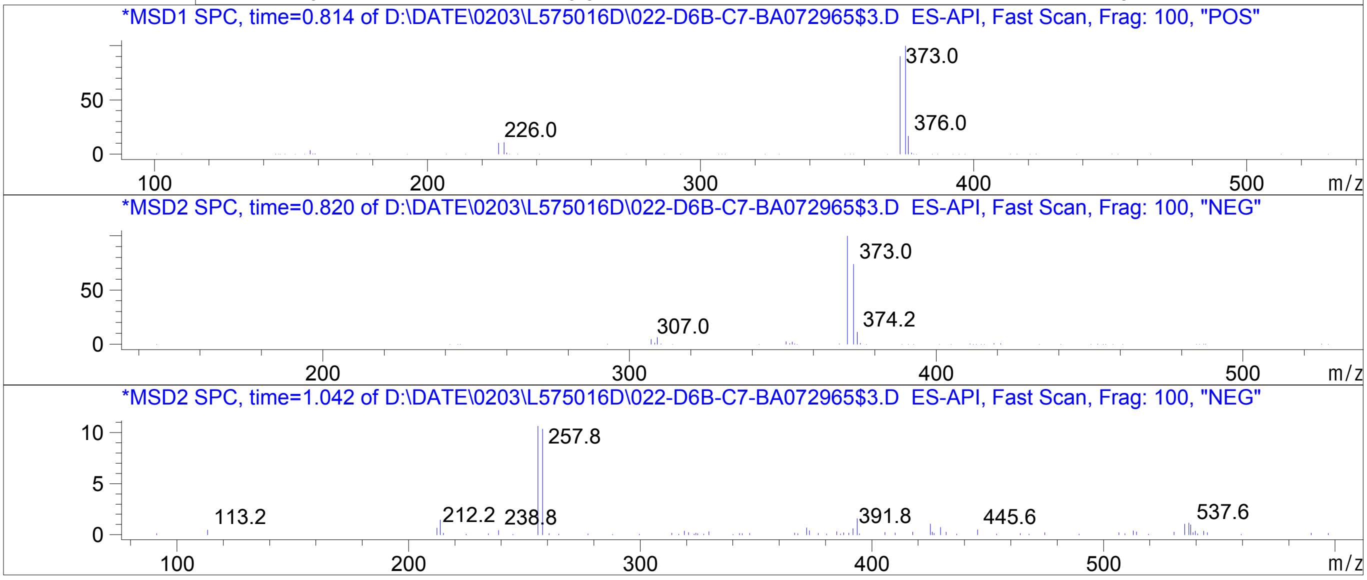

Inj.Date 2/3/2023 E Acq. Method C:\Users\ -> ->

MaxPeak: 100.00%
Ret_Time: 0.867 min

BA405922$2

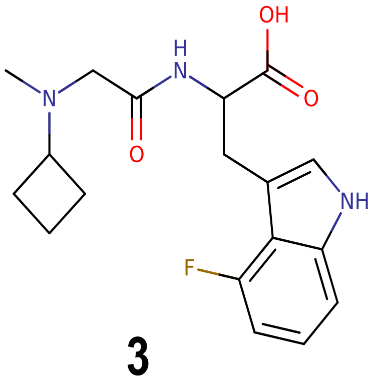

3

**Mol Wt**	**347.38**
**Exact Mass**	**347.19**

#	Time	Area%
1	0.867	100.00

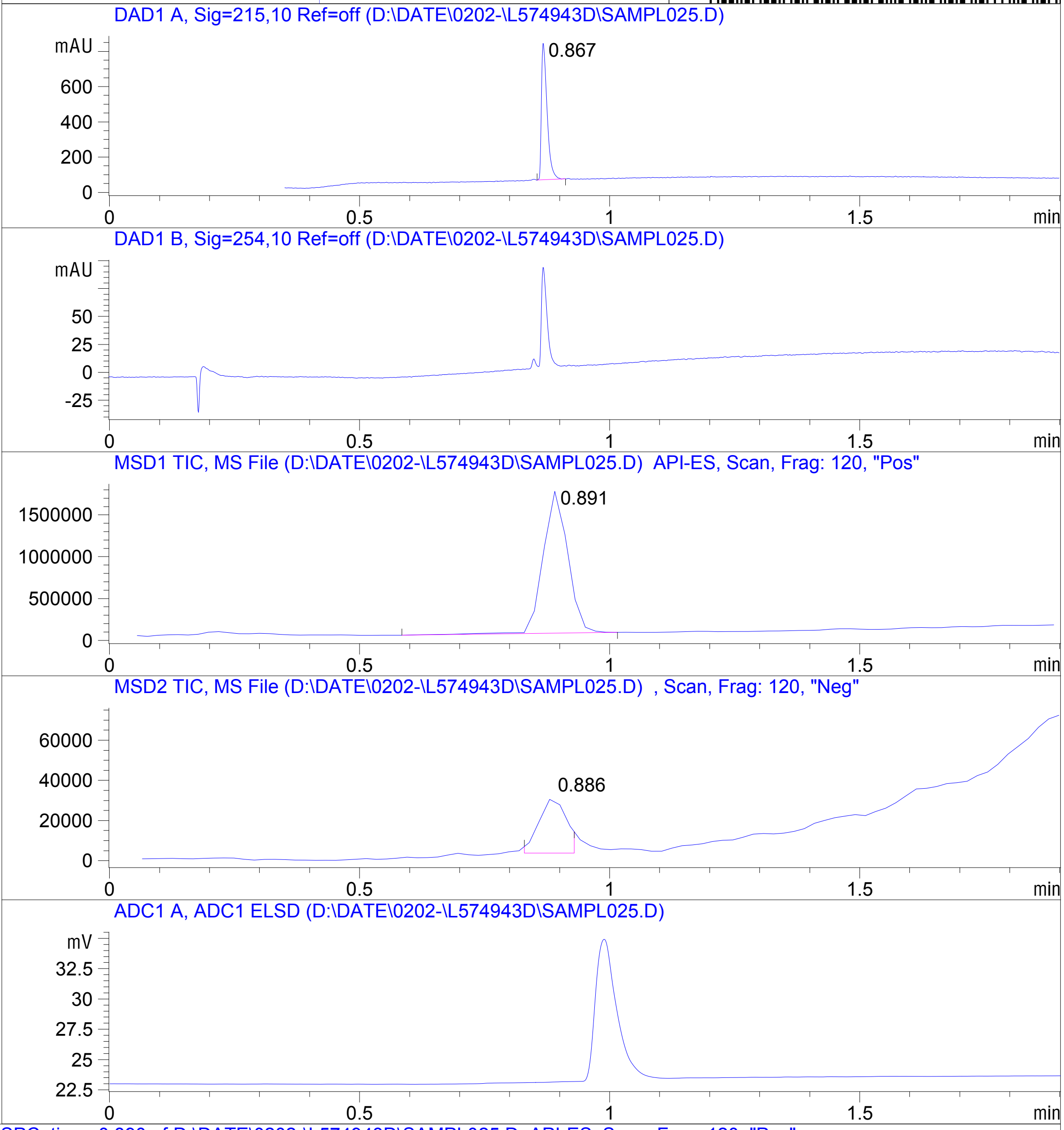

RT 0.891

RT 0.886

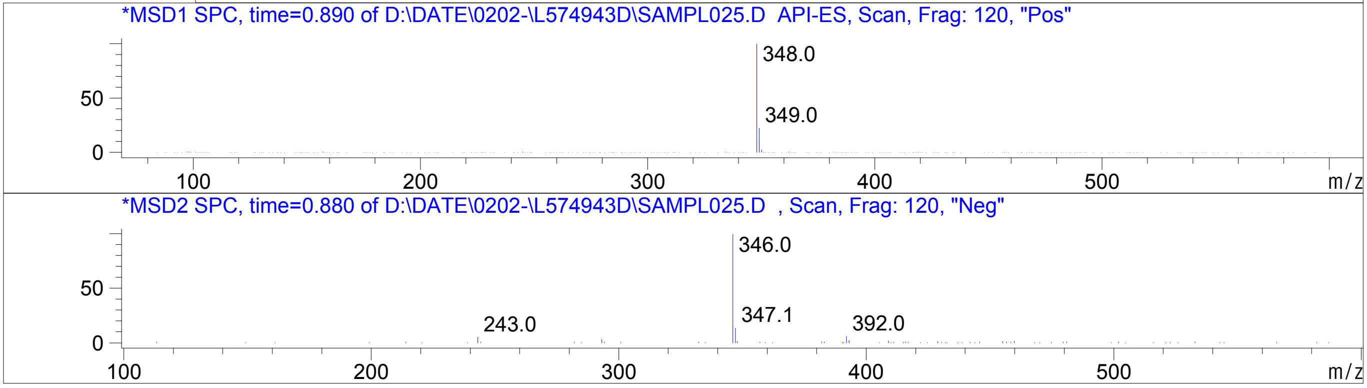

Inj.Date 2/3/2023 E -VL- Acq. Method C:\HPCHEM\-> ->

MaxPeak: 100.00%
Ret_Time: 0.772 min

BA072967$3

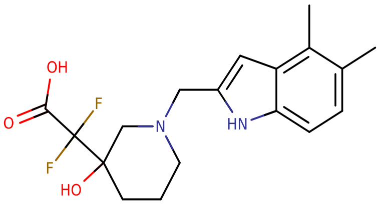

4

Mol Wt 352.38

Exact Mass 352.19

#	Time	Area%
1	0.772	100.00

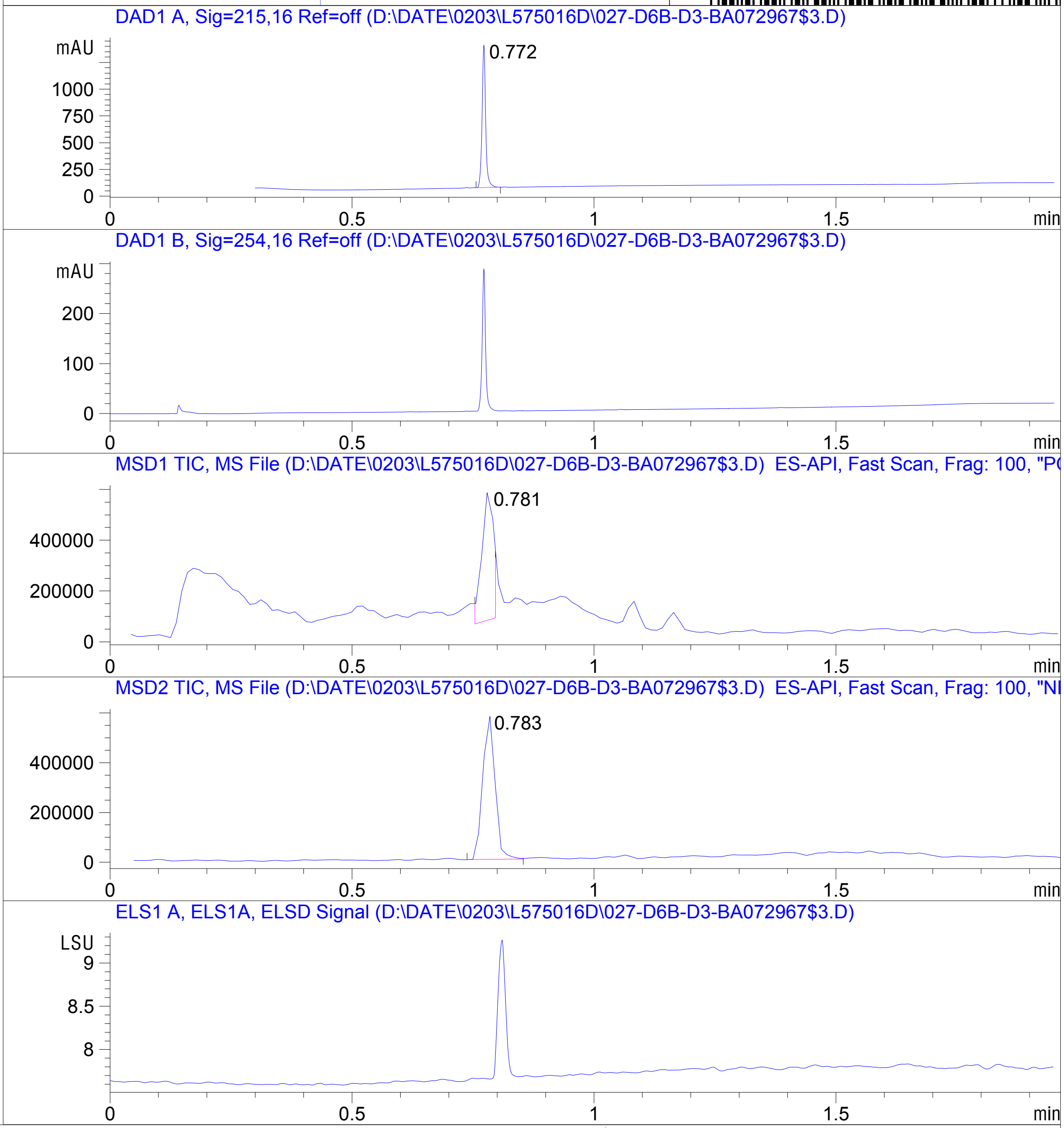

RT 0.781

RT 0.783

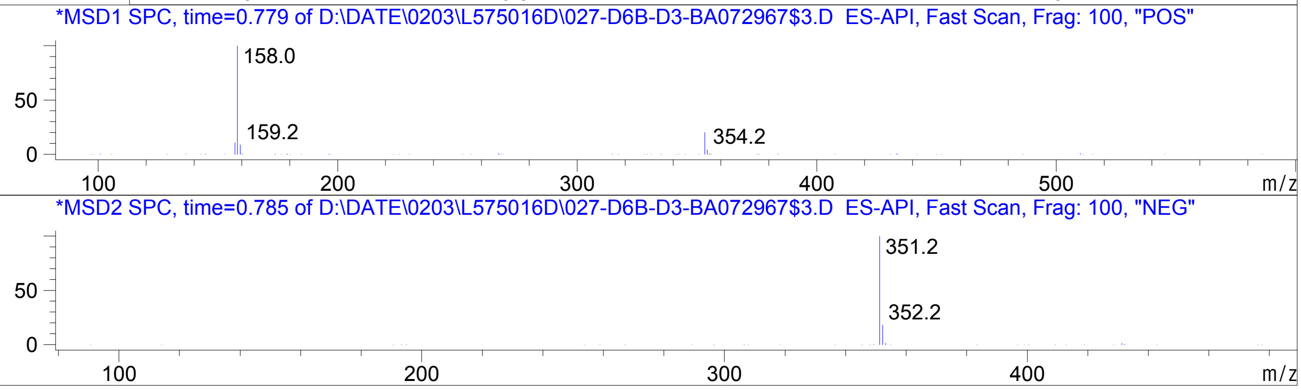

Inj.Date 2/3/2023 E Acq. Method C:\Users\ -> ->

MaxPeak: 100.00%
Ret_Time: 0.719 min

BA405923$11

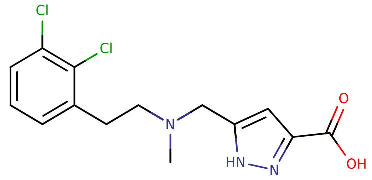

5

Mol Wt 328.19

Exact Mass 327.07

#	Time	Area%
1	0.719	100.00

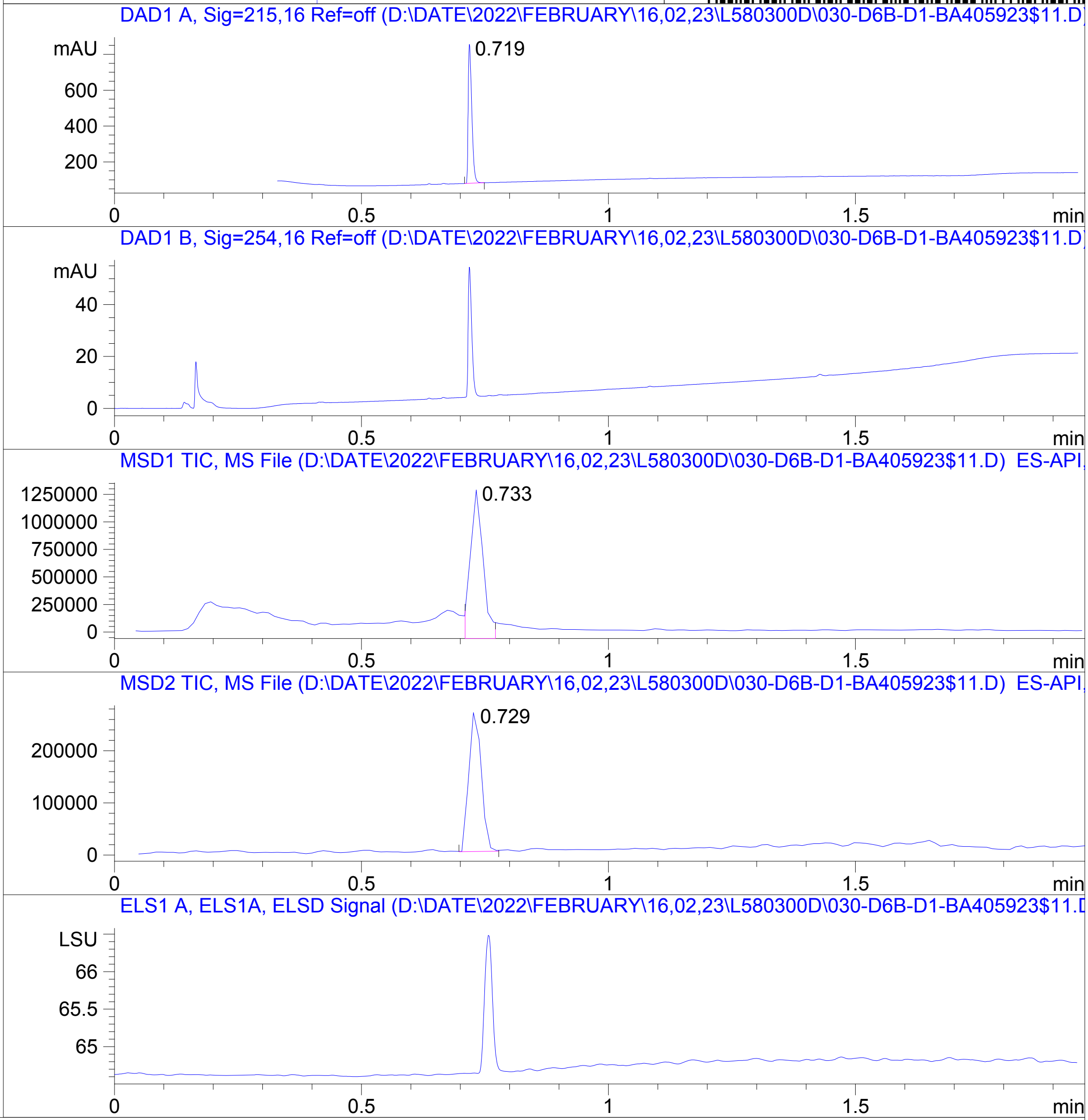

RT 0.733

RT 0.729

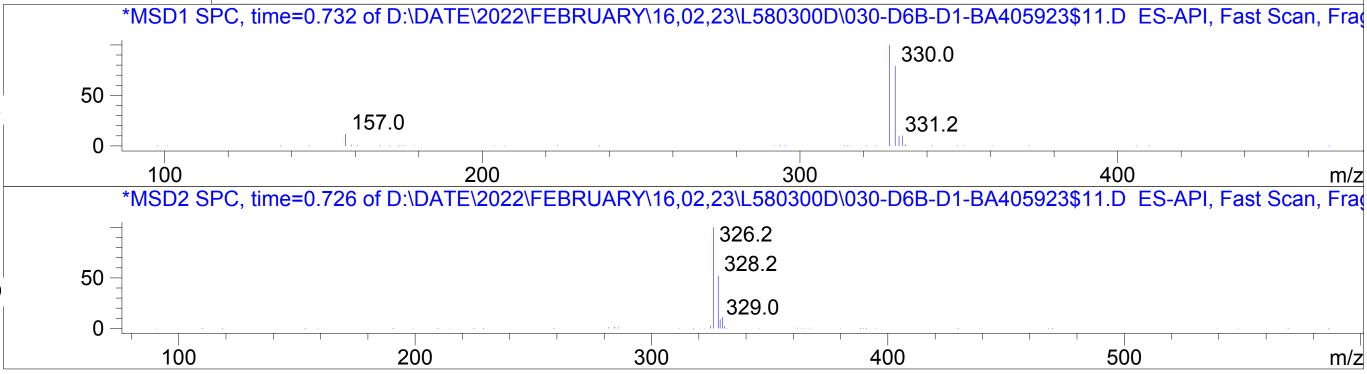

Inj.Date 2/16/2023   T   <invalid>   Acq. Method C:\Users\ ->   ->

MaxPeak: 97.55%
Ret_Time: 1.035 min

BA072962$2

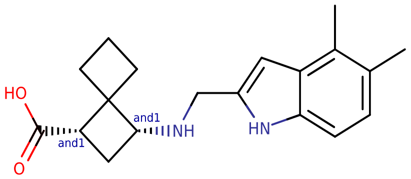

6

Mol Wt 312.41

Exact Mass 312.22

#	Time	Area%
1	1.035	97.55
2	1.075	2.45

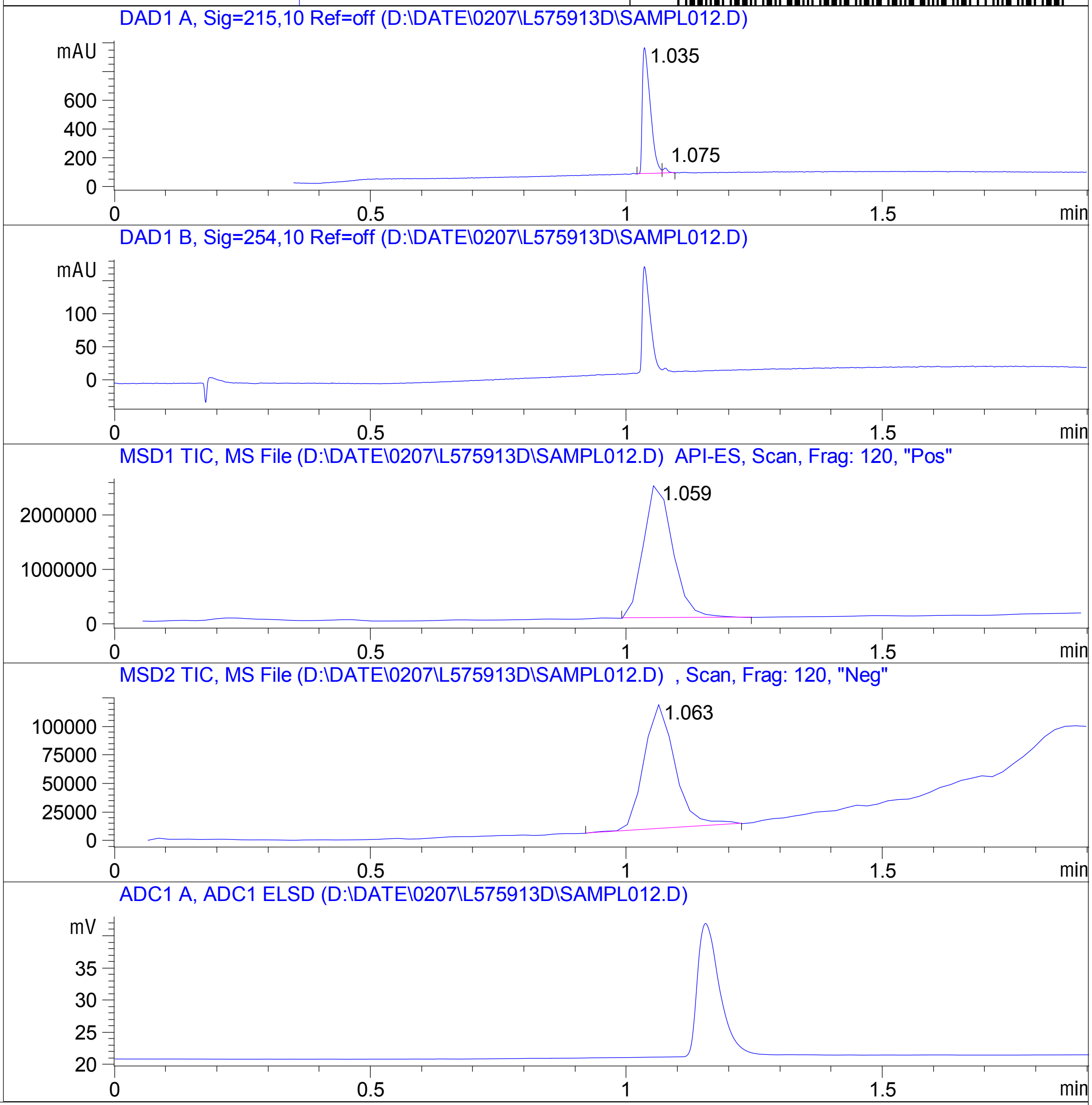

RT 1.059

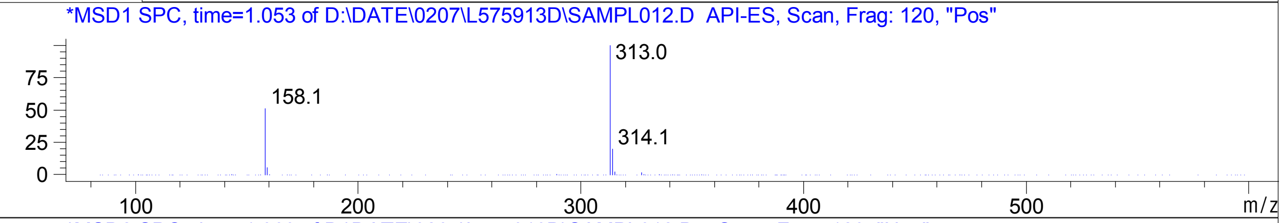

RT 1.063

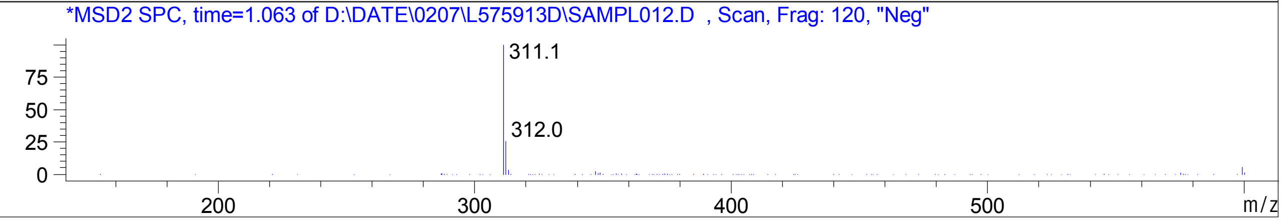

Inj.Date 2/7/2023 CH -VL- Acq. Method C:\HPCHEM\-> ->

MaxPeak: 100.00%
Ret_Time: 1.001 min

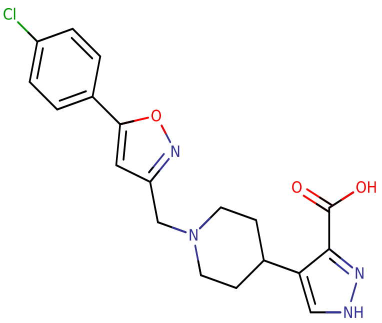

7

Mol Wt 386.83

Exact Mass 386.13

#	Time	Area%
1	1.001	100.00

BA072968$2

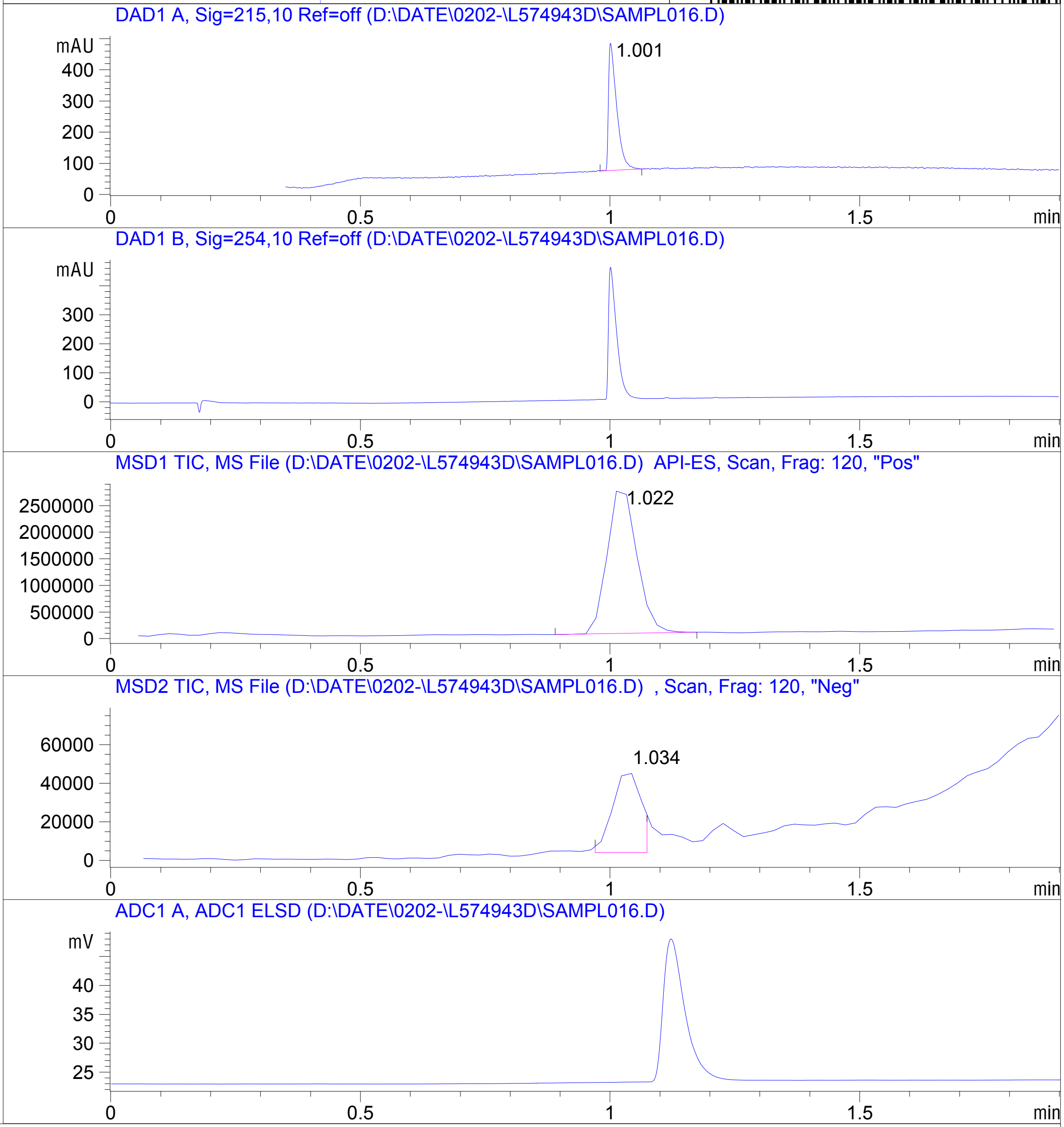

RT 1.022

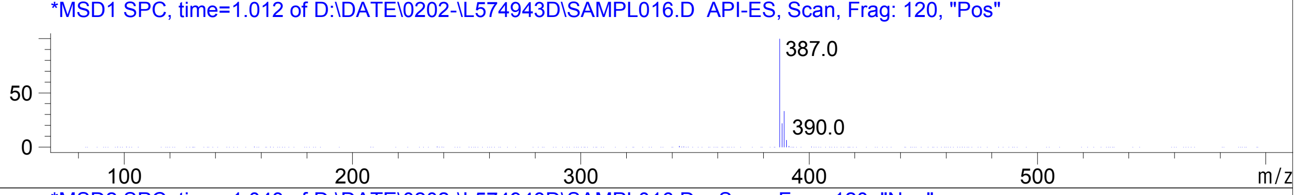

RT 1.034

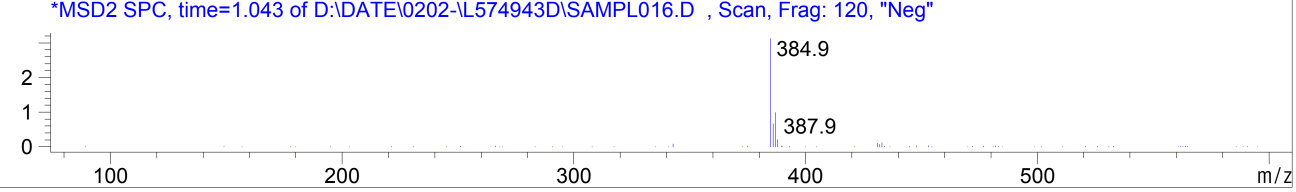

Inj.Date 2/3/2023 E -VL- Acq. Method C:\HPCHEM\-> ->

MaxPeak: 96.47%
Ret_Time: 0.993 min

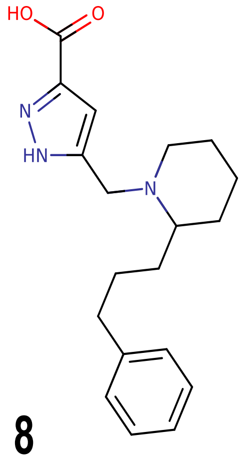

8

Mol Wt 327.42

Exact Mass 327.23

#	Time	Area%
1	0.993	96.47
2	1.123	3.53

BA072961$1

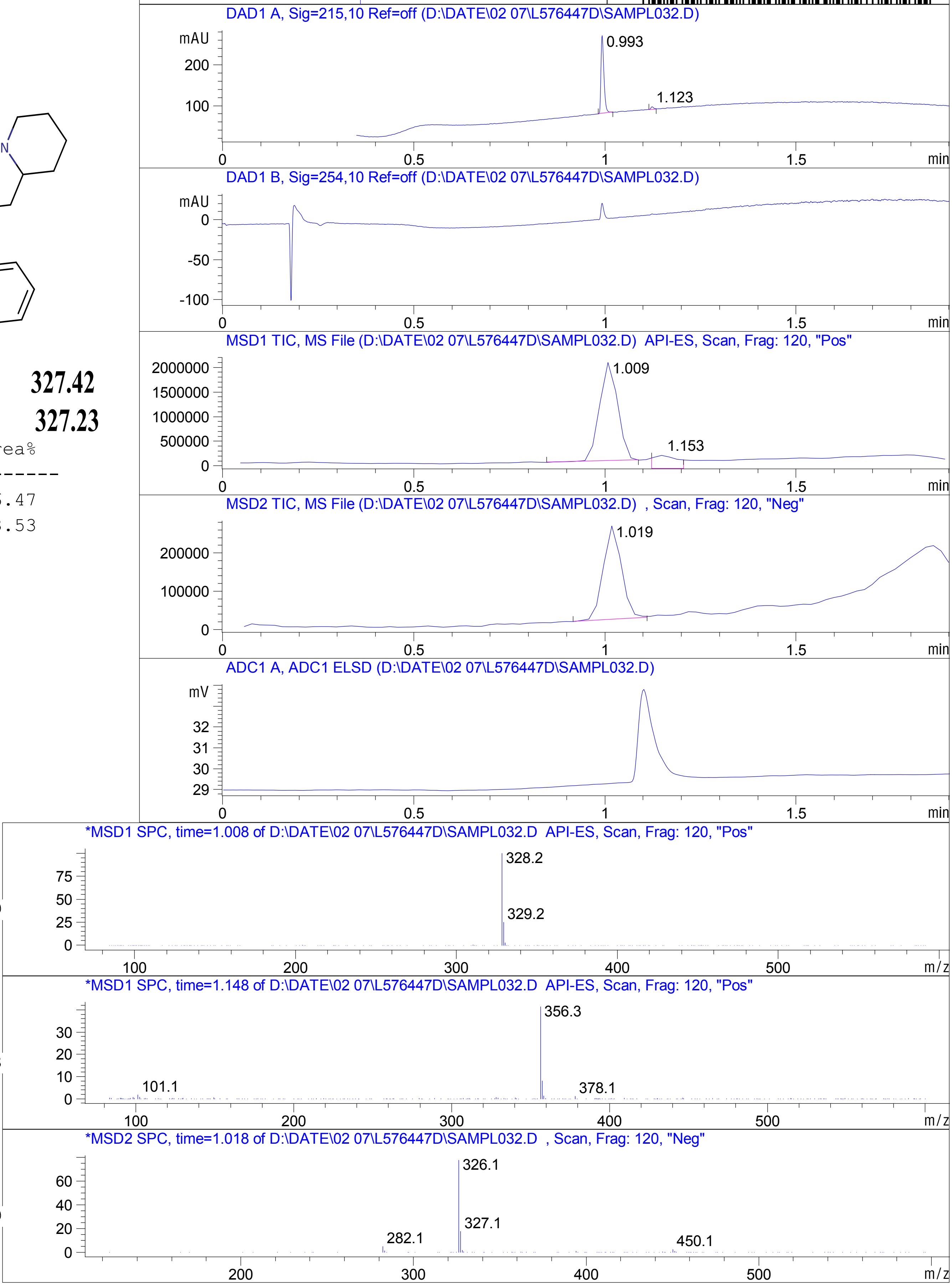

Inj.Date 2/8/2023 CH -SL- Acq. Method C:\HPCHEM\-> ->

BA405921$1

MaxPeak: 96.73%
Ret_Time: 1.025 min

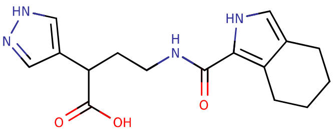

9

Mol Wt 316.36

Exact Mass 316.17

#	Time	Area%
1	0.932	3.27
2	1.025	96.73

RT 1.047

RT 1.046

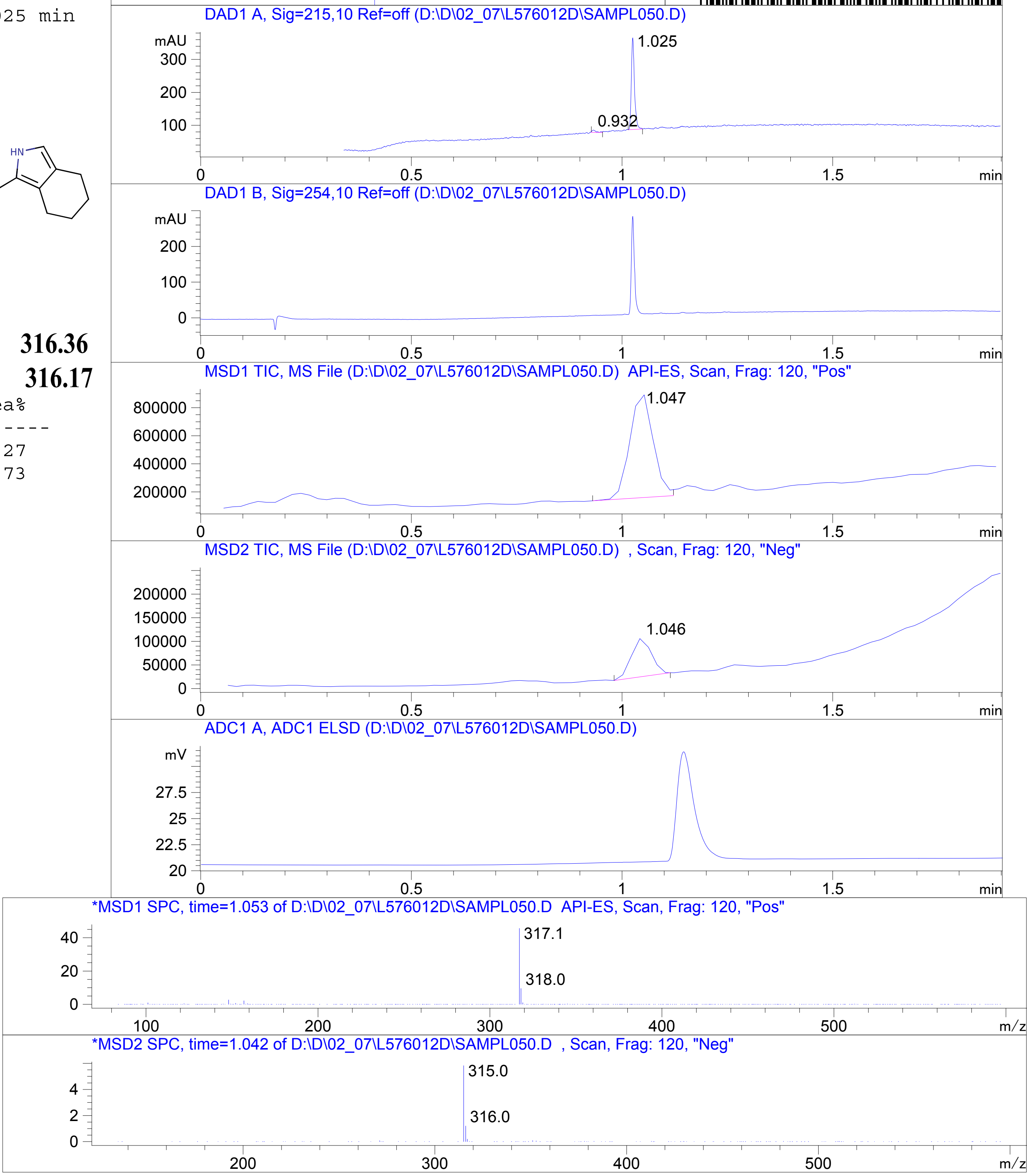

Inj.Date 2/7/2023 N -VL- Acq. Method C:\HPCHEM\-> ->

MaxPeak: 96.91%
Ret_Time: 0.870 min

BA370145$1

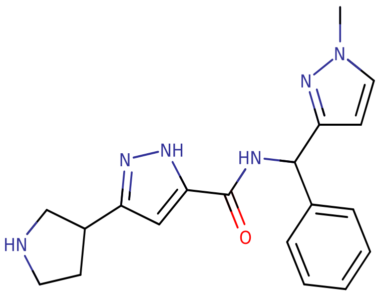

10

Mol Wt 350.42

Exact Mass 350.21

#	Time	Area%
1	0.870	96.91
2	0.923	3.09

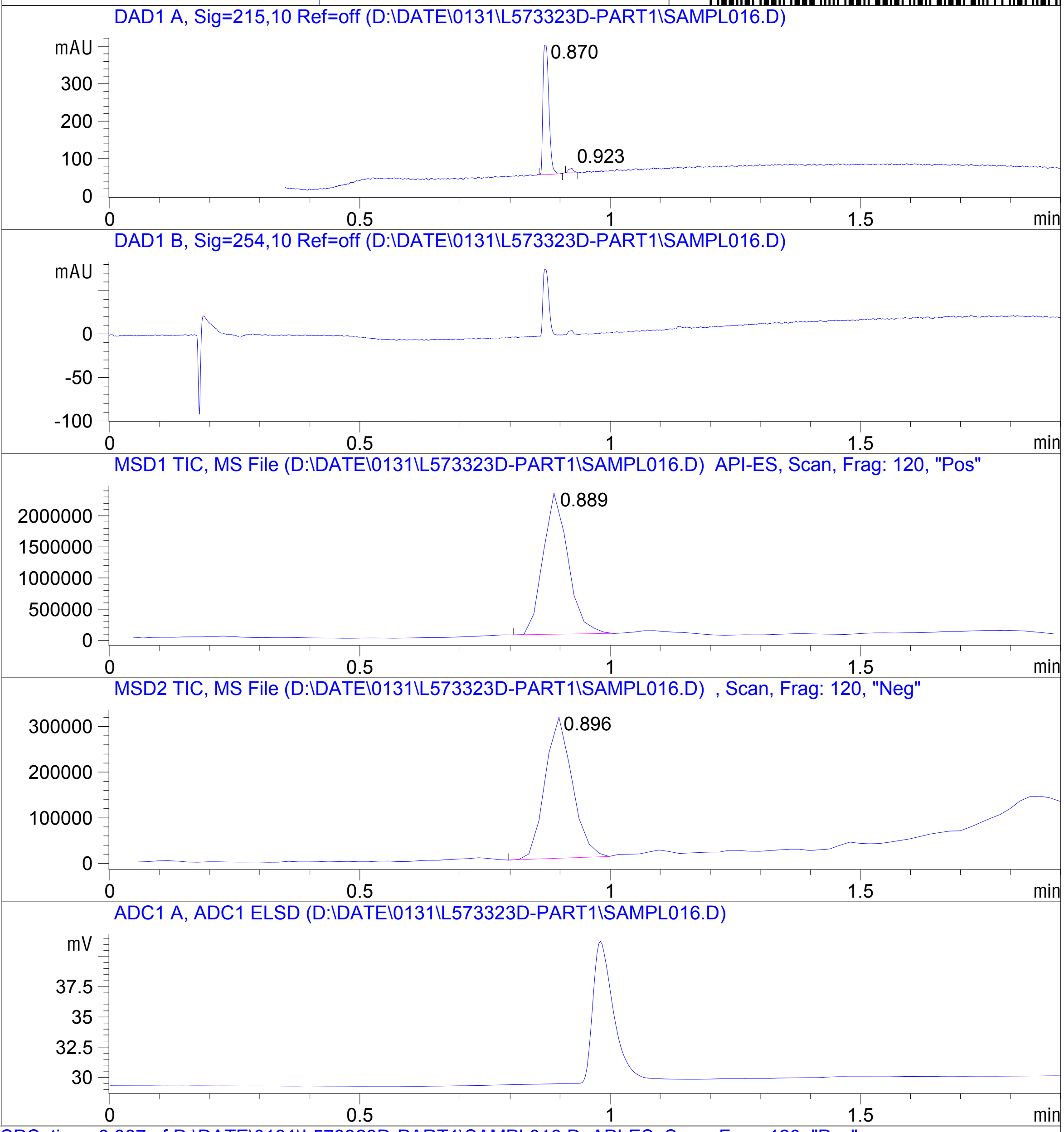

RT 0.889

RT 0.896

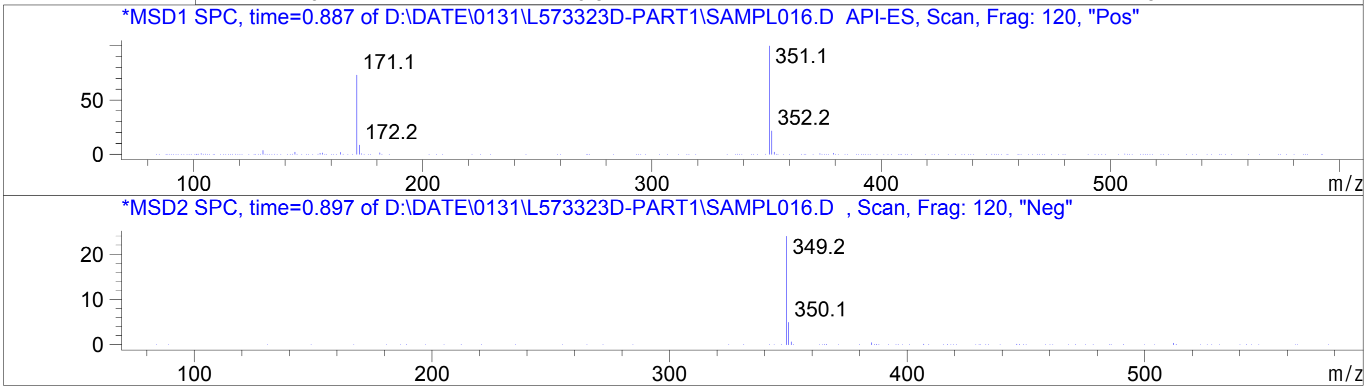

Inj.Date 1/31/2023 E -SL- Acq. Method C:\HPCHEM\-> ->

MaxPeak: 98.26%
Ret_Time: 0.922 min

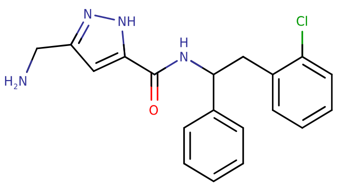

11

Mol Wt 354.83
Exact Mass 354.15

#	Time	Area%
1	0.922	98.26
2	0.983	1.74

BA370150$2

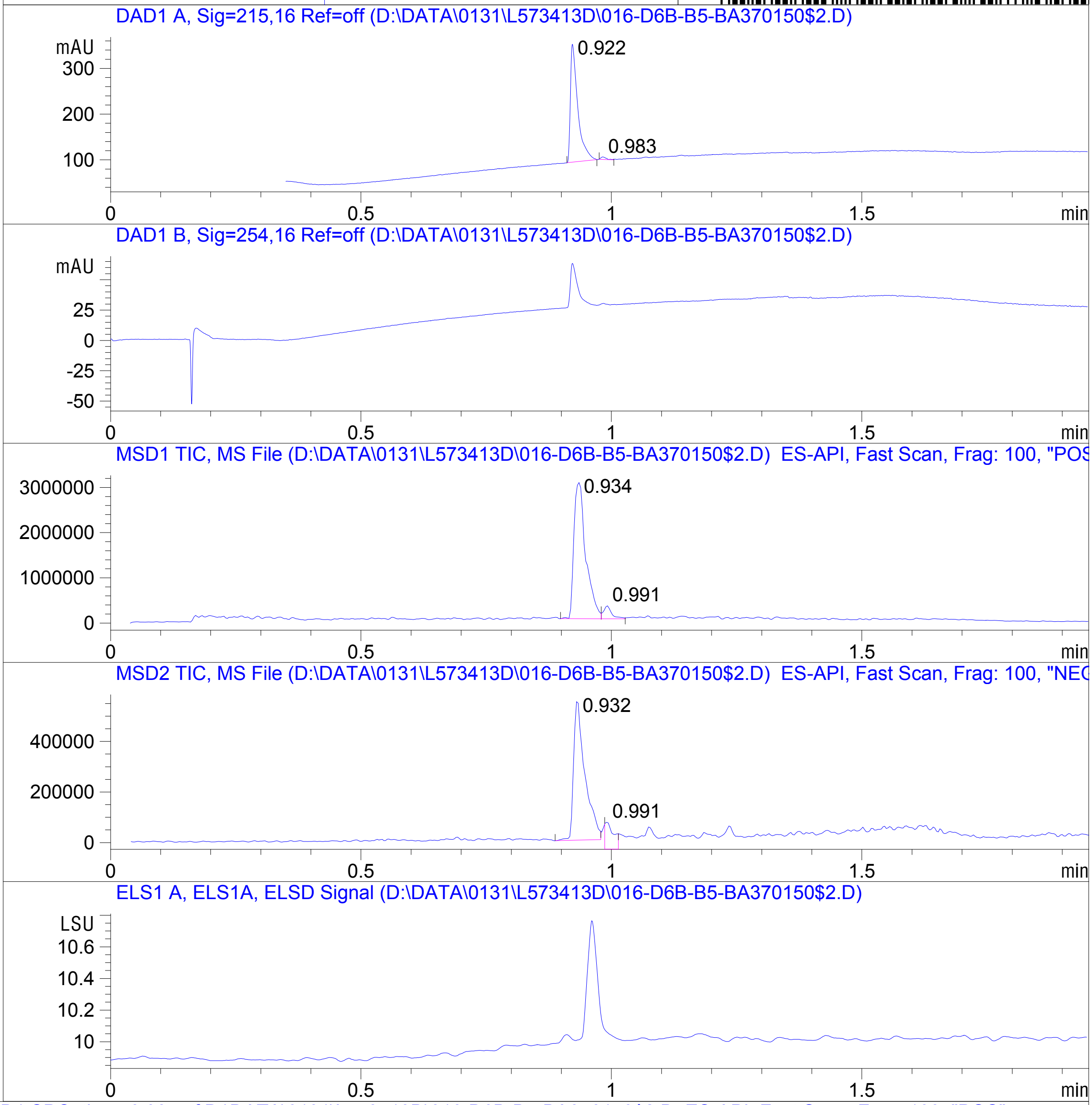

RT 0.934

RT 0.991

RT 0.932

RT 0.991

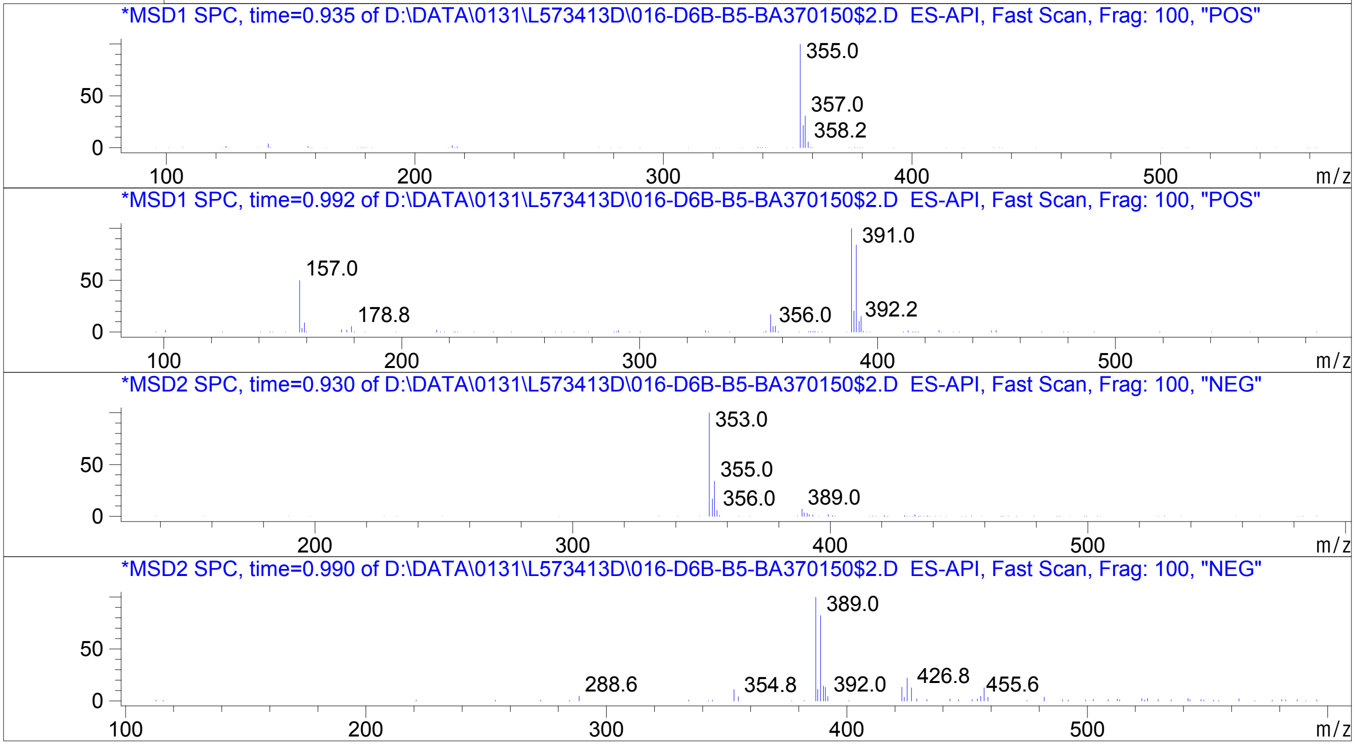

Inj.Date 1/30/2023 OA Acq. Method C:\Users\ -> ->

MaxPeak: 100.00%
Ret_Time: 0.869 min

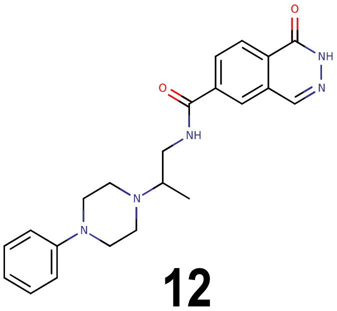

12

Mol Wt 391.47

Exact Mass 391.23

#	Time	Area%
1	0.869	100.00

BA370146$13

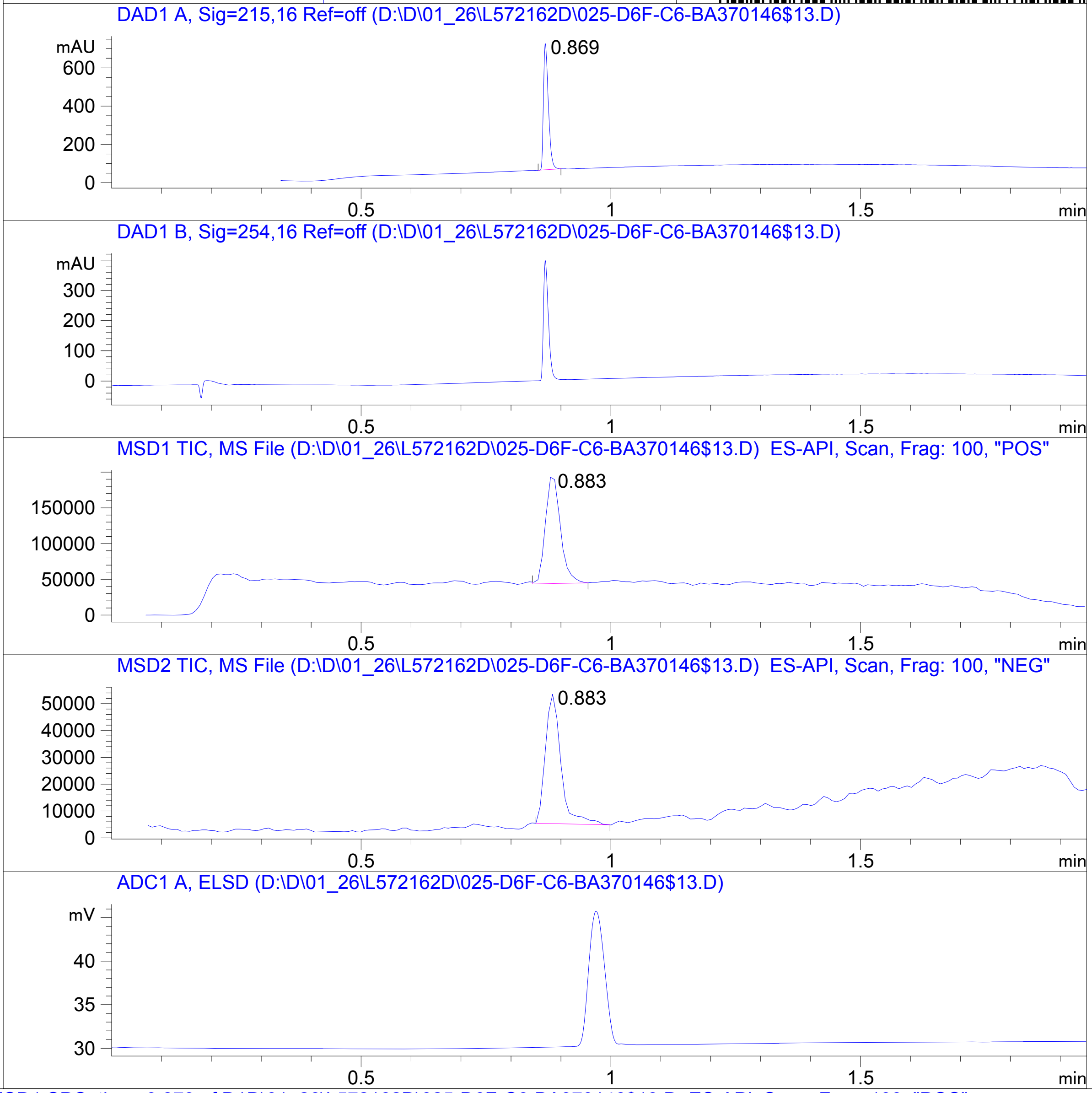

RT 0.883

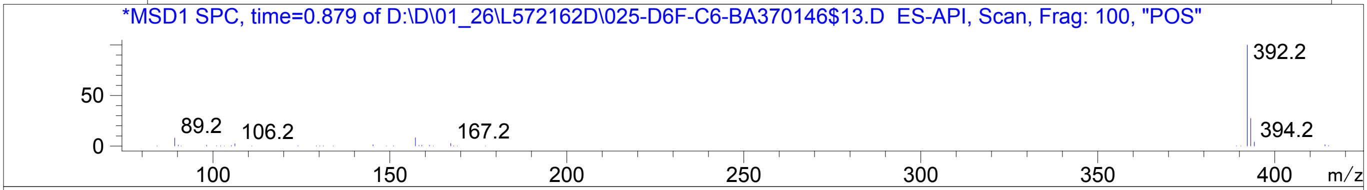

RT 0.883

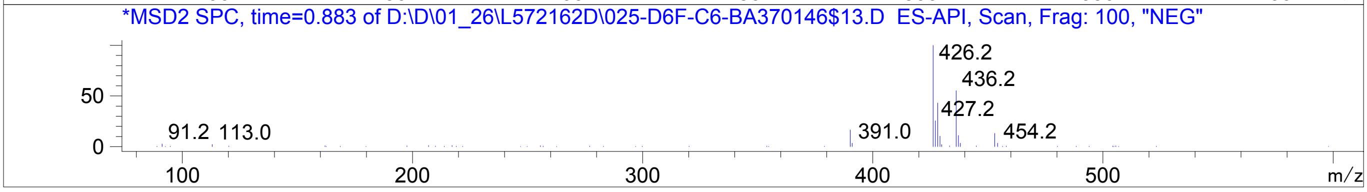

MaxPeak: 100.00%
Ret_Time: 0.607 min

BA370147$3

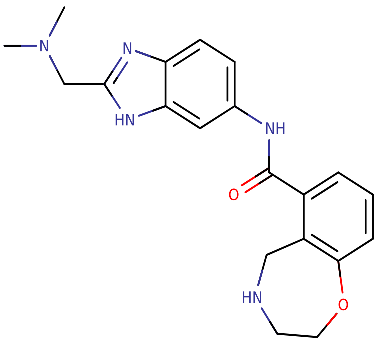

13

Mol Wt 365.43

Exact Mass 365.21

#	Time	Area%
1	0.607	100.00

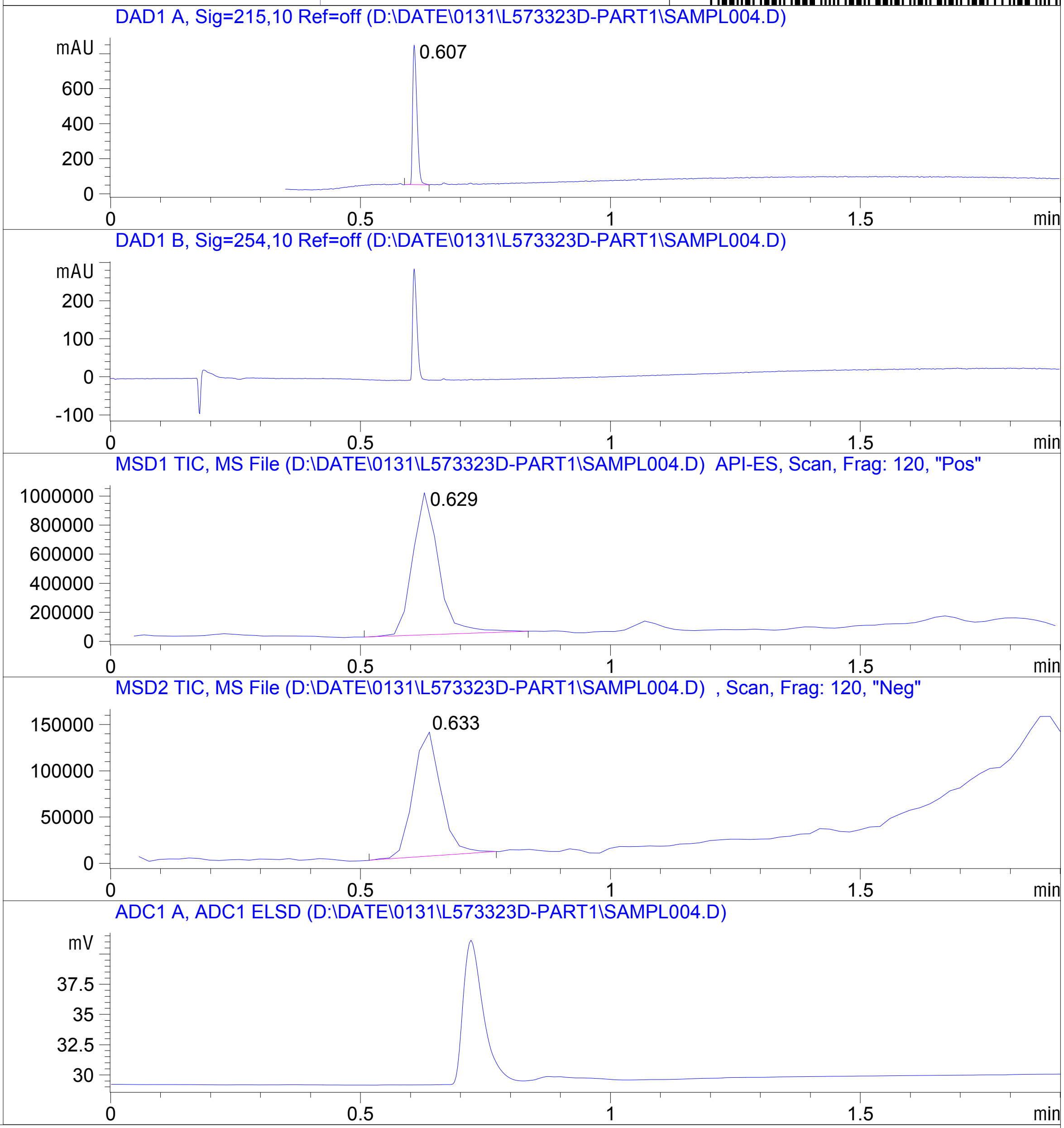

RT 0.629

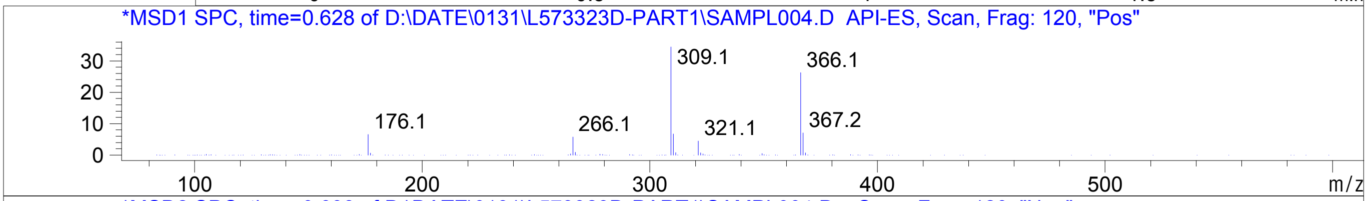

RT 0.633

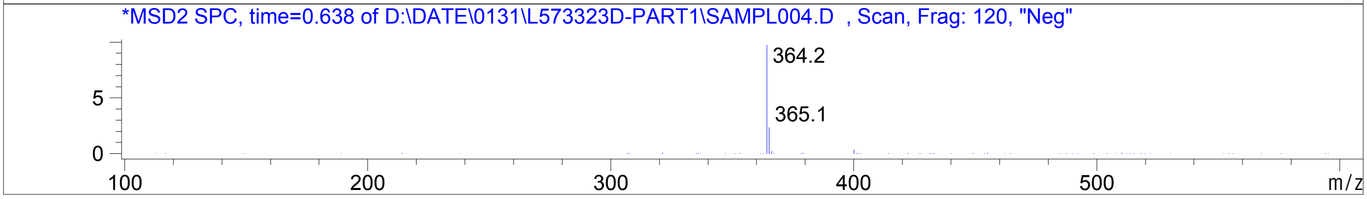

Inj.Date 1/31/2023 E -SL- Acq. Method C:\HPCHEM\-> ->

BA370151$3

```
MaxPeak:  100.00%
Ret_Time: 0.779 min
```

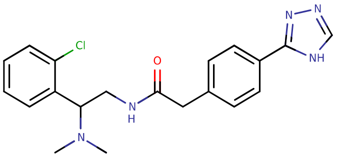

14

Mol Wt 383.88

Exact Mass 383.18

```
#  Time   Area%
------------------
1  0.779  100.00
```

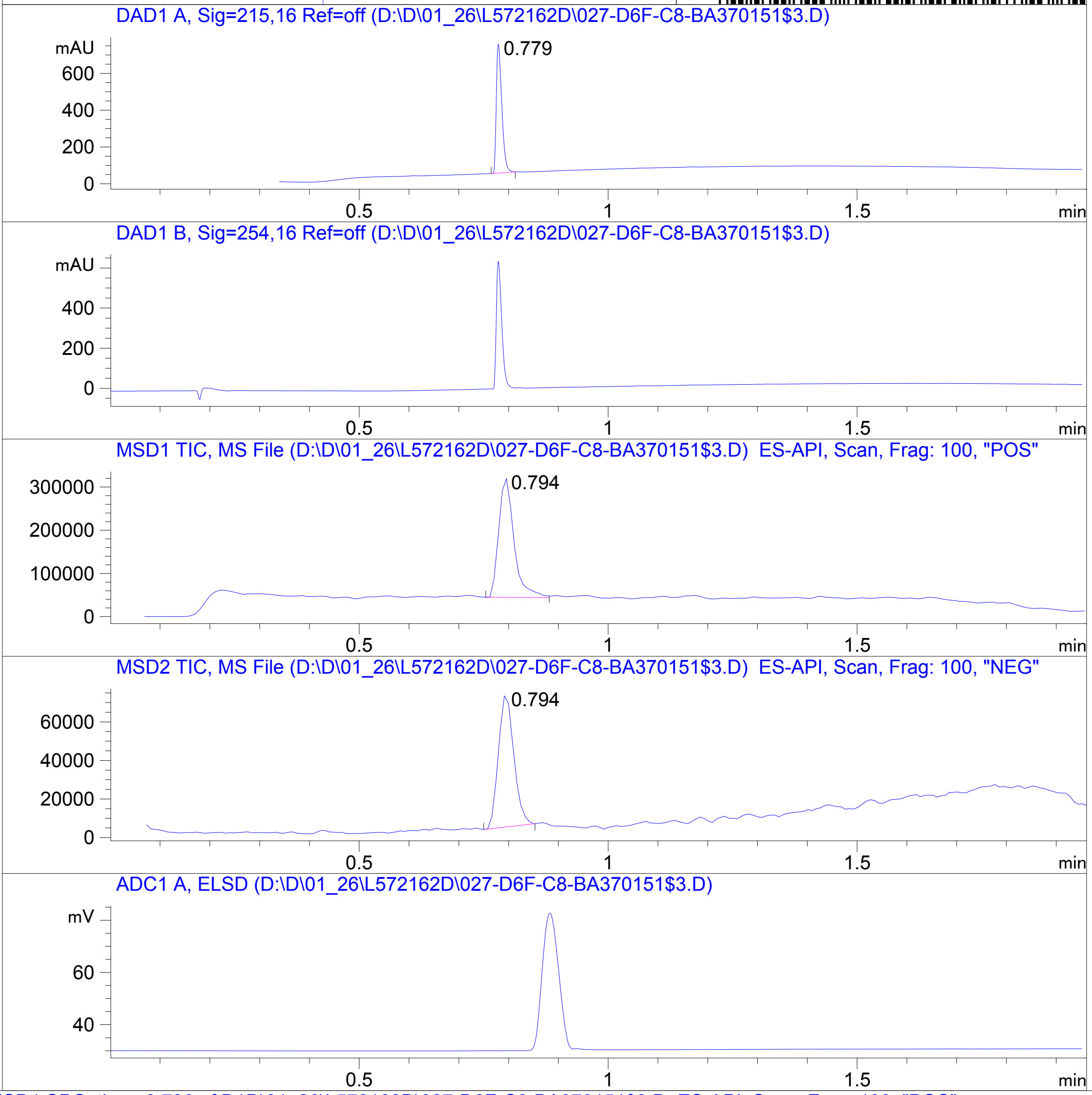

RT 0.794

RT 0.794

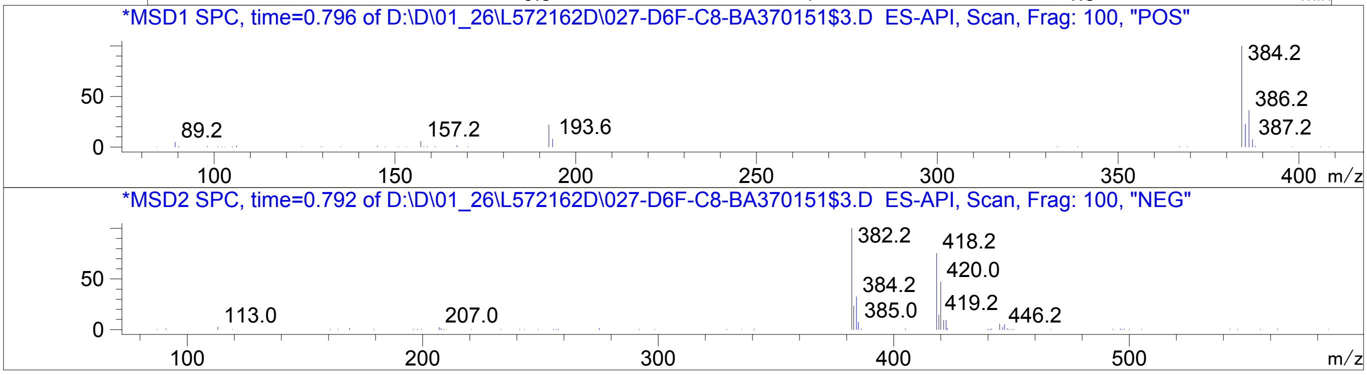

MaxPeak: 100.00%
Ret_Time: 0.797 min

BA370148$1

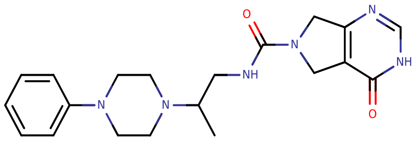

15

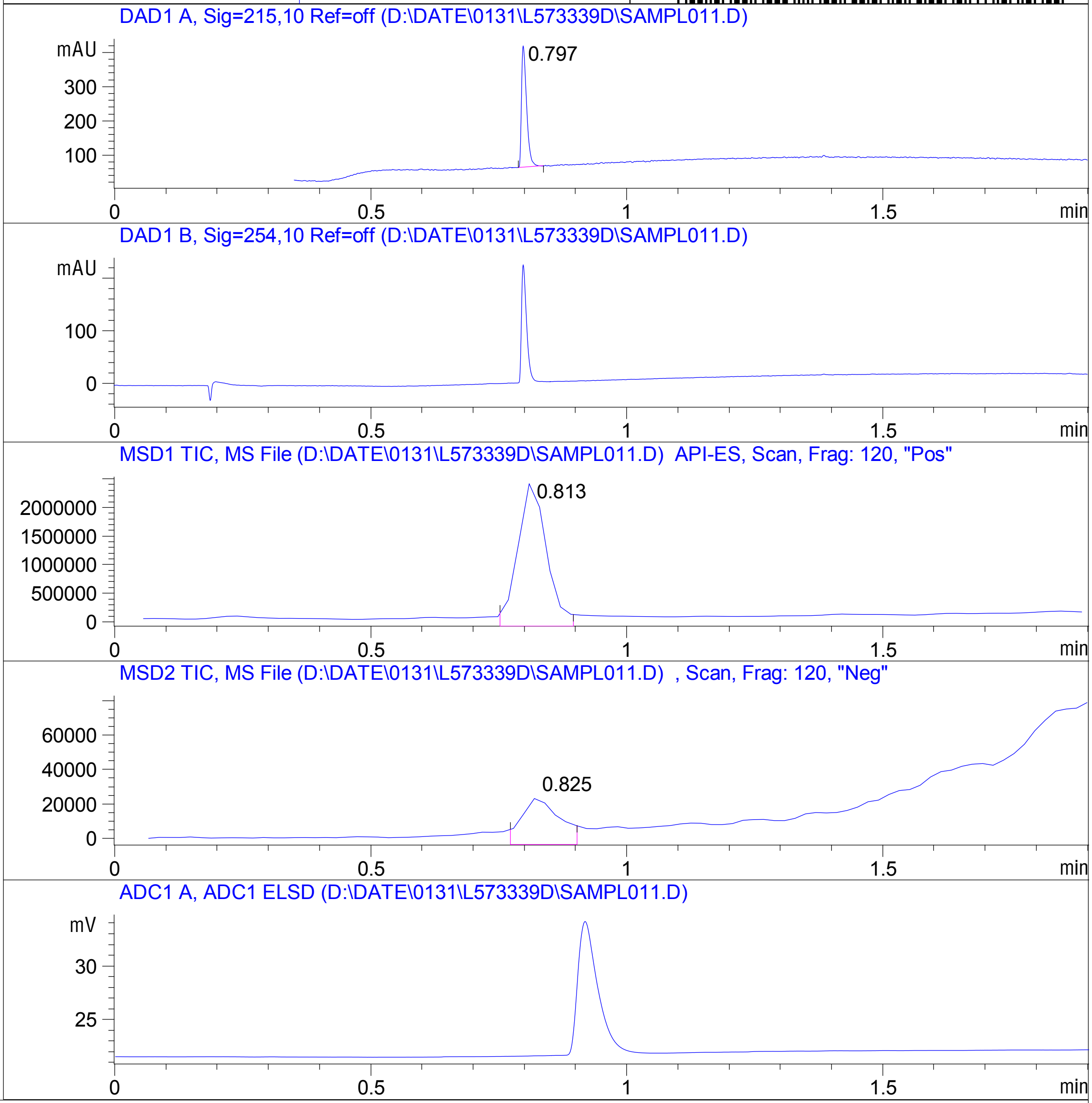

Mol Wt 382.46

Exact Mass 382.24

#	Time	Area%
1	0.797	100.00

RT 0.813

RT 0.825

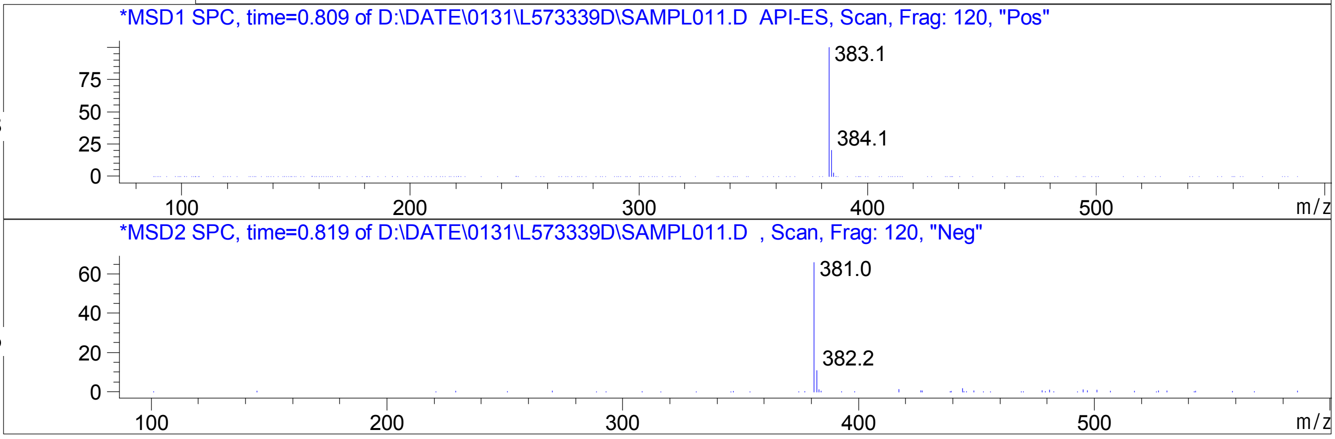

Inj.Date 1/31/2023 CH -VL- Acq. Method C:\HPCHEM\-> ->

MaxPeak: 53.03%
Ret_Time: 0.786 min

BA370152$2

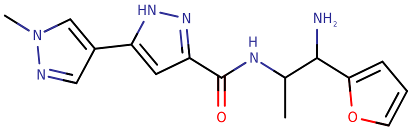

16

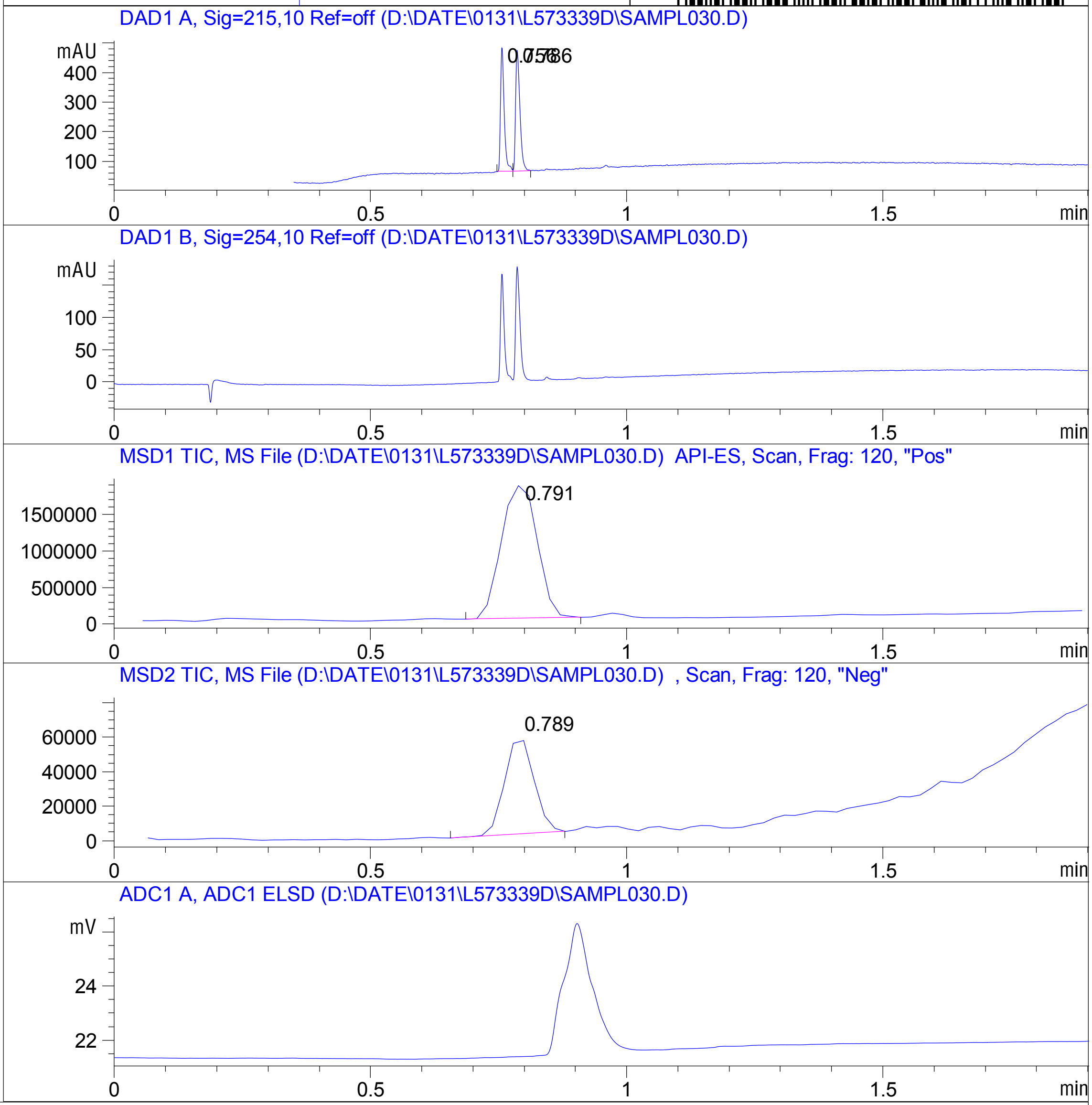

Mol Wt 314.34

Exact Mass 314.16

#	Time	Area%
1	0.756	46.97
2	0.786	53.03

RT 0.791

RT 0.789

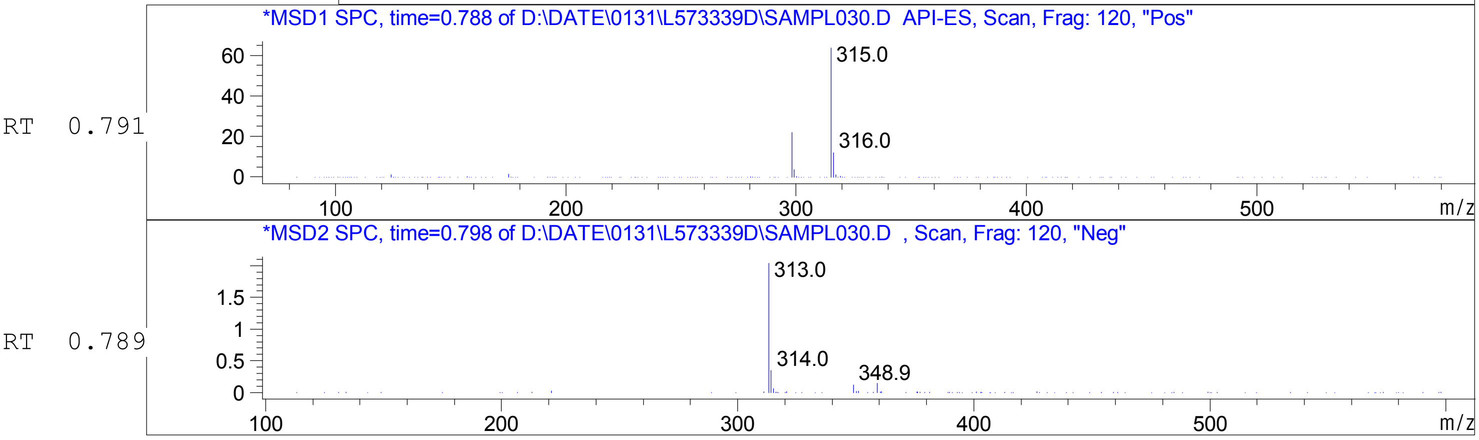

Inj.Date 1/31/2023 CH -VL- Acq. Method C:\HPCHEM\-> ->

MaxPeak: 98.46%
Ret_Time: 1.315 min

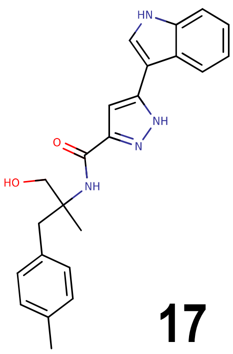

17

Mol Wt 388.46

Exact Mass 388.22

#	Time	Area%
1	1.181	1.54
2	1.315	98.46

BA370149$2

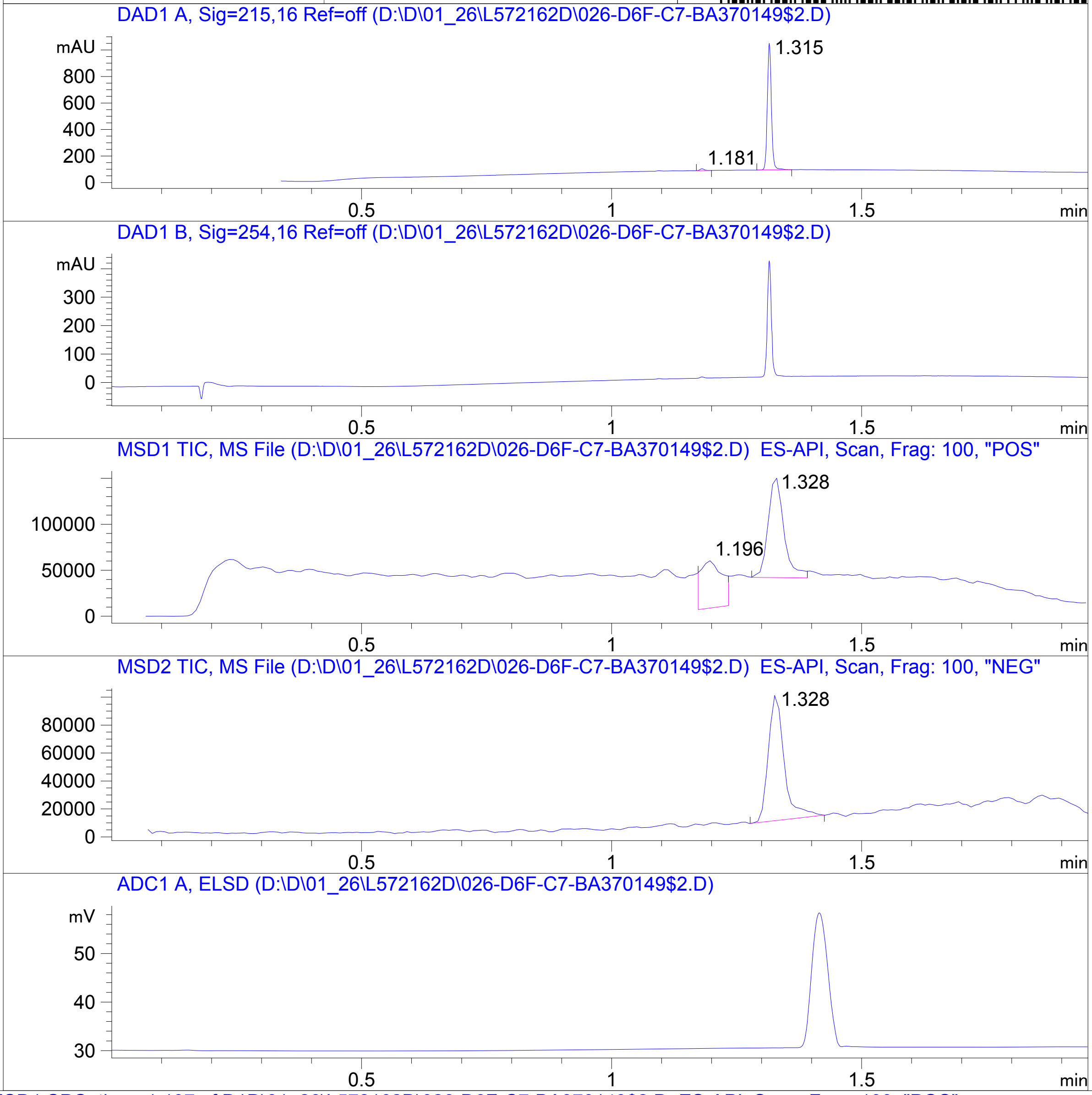

RT 1.196

RT 1.328

RT 1.328

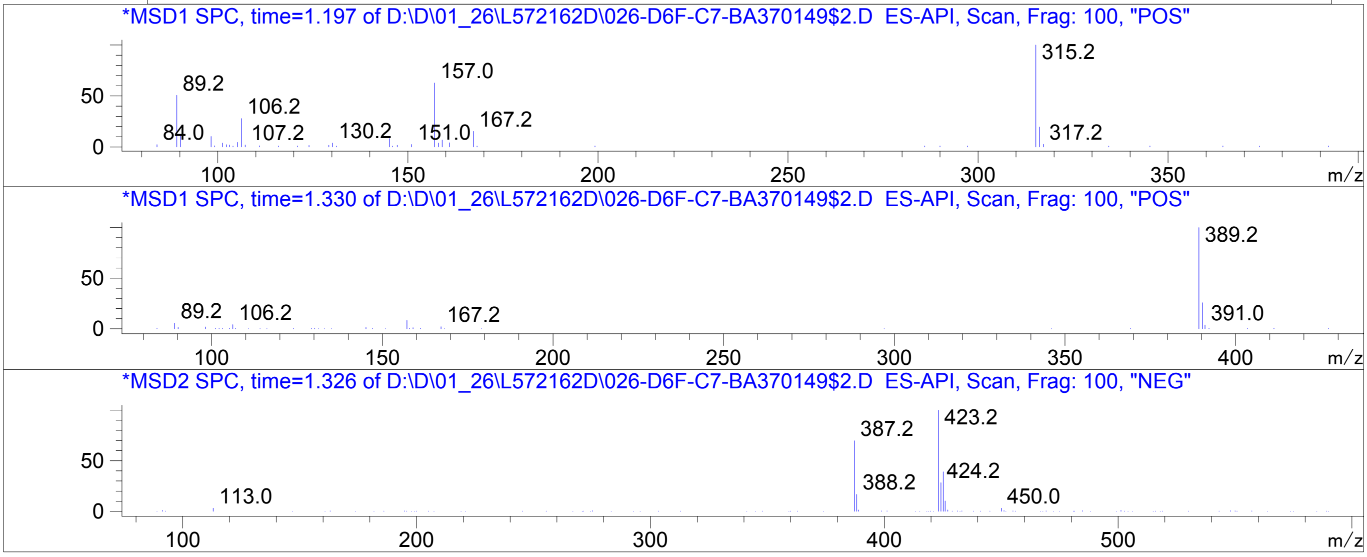

Supplement: Supplementary file 1 — ao5c01169_si_001.pdf [file ao5c01169_si_001.pdf]
